# Reinforcement learning with associative or discriminative generalization across states and actions: fMRI at 3 T and 7 T

**DOI:** 10.1002/hbm.25988

**Published:** 2022-07-21

**Authors:** Jaron T. Colas, Neil M. Dundon, Raphael T. Gerraty, Natalie M. Saragosa‐Harris, Karol P. Szymula, Koranis Tanwisuth, J. Michael Tyszka, Camilla van Geen, Harang Ju, Arthur W. Toga, Joshua I. Gold, Dani S. Bassett, Catherine A. Hartley, Daphna Shohamy, Scott T. Grafton, John P. O'Doherty

**Affiliations:** ^1^ Department of Psychological and Brain Sciences University of California Santa Barbara California USA; ^2^ Division of the Humanities and Social Sciences California Institute of Technology Pasadena California USA; ^3^ Computation and Neural Systems Program, California Institute of Technology Pasadena California USA; ^4^ Department of Child and Adolescent Psychiatry, Psychotherapy, and Psychosomatics University of Freiburg Freiburg im Breisgau Germany; ^5^ Department of Psychology Columbia University New York New York USA; ^6^ Zuckerman Mind Brain Behavior Institute, Columbia University New York New York USA; ^7^ Center for Science and Society Columbia University New York New York USA; ^8^ Department of Psychology New York University New York New York USA; ^9^ Department of Psychology University of California Los Angeles California USA; ^10^ Department of Bioengineering University of Pennsylvania Philadelphia Pennsylvania USA; ^11^ Department of Psychology University of California Berkeley California USA; ^12^ Department of Psychology University of Pennsylvania Philadelphia Pennsylvania USA; ^13^ Neuroscience Graduate Group University of Pennsylvania Philadelphia Pennsylvania USA; ^14^ Laboratory of Neuro Imaging USC Stevens Neuroimaging and Informatics Institute, Keck School of Medicine of USC, University of Southern California Los Angeles California USA; ^15^ Department of Neuroscience University of Pennsylvania Philadelphia Pennsylvania USA; ^16^ Department of Electrical and Systems Engineering University of Pennsylvania Philadelphia Pennsylvania USA; ^17^ Department of Neurology University of Pennsylvania Philadelphia Pennsylvania USA; ^18^ Department of Psychiatry University of Pennsylvania Philadelphia Pennsylvania USA; ^19^ Department of Physics and Astronomy University of Pennsylvania Philadelphia Pennsylvania USA; ^20^ Santa Fe Institute Santa Fe New Mexico USA; ^21^ Center for Neural Science New York University New York New York USA; ^22^ Kavli Institute for Brain Science Columbia University New York New York USA

**Keywords:** cognitive map, counterfactual learning, dopaminergic midbrain, generalization, hippocampus, individual differences, model‐free and model‐based, multifield fMRI, reinforcement learning, striatum

## Abstract

The model‐free algorithms of “reinforcement learning” (RL) have gained clout across disciplines, but so too have model‐based alternatives. The present study emphasizes other dimensions of this model space in consideration of associative or discriminative generalization across states and actions. This “generalized reinforcement learning” (GRL) model, a frugal extension of RL, parsimoniously retains the single reward‐prediction error (RPE), but the scope of learning goes beyond the experienced state and action. Instead, the generalized RPE is efficiently relayed for bidirectional counterfactual updating of value estimates for other representations. Aided by structural information but as an implicit rather than explicit cognitive map, GRL provided the most precise account of human behavior and individual differences in a reversal‐learning task with hierarchical structure that encouraged inverse generalization across both states and actions. Reflecting inference that could be true, false (i.e., overgeneralization), or absent (i.e., undergeneralization), state generalization distinguished those who learned well more so than action generalization. With high‐resolution high‐field fMRI targeting the dopaminergic midbrain, the GRL model's RPE signals (alongside value and decision signals) were localized within not only the striatum but also the substantia nigra and the ventral tegmental area, including specific effects of generalization that also extend to the hippocampus. Factoring in generalization as a multidimensional process in value‐based learning, these findings shed light on complexities that, while challenging classic RL, can still be resolved within the bounds of its core computations.

## Graphical abstract

This “generalized reinforcement learning” (GRL) model, a frugal extension of RL, parsimoniously retains the single reward‐prediction error (RPE), but the scope of learning goes beyond the experienced state and action. Aided by structural information but as an implicit rather than explicit cognitive map, GRL provided the most precise account of human behavior and individual differences in a reversal‐learning task with hierarchical structure that encouraged inverse generalization across both states and actions. With high‐resolution high‐field fMRI targeting the dopaminergic midbrain, the GRL model's RPE signals (alongside value and decision signals) were localized within not only the striatum but also the substantia nigra and the ventral tegmental area, including specific effects of generalization that also extend to the hippocampus.

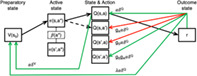

## 1 INTRODUCTION

“Reinforcement learning” (RL) is a successful computational framework for describing the means by which an agent can learn from feedback in their environment to select actions that maximize future reward. This framework has been canonized not only in machine learning and artificial intelligence (Bertsekas & Tsitsiklis, 1996; Sutton & Barto, 1998) but also in psychology (Bush & Mosteller, 1951a; Rescorla & Wagner, 1972) and neuroscience (Montague et al., 1996; Schultz, 2015; Schultz et al., 1997). In computational modeling of the nervous system, the discovery that the phasic activity of dopamine neurons represents the signature reward‐prediction error (RPE) has firmly placed RL at the core of our understanding of the neurobiological basis of reward‐related learning. Yet, as canonical RL models of the model‐free variety have proliferated to rise to the challenges of learning, so too have model‐based alternatives to the RL framework as well as dual‐systems models that are both model‐free and model‐based (Daw et al., 2005; Doll et al., 2012; O'Doherty et al., 2017, 2021). It is compellingly intuitive to consider these counterparts in terms of a straightforward dichotomy: model‐free versus model‐based. Toward the simpler end of the spectrum, model‐free processes entail implicit caching of learned associations; toward the more complex end of the spectrum, model‐based processes instead construct explicit cognitive models of the environment (which are incidentally agnostic with respect to conscious awareness). On the other hand, the present study expands this model space by introducing two additional dichotomies in their own right: associative versus discriminative generalization and state versus action generalization.

In applying RL to neurobiology, previous approaches have typically treated states and actions in the world as independent events, such that knowledge acquired about one state or action does not inform the agent about other states or actions. However, virtually all dynamic environments in the real world can be characterized by connections and patterns in relational structure across events that are disconnected not only temporally but also episodically, where “episodes” here correspond to perceived groupings within a sequence (and presently discrete trials within the experiment). As an agent grapples with uncertainty, such structured interdependence means that information obtained about one state or action can provide more general knowledge about other states and actions as well. A prototypical solution for leveraging the additional information is counterfactual inference. The credit assignment of standard RL models does not account for this interdependence or for mechanisms mediating the generalization it may support.

The present study operationalizes the concepts of associative versus discriminative generalization in relation to implicitly inferential counterfactual learning (cf. Aquino et al., 2020; Balcarras & Womelsdorf, 2016; Ballard et al., 2019; Baram et al., 2021; Charpentier et al., 2020; Collette et al., 2017; Daw & Shohamy, 2008; Gläscher et al., 2009; Hampton et al., 2007; Hauser et al., 2014, 2015; Lesage & Verguts, 2021; Liu et al., 2021; Matsumoto et al., 2007; Mattar & Daw, 2018; Reiter et al., 2017; Vinckier et al., 2016; Wimmer et al., 2012; Zaki et al., 2016) that also differs with respect to states versus actions. The simpler associative generalization treats different representations as if they were equivalent or at least similar, which can but does not necessarily imply inference. In contrast, the more complex discriminative generalization treats different representations as if they were linked but specifically not equivalent—effectively implying a sort of emergent model (or cognitive map) with more abstract credit assignment. Being semi‐inferential, this counterfactual learning entails value updating that occurs without direct observation and so requires an internal representation of generalizable structure in the environment. Yet the influence of such an internal representation does not necessarily require a (cognitive) model‐based process in the stricter senses of the term; inference of that level of comprehensiveness can be achieved with alternative algorithms that are also investigated here. Rather, fundamentally model‐free signals could more simply be modulated as needed and relayed via generalization.

The centerpiece of this work is a “generalized reinforcement learning” (GRL) model that addresses specific aspects of generalization by efficiently exploiting correlational structure between both states and actions. Augmenting the model‐free scheme in this parsimonious way is another approach amid a zeitgeist inclined to more sophisticated alternatives to the basic RL framework such as proper model‐based control. Whereas this model still employs the temporal‐difference (TD) prediction method (Dayan, 1992; Dayan & Sejnowski, 1994; Sutton, 1988; Sutton & Barto, 1998), the algorithm is modified—not only updating the estimated value of an experienced state or action but also generalizing so as to flexibly transfer value information to other states and actions related to what was experienced. As opposed to separate counterfactual RPE signals, this singular generalized RPE signal functions as a heuristic for relaying the counterfactual information that can be derived directly from immediate experience. To implement the present optimization for both behavioral and neural modeling, we introduced the different types of generalization in parallel as enhancements of the “critic/Q‐learner” model (Colas et al., 2017) that we previously developed and validated as a bridge between the “actor/critic” model (Barto et al., 1983, 2021; Sutton, 1984; Witten, 1977) and the “Q‐learning” model (Watkins, 1989; Watkins & Dayan, 1992).

**FIGURE 1:**
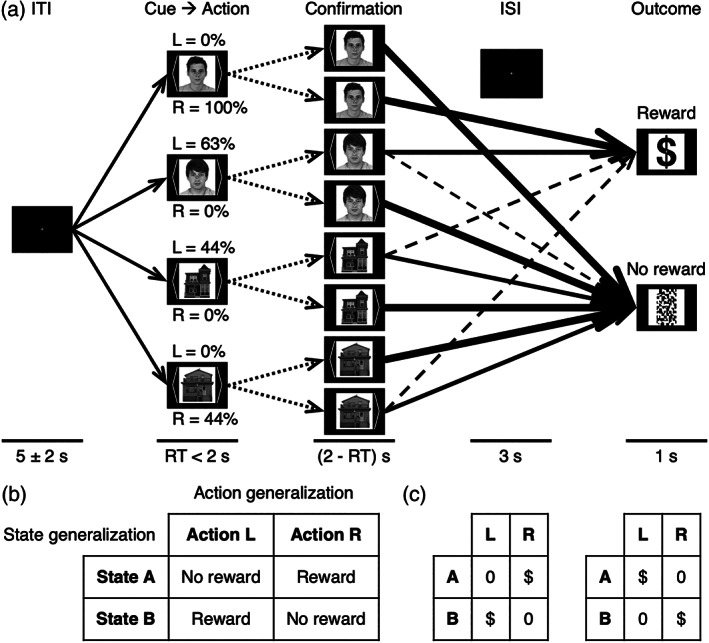
Task. (a) This schematic of the hierarchical reversal‐learning task performed during fMRI scanning includes the probabilities of a rewarded outcome in one of 12 blocks. Following an intertrial interval (ITI) with a fixation cross, one of four paired states (i.e., cues) was presented with equal probability, prompting the participant to choose either the left‐hand action (“L”) or the right‐hand action (“R”). Confirmation of the action at the reaction time (RT) was followed by an interstimulus interval (ISI) and finally an outcome of either a monetary reward or no reward as feedback. The paired state categories were faces and houses for the 3‐T version or colors and directions of motion for the 7‐T version. Dotted arrows symbolize the two possible actions. Solid arrows represent equally or more likely state transitions, whereas dashed arrows represent less likely transitions. Arrow thickness corresponds to the weight of an outcome's probability. (b) Only one action was rewarded per state, thereby facilitating discriminative action generalization. States were paired within a category as “state A" and “state B" such that opposite actions were rewarded between the two states, thereby facilitating discriminative state generalization. One of two possible arrangements for hierarchical reward structure (independent of probabilities) is shown here, corresponding to the face category for this example block: The upper face is “state A”, and the lower face is “state B”. There was no pairing between the independent categories. (c) The second possible arrangement is also shown for comparison. The two possibilities alternated within categories as this anticorrelational rule remained constant through reversals that remapped categories between blocks. For an optimal learner, this binary metastate determines the cognitive map or model of generalizable task structure, which for a proper model‐based algorithm is an explicit model but for generalized reinforcement learning is an implicit model.

Suitably for testing this GRL model, a hierarchical reversal‐learning paradigm (Figure 1) allowed for associative generalization but favored discriminative generalization across both states and actions. The tightly controlled task accomplished this with high‐order structure imposed to link available actions as well as subsumed states to discriminate between within stimulus categories. This embedded task structure provided participants with opportunities to recognize and exploit patterns across related events in separate trials so as to maximize reward. To facilitate action generalization, only one of two actions would be rewarded per state as a rule. Moreover, to facilitate state generalization, states (i.e., visually discriminable cues) were also paired within a category such that opposite actions were rewarded between the two states. The rule within each state category thus defined a hierarchical metastate with mapping that could reverse independently of that for the other category's binary metastate. The optimal strategy in this setting is inverse generalization that effectively infers and leverages anticorrelational interdependencies both between complementary actions within each state and between complementary states within each category.

**FIGURE 2:**
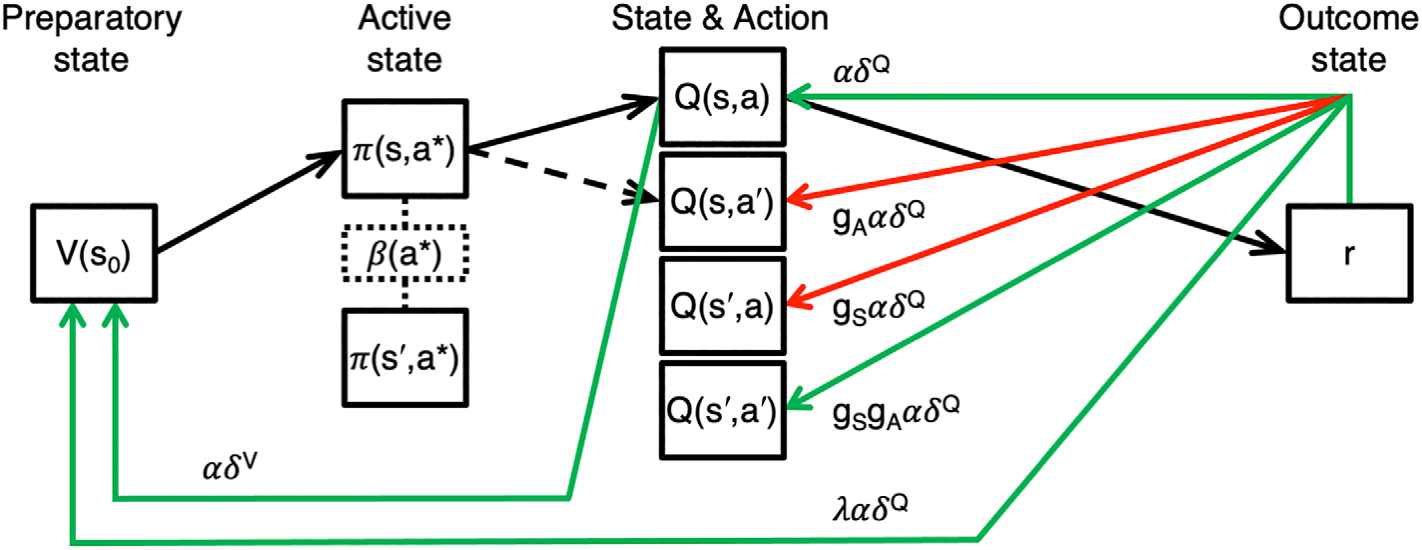
The “generalized reinforcement learning” (GRL) model. Compare to Figure 1. Here depicted in its 7‐parameter form, the GRL model introduces the concepts of state generalization (*g*_*S*_) and action generalization (*g*_*A*_) as enhancements of the “critic/Q‐learner” model, which is represented by the case where *g*_*A*_ = *g*_*S*_ = 0. The agent begins the trial in the preparatory state *s*_*0*_ having a state value *V(s*_*0*_) cached by the “critic” module. At trial onset, the agent is presented with a random active state *s* having a complementary state *s′* within the same category (e.g., faces). The agent's probabilistic action‐selection policy π(s,a*) over available actions a* is determined by not only their respective action values Q(s,a*), which are cached by the “Q‐learner” module, but also action‐specific bias and hysteresis β(a*). For this example, the agent's chosen action *a* corresponds to the greater action value *Q(s,a)* that updates *V*(*s*_*0*_ *)* via a state‐value‐prediction error *δ* ^*V*^ weighted by the learning rate *α*, which follows from this temporal‐difference (TD) algorithm that also tracks passive states. The outcome of the action is a reward *r* that updates *Q(s,a)* by way of an action‐value‐prediction error *δ* ^*Q*^. With the “TD(*λ*)” eligibility trace, *V(s*_*0*_ *)* is also updated a second time by this same reward‐prediction error (RPE) but reweighted with a decay multiplier as the eligibility parameter *λ*. With analogy to the temporal generalization of TD(*λ*), this new GRL model postulates that the generalized RPE signal is duplicated again, reweighted by *g*_*A*_, and relayed to the action value *Q(s,a′)* representing the complementary nonchosen action *a′* within state *s*. Likewise, the generalized RPE is reweighted by *g*_*S*_ and relayed to the action value *Q(s′,a)* representing the chosen action *a* for complementary state *s′*. Finally, these parameters interact as a combined weight *g*_*S*_ *g*_*A*_ modulating the RPE relayed to action value *Q(s′,a′)* for the complementary action *a′* within the complementary state *s′*. Positive value updates are indicated with green arrows for this rewarded example trial, whereas negative updates for the complementary state and the complementary action (but not their combination) are indicated with red arrows. The signs of these updates reflect discriminative generalization (−1 ≤ *g*_*A*_ < 0 and −1 ≤ *g*_*S*_ < 0), which is optimal here because of the anticorrelational structure across states and actions.

Yet the task is difficult in a probabilistic and changing environment producing noisy input, and fully recognizing interdependencies across trials becomes nontrivial as working memory is taxed. These cognitive demands may instead predispose an uncertain learner to implicit generalization. Although the quasi‐model‐based GRL model does not include an explicit representation of the linkage between states or actions, the optimality of discriminative generalization follows from the potential for the generalizing agent to incorporate more of the available information and update value representations four times as frequently in this case (Figure 2). As an anticipatory strategy, this reduces uncertainty in preparation for the next encounter within either a given state or its category.

There is considerable precedent for inquiry surrounding generalization and structure in learning (Bush & Mosteller, 1951b; Ghirlanda & Enquist, 2003; Harlow, 1949; Shepard, 1957, 1987; Tenenbaum & Griffiths, 2001; Tversky, 1977), and this is even the case for the specific domain of value‐based learning (Ballard et al., 2019; Baram et al., 2021; Behrens et al., 2018; Bernardi et al., 2020; Bromberg‐Martin et al., 2010; Daw & Shohamy, 2008; Doll et al., 2012; Doll, Duncan, et al., 2015; Doll, Shohamy, & Daw, 2015; Gerraty et al., 2014; Gershman & Niv, 2015; Hampton et al., 2006, 2007; Karagoz et al., 2022; Kool et al., 2016, 2017, 2018; Lehnert et al., 2020; Liu et al., 2021; Mattar & Daw, 2018; O'Doherty, 2012; Park et al., 2020; Prévost et al., 2013; Sadacca et al., 2016; Schulz et al., 2020; Watanabe & Hikosaka, 2005; Wimmer et al., 2012; Wimmer & Shohamy, 2012; Wunderlich et al., 2011). Yet typical approaches have emphasized strictly associative forms of generalization based on equivalence or similarity; in the present context, these are actually counterproductive as conflation—that is, overgeneralization. Uniquely for the present study, its explication extends to individual learners and how discriminative generalization can manifest (or not manifest) across representations of both states and actions. The GRL model is sensitive to not only discriminative generalization, which is presently optimal, but also associative overgeneralization or simple undergeneralization, thereby capturing possible variability in how humans might generalize with true or false beliefs or just fail to generalize altogether. With an aim for pragmatism, efficiency, and flexibility rather than pure optimality, these parameterized forms of associative or discriminative generalization and state or action generalization were framed to dovetail with classic RL and its operating constraints in the midst of stochasticity and parallel effects of action‐specific bias and hysteresis.

While serving to demonstrate the robustness of the techniques, this multisite study also allowed for a more diverse sample as part of the emphasis on individual differences (cf. Colas et al., 2017; Schönberg et al., 2007). Human participants performed one of two versions of the structured learning task while their brains were scanned with functional magnetic‐resonance imaging (fMRI). The first experiment was conducted at a now‐standard field strength (3 T) across five separate laboratories, whereas the second was conducted at a high (or “ultra‐high”) field strength (7 T) in parallel so as to elucidate subtle neural signatures of the GRL model with high fidelity, introducing commensurable state‐of‐the‐art imaging for a paradigm that lacks precedent for a high‐field or multifield fMRI study (cf. Beisteiner et al., 2011; Colizoli et al., 2021; Da Costa et al., 2015; de Hollander et al., 2017; Morris et al., 2019; Sengupta et al., 2018; Theysohn et al., 2013; Torrisi et al., 2018; Zaretskaya et al., 2020; but see Fontanesi, Gluth, Rieskamp, et al., 2019). While we did examine cortical signals, we primarily focused on subcortical regions of the basal ganglia that have been implicated in RL with evidence from earlier studies. Here the GRL model again benefits from the anchor of RL insofar as prior literature from the classic RL perspective still provides a firm foundation for further constraining hypotheses about signals in the brain. Bolstered by the advantages of high‐field fMRI (De Martino et al., 2018; Dumoulin et al., 2018; Torrisi et al., 2018; Uğurbil, 2018), our neuroimaging protocols were optimized for higher spatial resolution to pinpoint RL and GRL mechanisms in not only the striatum but also the dopaminergic midbrain. The technical challenges posed by measurements within elusive dopaminergic nuclei (Barry et al., 2013; de Hollander et al., 2015, 2017; Düzel et al., 2009, 2015) were addressed by adopting tailored measures for image preprocessing and denoising.

The first hypothesis was that the GRL model offers a superior account of motivated behavior and especially the distribution of performance at the level of individual participants. This quasi‐model‐based extension of model‐free RL could even stand to outcompete more unambiguously model‐based solutions, including delta learning with a state‐prediction error (SPE) (cf. Gläscher et al., 2010; Lee et al., 2014)—or here a “metastate‐prediction error” (MPE)—as well as more sophisticated Bayesian inference with a hidden Markov model (HMM) (Ghahramani, 2001; cf. Hampton et al., 2006; Prévost et al., 2013). Second, this model was hypothesized to successfully capture dynamics of neural activity (O'Doherty et al., 2007) associated with the computations characterizing RL as implemented within mesostriatal circuits (Chase et al., 2015; Colas et al., 2017; Garrison et al., 2013; O'Doherty et al., 2003; O'Doherty et al., 2004; Pauli et al., 2015; Schönberg et al., 2007). Third, predictions for value signals in both the ventral striatum and ventromedial prefrontal cortex (vmPFC) (Bartra et al., 2013; Behrens et al., 2008; Chase et al., 2015; Clithero & Rangel, 2014; Colas et al., 2017; Gläscher et al., 2009; Hare et al., 2008; Jocham et al., 2011; Kim et al., 2006) were also tested alongside RPE signals, which poses a challenge because these two types of signals as well as decision signals are all interconnected. Fourth, targeting predictions entirely specific to the GRL model, interaction effects in the basal ganglia as well as the hippocampus were expected to reflect the relaying of learning signals to representations of other states and actions; these interactions could be between RPE signaling and state generalization or between RPE signaling and action generalization. The hippocampal formation of the medial temporal lobe is a viable candidate for representing not only spatial topological maps (Moser et al., 2008; O'Keefe & Nadel, 1978) but also cognitive maps (Lewin, 1935, 1936; Tolman, 1948) such as in this more abstract space of states and actions (Ballard et al., 2019; Baram et al., 2021; Behrens et al., 2018; Bernardi et al., 2020; Cazé et al., 2018; Daw & Shohamy, 2008; Gerraty et al., 2014; Liu et al., 2019, 2021; Mattar & Daw, 2018; Momennejad et al., 2018; Park et al., 2020; Schuck & Niv, 2019; Wimmer et al., 2012; Wimmer & Shohamy, 2012).

The aims of the present study thus include first replicating and then building upon the established narrative of RL in the human brain, encompassing a trichotomy of value, decision, and learning signals. With a parsimoniously optimized implementation of the algorithmic template of RL, this flexible scheme for associative or discriminative generalization across states and actions broadens this narrative for predictably structured environments. Furthermore, with that narrative there arises an opportunity to reflect on how this generalization paradigm—as distinguished from one defined by a multistep task (Bellman, 1957; Daw et al., 2005, 2011; Gläscher et al., 2010; Lee et al., 2014; Sutton & Barto, 1998), for example—can relate to model‐free, model‐based, or quasi‐model‐based aspects of structural learning.

## 2 RESULTS

The first version of the structured learning task included fMRI at 3 T and faces or houses as stimuli (16 in total), whereas the second version included high‐resolution fMRI at 7 T and colors or directions of motion as stimuli (4 in total). These different versions were acquired in parallel, and the advantages of the differences in stimuli between them were twofold. The prosaic advantage applies to the 7‐T fMRI data, which are more susceptible to signal dropout: Owing to discrepancies between the magnetic properties of the cerebrum and the cerebellum and the properties of the interstitial space between them, there is a risk of dropout in the vicinity of the fusiform gyrus and (to a lesser extent) the parahippocampal gyrus—that is, the fusiform face area (FFA) (Kanwisher et al., 1997) and the parahippocampal place area (PPA) (Epstein & Kanwisher, 1998), which would relate to processing of face and house stimuli (i.e., states), respectively. The more substantial advantage is that replicating both behavioral and neural results between somewhat different experiments rather than strictly identical experiments can speak to the robustness or generality of a given effect.

### 2.1 Participant groups

Within each data set (i.e., the 3‐T Face/House (“3FH”) version or the 7‐T Color/Motion (“7CM”) version), the first step of the analysis entailed dividing participants into three subgroups according to model‐independent performance on the task (Schönberg et al., 2007) as well as the results of model fitting (Colas et al., 2017) (Table 1). Learning performance could thus be related to both behavioral and neural aspects of the modeling for this difficult task. A subset of participants was initially set aside as the “Good learner” (“G”) group (3FH: *n* = 31/47; 7CM: *n* = 16/22) if choice accuracy was significantly greater than the chance level of 50% for a given individual (*p* < .05). The remaining participants for whom the null hypothesis of chance accuracy could not be rejected at the individual level (*p* > .05) were further subdivided between the “Poor learner” (“P”) group (3FH: *n* = 9/47; 7CM: *n* = 5/22) and the “Nonlearner” (“N”) group (3FH: *n* = 7/47; 7CM: *n* = 1/22) according to whether or not an RL model could yield a significant improvement in goodness of fit relative to a nested hysteresis model without sensitivity to reward or its omission. Despite additional free parameters, the hysteresis model was justified statistically as a baseline model superior to the chance or intercept models (Tables S1‐S15).

**TABLE 1:** Participant groups

	3‐T Face/House			7‐T Color/Motion		
	Good learner	Poor learner	Nonlearner	Good learner	Poor learner	Nonlearner
*n*	31	9	7	16	5	1
Accuracy (%)	62.8 (5.4)	50.1 (3.1)	50.1 (3.0)	62.3 (5.6)	49.8 (4.9)	50.0
Reaction time (ms)	974 (129)	757 (107)	671 (104)	989 (87)	784 (163)	1043
Missed trials (%)	4.2 (6.8)	8.1 (10.5)	9.5 (9.8)	10.1 (9.1)	12.5 (12.4)	30.7
Age (y)	26.0 (4.9)	25.1 (5.0)	23.9 (5.2)	26.9 (4.2)	31.8 (9.8)	27
Male:Female (%)	54.8	55.6	71.4	43.8	100	0

*Note*: A subset of participants was initially set aside as the “Good learner” group if choice accuracy was significantly greater than chance at the individual level (*p* < .05). The remaining participants with chance accuracy (*p* > .05) were assigned to either the “Poor learner” group or the “Nonlearner” group according to whether or not a learning model could yield an improvement in fit relative to a hysteresis model without sensitivity to learnable outcomes. A speed‐accuracy tradeoff was exhibited between groups with concomitant effects in reaction time such that the Good‐learner group was the slowest to respond (*p* < .05). Standard deviations are listed in parentheses below corresponding means.

As part of the overarching computational framework—that is, not only RL per se but also the associated policy for action selection—reaction time (RT) was measured to implicitly relate dynamical models of decision making (Busemeyer & Townsend, 1993; Colas, 2017; Laming, 1968; Luce, 1986; Ratcliff, 1978; Usher & McClelland, 2001) to this context of active value‐based learning (Ballard & McClure, 2019; Fontanesi, Gluth, Spektor, et al., 2019; Fontanesi, Palminteri, & Lebreton, 2019; Frank et al., 2015; Luzardo et al., 2017; McDougle & Collins, 2021; Miletić et al., 2020, 2021; Millner et al., 2018; Pedersen et al., 2017; Pedersen & Frank, 2020; Ratcliff & Frank , 2012; Sewell et al., 2019; Sewell & Stallman, 2020; Shahar et al., 2019; Viejo et al., 2015). As more difficult decisions were hypothesized to be slower (see below), so too were decisions made more conscientiously by more attentive learners. Regarding the latter hypothesis, both data sets exhibited a speed‐accuracy tradeoff (Garrett, 1922; Johnson, 1939) between groups with effects in RT such that the Good‐learner group with high accuracy was also the slowest to respond (3FH‐GP: *M* = 217 ms, *t*_*38*_ = 4.60, *p* < 10^−4^; 3FH‐GN: *M* = 304 ms, *t*_*36*_ = 5.81, *p* < 10^−6^; 7CM‐GP: *M* = 205 ms, *t*_*19*_ = 3.72, *p* < 10^−3^). Likewise, the Poor‐learner group was marginally slower than the Nonlearner group (3FH‐PN: *M* = 87 ms, *t*_*14*_ = 1.63, *p* = .063).

### 2.2 Model comparison

The present GRL model has seven free parameters: two for basic RL (*α*, *τ*), three for action‐specific bias and hysteresis (*β*_*0*_, *λ*_*β*_, *β*_*R*_), and two for generalization (*g*_*A*_, *g*_*S*_) (see Section 4). This and 16 other models were formally compared with inclusion of a full factorial design permuting the novel factors of action generalization and state generalization while controlling for outcome‐independent effects of action‐specific bias and hysteresis (Colas et al., 2017). The candidates included basic model‐free RL (1 model), quasi‐model‐based GRL (10 models), the model‐based SPE (with delta learning) (1 model), the model‐based HMM (with Bayesian learning) (2 models), and dual‐systems models that are both model‐free and model‐based (3 models) (Table 2). (Note that the “state” determining the SPE or the HMM's hidden state is not the cue itself but rather the cue category's metastate for generalizable structure represented with two possibilities shown in Figure 1c.)

**TABLE 2:** Model parameters

			RL	GRL			SPE	HMM		Dual
Model	MF vs. MB	df	*α*	*g*_*A*_	*g*_*S*_	*g*_*SA*_	*α*_*SPE*_	*θ*_*0*_	*θ*_*1*_	*w*_*MB*_
A0|S0	MF	5	*α*	—	—	—	—	—	—	—
A−|S0	MF (~MB)	5	*α*	−1	—	—	—	—	—	—
AX|S0	MF (~MB)	6	*α*	*g*_*A*_	—	—	—	—	—	—
A0|S+	MF	5	*α*	—	+1	—	—	—	—	—
A0|S−	MF (~MB)	5	*α*	—	−1	—	—	—	—	—
A0|SY	MF (~MB)	6	*α*	—	*g*_*S*_	—	—	—	—	—
A−|S+	MF (~MB)	5	*α*	−1	+1	−1	—	—	—	—
A−|S−	MF (~MB)	5	*α*	−1	−1	−1	—	—	—	—
AW|SW	MF (~MB)	6	*α*	*g*	*g*	*g*	—	—	—	—
AX|SY	MF (~MB)	7	*α*	*g*_*A*_	*g*_*S*_	*g*_*A*_	—	—	—	—
AX|SY|Z	MF (~MB)	8	*α*	*g*_*A*_	*g*_*S*_	*g*_*SA*_	—	—	—	—
SPE	MB	5	—	—	—	—	*α*_*SPE*_	—	—	—
SPE+RL	MF + MB	7	*α*	—	—	—	*α*_*SPE*_	—	—	*w*_*MB*_
HMM0	MB	5	—	—	—	—	—	*θ*_*0*_	—	—
HMM	MB	6	—	—	—	—	—	*θ*_*0*_	*θ*_*1*_	—
HMM0+RL	MF + MB	7	*α*	—	—	—	—	*θ*_*0*_	—	*w*_*MB*_
HMM+RL	MF + MB	8	*α*	—	—	—	—	*θ*_*0*_	*θ*_*1*_	*w*_*MB*_

*Note*: All of the learning models are listed in ascending order of complexity both within and across classes: RL is the most simple and followed by GRL, the state‐prediction error (SPE) (i.e., metastate‐prediction error or MPE), the hidden Markov model (HMM), and lastly the most complex dual‐systems models. The algorithms are described in terms of being model‐free (“MF”), model‐based (“MB”), or quasi‐model‐based (“~MB”). Model‐free and (cognitive) model‐based learning rates are listed as *α* and *α*_*SPE*_ for the RPE and the SPE, respectively. For RL and GRL, the labels “A0”, “A−”, and “AX” denote the absence of action generalization (*g*_*A*_ = 0), maximally optimal discriminative action generalization (*g*_*A*_ = −1), and free action generalization (−1 ≤ *g*_*A*_ ≤ 0), respectively. The labels “S0”, “S+”, “S−”, and “SY” denote the absence of state generalization (*g*_*S*_ = 0), maximally suboptimal associative state generalization (*g*_*S*_ = 1), maximally optimal discriminative state generalization (*g*_*S*_ = −1), and free state generalization (−1 ≤ *g*_*S*_ ≤ 1), respectively. GRL model “AW|SW” is limited to a single free parameter *g* shared between these two types of generalization (*g*_*S*_ = *g*, *g*_*A*_ = min{0, *g*_*S*_}). GRL model “AX|SY|Z" adds a free parameter *g*_*SA*_ for an interaction term *g*_*S*_ *g*_*SA*_ (i.e., *g*_*SA*_ ≠ *g*_*A*_). The HMM0 variant shares a consistency parameter *θ*_*0*_ with the full HMM but omits the reversal rate (*θ*_*1*_ = 0). Dual‐systems models ("MF + MB") include a weighting parameter *w*_*MB*_ for the model‐based system. “df” stands for degrees of freedom.

In this context, a negative sign for the action‐generalization weight (−1 ≤ *g*_*A*_ < 0) represents correct recognition of the fixed complementarity between available actions, thereby speeding up learning. Likewise, a negative sign for the state‐generalization weight (−1 ≤ *g*_*S*_ < 0) represents correct recognition of the fixed complementarity between paired states rewarding opposite actions within a category, whereas a positive sign (0 < *g*_*S*_ ≤ 1) instead represents incorrect overgeneralization across states within a category as if they were identical. The extremes for these parameters (*g*_*A*_ = −1, *g*_*S*_ = 1, or *g*_*S*_ = −1), their absence (*g*_*A*_ = 0 or *g*_*S*_ = 0), and their equivalence (*g*_*A*_ = min{0, *g*_*S*_}) were combined as alternatives to determine whether the two additional degrees of freedom were justified. Moreover, whereas the 7‐parameter GRL model was expected to suffice with the assumption of an unparameterized interaction term *g*_*S*_ *g*_*A*_, a version with an additional free parameter *g*_*SA*_ for an interaction term *g*_*S*_ *g*_*SA*_ was also tested as part of due diligence.

**FIGURE 3:**
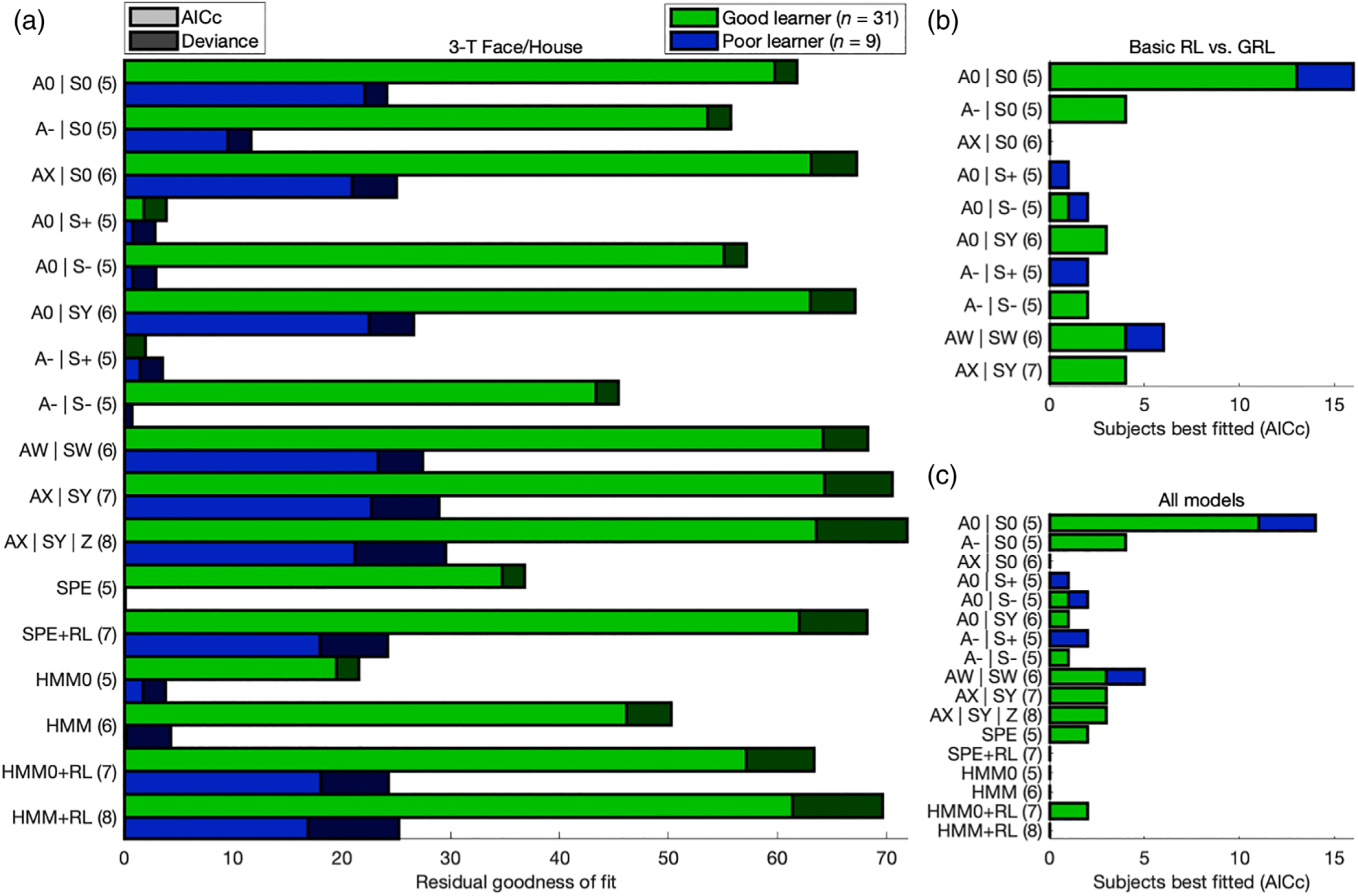
Model comparison: 3‐T Face/House version. (a) For each learning model, average goodness of fit relative to the outcome‐insensitive hysteresis model is shown with (light bars) and without (light and dark bars combined) a penalty for model complexity according to the corrected Akaike information criterion (AICc). The Good‐learner (green bars) and Poor‐learner (blue bars) groups are plotted separately. Emphasizing the result for the Good‐learner group, the 7‐parameter GRL model (“AX|SY”) outperformed all models even after correcting for model complexity and so justified inclusion of free parameters for both action and state generalization. The Nonlearner group is omitted here because these participants were best fitted by the hysteresis model following penalization. A more positive residual corresponds to a superior fit. Degrees of freedom are listed in parentheses. (b) Counts of the participants best fitted by the 7‐parameter GRL model and each of its nested models according to the AICc are plotted with separation between learner groups, demonstrating that the majority strongly generalize and exhibit heterogeneity in generalization strategies within both groups. These trends could only be captured by a fully parameterized two‐dimensional GRL model. (c) Broadening the scope to all models affirmed the preference for 7‐parameter GRL and suggested negligible utilization of proper model‐based strategies. This figure is related to Tables S1–S3.

**FIGURE 4:**
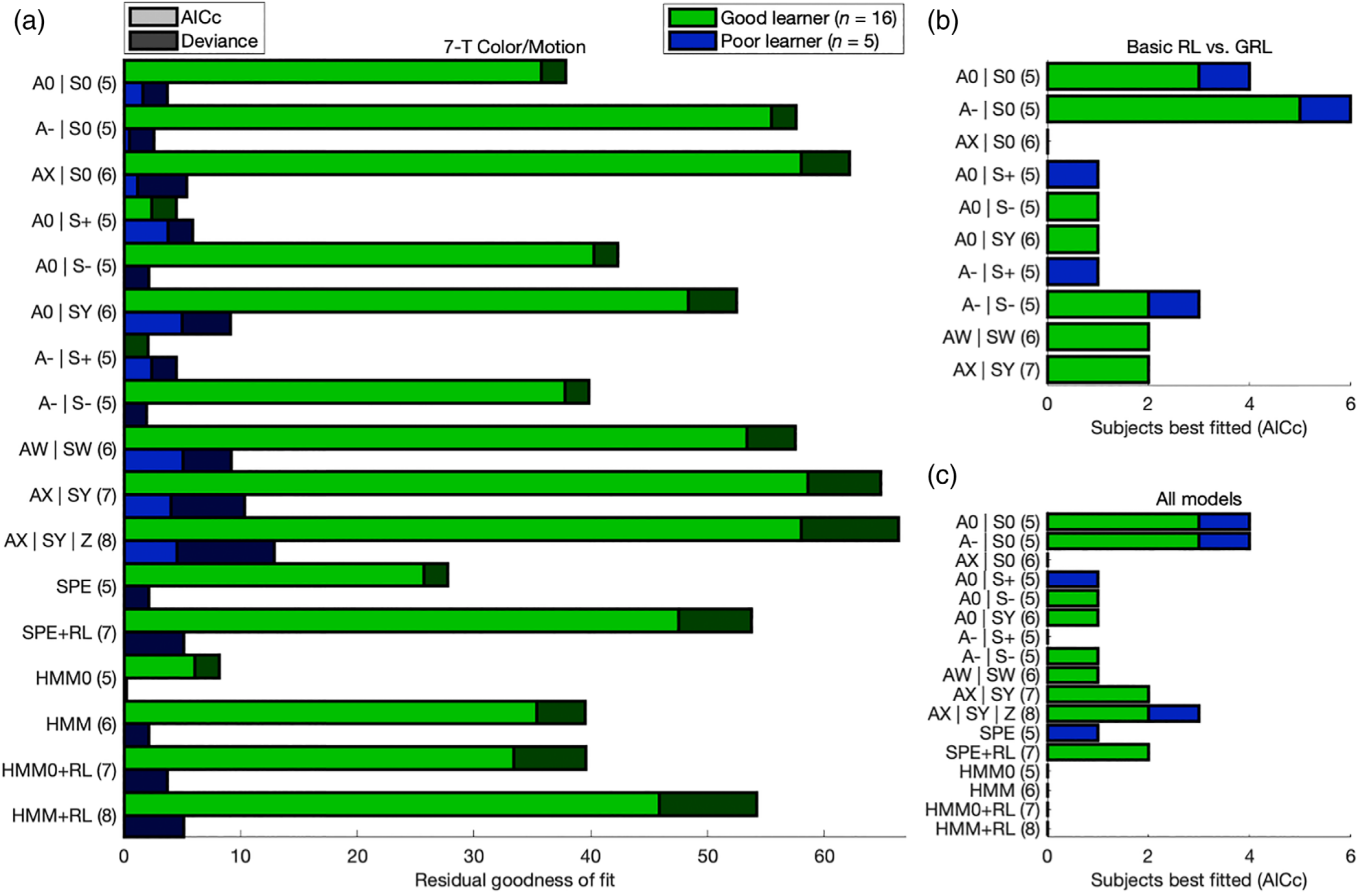
Model comparison: 7‐T Color/Motion version. Compare to Figure 3. Results were replicated in the 7‐T Color/Motion version of the experiment. This figure is related to Tables S4 and S5.

Across the Good learners of both data sets, the 7‐parameter GRL model including free parameters for both action and state generalization outperformed all nine models nested within it, the model that it was nested within, and six model‐based alternatives—even after correcting for model complexity according to the Akaike information criterion with correction for finite sample size (AICc) (Akaike, 1974; Hurvich & Tsai, 1989) (Figures 3a and 4a, Tables S1–S5). Crucially, when fitted at the level of individual subjects, this fully parameterized model could accommodate the heterogeneity in strategies for generalization observed within both Good‐learner and Poor‐learner groups—ranging from optimal (discriminative generalization) to semioptimal (undergeneralization) to suboptimal (associative overgeneralization) and with the majority strongly generalizing (3FH: *n* = 24/40; 7CM: *n* = 17/21) (Figures 3b/c and 4b/c). In other words, although a simpler alternative nested within the 7‐parameter model may provide a decent account for some individuals, this more complex model in itself provided the most parsimonious account for the greatest proportion of participants.

The lesser performance of the 8‐parameter GRL model argues against an explanation reduced to mere overfitting because this further increase in complexity did not significantly improve performance. In an attempt to reduce complexity, a shared parameter for state and action generalization (i.e., *g*_*A*_ = min{0, *g*_*S*_}) proved insufficient as these orthogonal factors actually required separate free parameters. Notably for the approximation of a proper model‐based strategy, the nested algorithm fixed with maximally optimal state and action generalization (i.e., *g*_*A*_ = *g*_*S*_ = −1) was not supported for most participants even when granted the benefits of fewer degrees of freedom. Likewise, the evidence did not favor the 5‐ or 6‐parameter model‐based algorithms (i.e., the SPE or the HMM) (cf. Aquino et al., 2020; Hampton et al., 2006; Prévost et al., 2013) or their respective dual‐systems counterparts.

To again affirm the discriminability of the 7‐parameter GRL model among both simpler and more complex alternatives, this entire pattern of results could be replicated after substituting simulated data generated by the fitted model itself (Figures S1 and S2, Tables S6–S10). Conversely, simulations generated with basic RL produced fitting results that instead aligned with basic RL (Figures S3 and S4, Tables S11–S15). That is, the complex model could be recovered from the complex model, and the simple model could be recovered from the simple model. This robust model discriminability rules out overfitting.

To complement the quantitative model comparison for overall goodness of fit, a posterior predictive check focused on a subset of diagnostic trials characterized by the purest effects of generalization. The hypothesis of generalized RL rather than basic RL could thus be tested at another level with qualitative falsification of the null hypothesis (Palminteri, Wyart, et al., 2017; Wilson & Collins, 2019). Based on parameter fits from the GRL model accommodating idiosyncratic generalization, Good and Poor learners were reclassified in “Discriminative generalizer” (*g*_*S*_ < 0) (3FH: *n* = 19/40; 7CM: *n* = 12/21), “Nongeneralizer” (*g*_*S*_ = 0) (3FH: *n* = 11/40; 7CM: *n* = 2/21), and “Associative generalizer” (*g*_*S*_ > 0) (3FH: *n* = 10/40; 7CM: *n* = 7/21) groups. The trials of interest corresponded to the first opportunities for generalization of reward within each block—that is, points in time before subsequent direct experience could update a value representation in the same direction as generalizable information would. After a given state‐action pair was rewarded for the first time, the crucial test was whether the complementary action would correctly be chosen upon the next encounter with the complementary state within the same category. Despite an absence of direct reinforcement for the second state's new reward contingencies—and even prior reinforcement to the contrary—the implicit inference of discriminative generalization nevertheless helps to boost this first‐generalization accuracy above chance following indirect generalizable reinforcement. Associative generalization counterproductively does the opposite in keeping with the false belief that the same action would be rewarded across the category.

**FIGURE 5:**
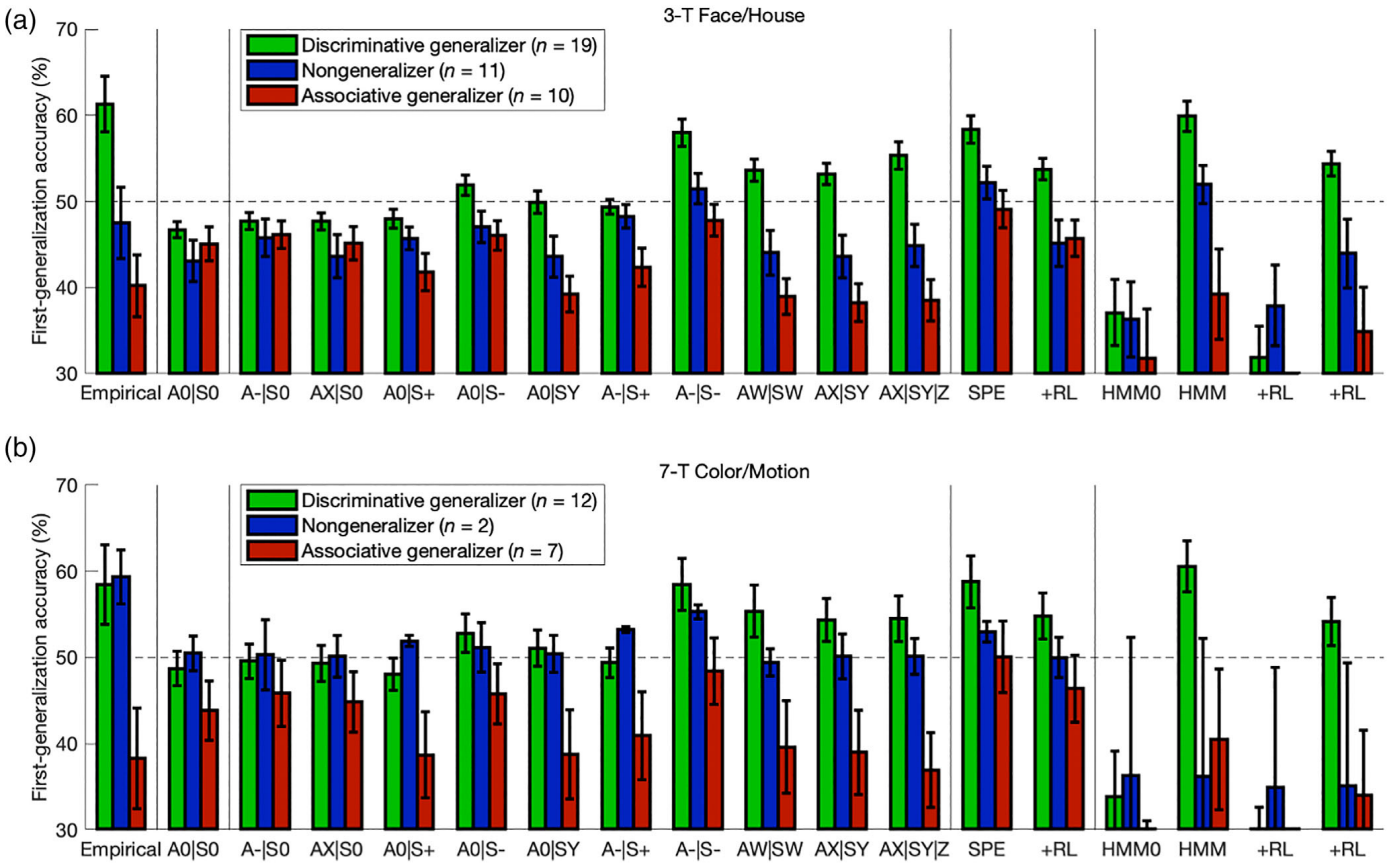
Posterior predictive check. (a) Focusing on only trials with the first opportunities for generalization of reward within each block, the purest effects of generalization were isolated in order to falsify basic RL. Using the GRL model, learners were reclassified in “Discriminative generalizer” (*g*_*S*_ < 0) (green bars), “Nongeneralizer” (*g*_*S*_ = 0) (blue bars), and “Associative generalizer” (*g*_*S*_ > 0) (red bars) groups. Only Discriminative generalizers could benefit from indirect reinforcement at these points in time, whereas Associative generalizers counterproductively overgeneralize the new information. In the absence of direct reinforcement for a complementary state's newly correct action, Discriminative generalizers, Nongeneralizers, and Associative generalizers performed above (*p* < .05), at (*p* > .05), and below (*p* < .05) chance at first generalization, respectively. Simulated data sets from each competing model were yoked to their respective empirical data sets. Whereas the simpler nested models fail to account for this interaction effect between groups (*p* > .05), two‐dimensional GRL models with state and action generalization capture the pattern successfully (*p* < .05). The (cognitive) model‐based alternatives (SPE and HMM) and their respective dual‐systems models (“+RL”) offered no substantial benefits here. (b) Results were replicated in the 7‐T Color/Motion version of the experiment. Error bars indicate standard errors of the means.

As expected across both data sets, Discriminative generalizers did in fact perform above chance with the first generalization (3FH: *M* = 11.3%, *t*_*18*_ = 3.51, *p* = 10^−3^; 7CM: *M* = 8.5%, *t*_*11*_ = 1.84, *p* = .047), whereas Associative generalizers performed not only below Discriminative generalizers (3FH: *M* = 21.1%, *t*_*27*_ = 4.09, *p* < 10^−3^; 7CM: *M* = 20.1%, *t*_*17*_ = 2.68, *p* = .008) but also below chance (3FH: *M* = 9.8%, *t*_*9*_ = 2.73, *p* = .012; 7CM: *M* = 11.7%, *t*_*6*_ = 2.00, *p* = .046) (Figure 5). Moreover, the Nongeneralizer group's first‐generalization accuracy was not significantly below chance (*p* > .05) but was below that of Discriminative generalizers (*M* = 13.8%, *t*_*28*_ = 2.61, *p* = .007). (Note that the subject counts of the generalization groups were not distributed as uniformly for the second sample, which left an insufficient Nongeneralizer group with a spuriously trending but nonsignificant result (*p* > .05) because of noise in the limited subset of trials that were separated from the majority in this analysis.) Across all learners, first‐generalization accuracy increased parametrically as the state‐generalization weight was more negative (3FH: *r* = 0.594, *t*_*38*_ = 4.55, *p* < 10^−4^; 7CM: *r* = 0.459, *t*_*19*_ = 2.25, *p* = .018). Altogether, hypotheses were confirmed across the board for these participant classifications derived from the GRL model.

Simulated data sets were generated with individually fitted instantiations of the computational models but yoked to their respective empirical data sets. That is, the simulated agents received input in silico according to what their respective participants actually encountered in the session. Regarding model comparison and falsification, this posterior predictive check confirmed that basic RL (sans generalization) was unable to account for the aforementioned effects, instead producing below‐chance first‐generalization accuracy across all groups of simulated agents (*p* < .05). Whereas the GRL agent has the capacity to infer this new category‐level information prior to direct experience, the basic RL agent is limited to only information experienced within the current state that is at best neutral but may even be the opposite of what should be inferred.

Although simpler nested models with fixed generalization roughly approximate the empirical pattern in first‐generalization accuracy, only the two‐dimensional GRL models with parameterization of both state and action generalization could achieve the qualitative interaction effects within and between generalization‐based groups (*p* < .05). (That the GRL model's fits to these trials are not quite perfect in terms of quantitative correspondence is merely a reflection of the fact that models were simultaneously fitted to the remaining 96% of trials along with the 4% emphasized at the moment; all of the trials were included in subsequent analyses that demonstrated the model's noteworthy quantitative precision.) Remarkably, despite classification here being based on state generalization, the coexistence of action generalization was also essential for calibrating the model's recapitulated effects: Another example of subpar performance for the “A0|SY” model with *g*_*A*_ = 0 was evident in first‐generalization accuracy remaining closer to chance among Discriminative generalizers (*p* > .05).

The more complex model‐based algorithms (SPE and HMM) at best qualitatively matched GRL here but did not always perform as well—even as half of a dyad including basic RL. (The reduced HMM0 variant in particular was limited by the rigidity of not explicitly representing reversals, such that new information that contradicts prior beliefs could not be integrated rapidly enough.) In this case, these alternatives did not offer any improvement that would justify sacrificing the parsimony of GRL. All things being equal, Occam's razor would bias model selection away from the computational complexity demanded by a more model‐based architecture as compared to a quasi‐model‐based but primarily model‐free architecture that boasts simplicity. Unlike the addition of free parameters, this lack of parsimony—including a less straightforward neural implementation—is not readily quantifiable for formal penalization in proportion to the concomitant increase in model complexity.

### 2.3 Behavioral modeling

With the model comparison pointing to the 7‐parameter GRL model, the next steps were to further verify and interpret the individually fitted parameters of this model with reference to learning performance (Table 3). The model could first quantify overall reward sensitivity with a logarithmic transformation of the ratio between the sum of all four generalized learning rates and the softmax temperature (cf. Colas et al., 2017; Schönberg et al., 2007). This sensitivity metric *log(α(1*−*g*_*A*_−*g*_*S*_ *+g*_*S*_ *g*_*A*_ *)/τ)* was greater for Good‐learner groups than for Poor‐learner groups across data sets (3FH: *M* = 1.873, *t*_*38*_ = 4.54, *p* < 10^−4^; 7CM: *M* = 1.510, *t*_*19*_ = 2.72, *p* = .007). Likewise, choice accuracy increased parametrically with sensitivity across both learner groups (3FH: *r* = 0.547, *t*_*38*_ = 4.03, *p* = 10^−4^; 7CM: *r* = 0.645, *t*_*19*_ = 3.68, *p* < 10^−3^). In keeping with the speed‐accuracy tradeoff, RT was analogously slower as sensitivity increased (with marginal significance for the latter data set) (3FH: *r* = 0.506, *t*_*38*_ = 3.62, *p* < 10^−3^; 7CM: *r* = 0.346, *t*_*19*_ = 1.61, *p* = .062).

**TABLE 3:** Parameters of the GRL model

	3‐T Face/House			7‐T Color/Motion		
	Good learner	Poor learner	Nonlearner	Good learner	Poor learner	Nonlearner
*n*	31	9	7	16	5	1
Accuracy (%)	62.8 (5.4)	50.1 (3.1)	50.1 (3.0)	62.3 (5.6)	49.8 (4.9)	50.0
Reward sensitivity *log(α(1*−*g*_*A*_−*g*_*S*_ *+g*_*S*_ *g*_*A*_ *)/τ)*	0.058 (0.280)	−1.815 (2.309)	−1.823 (2.009)	0.069 (0.337)	−1.442 (2.266)	−5.764
Learning rate *α*	0.517 (0.242)	0.269 (0.339)	0.483 (0.345)	0.555 (0.345)	0.540 (0.353)	0.372
Action generalization *g*_*A*_	−0.355 (0.367)	−0.321 (0.376)	−0.787 (0.357)	−0.535 (0.393)	−0.551 (0.482)	−1.000
Discriminative : None	21 : 10	6 : 3	7 : 0	13 : 3	4 : 1	1 : 0
State generalization *g*_*S*_	−0.184 (0.344)	0.367 (0.535)	0.359 (0.887)	−0.239 (0.390)	0.257 (0.819)	1.000
Disc. : None : Associative	18 : 9 : 4	1 : 2 : 6	2 : 0 : 5	11 : 1 : 4	1 : 1 : 3	0 : 0 : 1
Softmax temperature *τ*	0.698 (0.464)	0.737 (0.565)	3.066 (0.724)	0.700 (0.343)	1.298 (0.782)	2.157
Perseveration bias: Initial magnitude *β*_*0*_	−0.066 (0.235)	−0.133 (0.438)	−0.169 (1.034)	−0.130 (0.153)	−0.393 (0.949)	−1.278
Alternation : Perseveration	21 : 10	4 : 5	4 : 3	13 : 3	3 : 2	1 : 0
Perseveration bias: Inverse decay rate *λ*_*β*_	0.543 (0.371)	0.578 (0.404)	0.456 (0.421)	0.659 (0.318)	0.485 (0.403)	0.000
Rightward bias *β*_*R*_	0.113 (0.354)	0.160 (0.185)	0.391 (0.855)	0.167 (0.240)	0.245 (0.360)	−0.435
Leftward : Rightward	12 : 19	2 : 7	2 : 5	2 : 14	1 : 4	1 : 0
Intercept model: Residual deviance *D*_6_	78.56	48.67	16.75	73.97	42.15	22.28
Hysteresis model: Residual deviance *D*_3_	70.55	28.94	1.46	64.82	10.31	1.51

*Note*: Average fitted parameters for the preferred GRL model are listed for each participant group within each data set. Overall reward sensitivity was encapsulated by the ratio between generalized learning rates and temperature, which was greater for the Good‐learner group than for the Poor‐learner group in both data sets as expected (*p* < .05). Whereas action generalization did not differ between groups in either data set (*p* > .05), state generalization was more negative—that is, more optimal—for Good learners than for Poor learners (*p* < .05). The signs of individual fits are summarized as “discriminative” (−1 ≤ *g*_*A*_ < 0) or “none” (*g*_*A*_ = 0) for action generalization; “discriminative” (−1 ≤ *g*_*S*_ < 0), “none” (*g*_*S*_ = 0), or “associative” (0 < *g*_*S*_ ≤ 1) for state generalization; “alternation” (*β*_*0*_ < 0) or “perseveration” (*β*_*0*_ > 0) for hysteretic biases; and “leftward” or (*β*_*R*_ < 0) “rightward” (*β*_*R*_ > 0) for lateral biases. The residual deviance *D*_*df*_ (with degrees of freedom in the subscript) corresponds to the GRL model's improvement in fit relative to either null model. Standard deviations are listed in parentheses below corresponding means.

State generalization *g*_*S*_ was more negative—that is, more optimal—for Good learners than for Poor learners across data sets (3FH: *M* = 0.551, *t*_*38*_ = 3.71, *p* < 10^−3^; 7CM: *M* = 0.496, *t*_*19*_ = 1.89, *p* = .037). Likewise, across all learners, choice accuracy increased as state generalization was more negative (3FH: *r* = 0.509, *t*_*38*_ = 3.65, *p* < 10^−3^; 7CM: *r* = 0.571, *t*_*19*_ = 3.04, *p* = .003). Action generalization *g*_*A*_ did not differ between groups in either data set (*p* > .05). In other words, the Poor learners were primarily limited by difficulties with properly discriminating and generalizing between states within a category rather than actions within a state—the former being the more complex process here. The dissociation between state generalization and action generalization was confirmed by the complete absence of any correlation between these parameters across all learners (3FH: *r* = 0.006, *p* > .05; 7CM: *r* = −0.033, *p* > .05).

**FIGURE 6:**
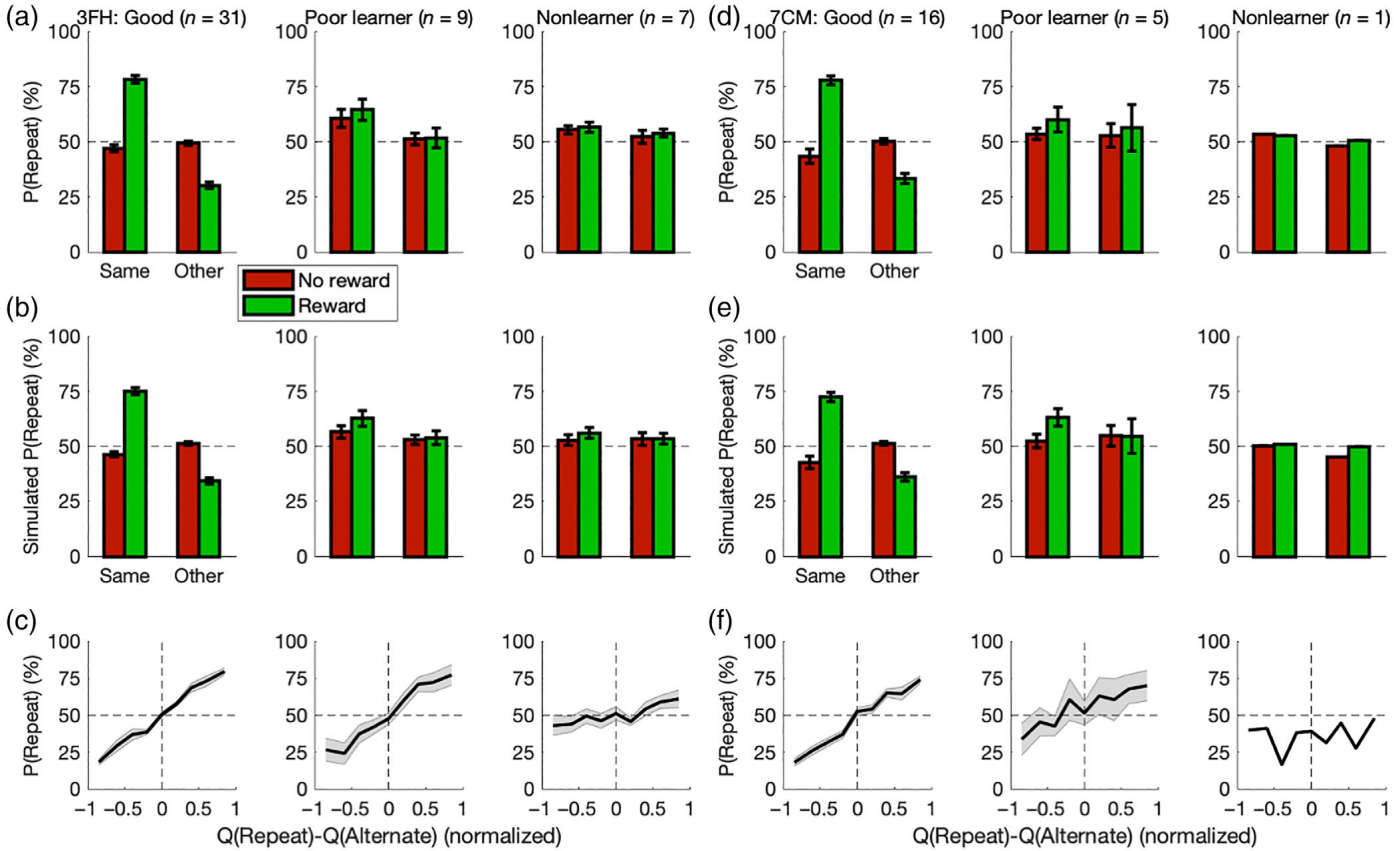
Behavioral modeling with the GRL model. (a) State generalization in particular relates to a qualitative pattern in choice behavior for this paradigm. Across all trials, conditions were defined by the most recent trials in which either the same (i.e., current) state was encountered or the other state within the current category was encountered; these trials were further binned according to whether the trial rewarded a given action (green bars) or provided no reward (red bars). While a rewarded action was more likely to be repeated for the same state among Good learners (*p* < .05), instead a nonrewarded action was more likely to be repeated from the other state (*p* < .05). This interaction effect (*p* < .05) follows from the complementarity of states within a category. Poor learners and Nonlearners did not exhibit such a pattern in behavior (*p* > .05). (b) To verify that the GRL model could reproduce these results with quantitative precision, simulated data sets were analyzed in the same fashion. (c) For all participant groups—including even “Nonlearners”—the probability of repeating the most recent action (independent of state) increased as a function of the difference between action values *Q*_*t*_ *(s*_*t*_ *,a)* derived from the GRL model (*p* < .05). (d–f) Results were replicated in the 7‐T Color/Motion version of the experiment. Error bars indicate standard errors of the means.

These forms of discriminative generalization speed up learning across trials, and as alluded to previously, state generalization here relates to a qualitative pattern in choice behavior based on an interaction effect of reinforcement between hierarchically paired states (Figure 6a/d). Whereas the previous analysis was concerned with isolating generalization effects in a subset of trials, this analysis across all trials addressed a mixture of effects such as pure RL, generalized RL, action‐specific biases, and stochasticity from noise and exploration. Conditions for preceding outcomes were defined by the most recent trials in which either the same (i.e., current) state was encountered or the other, complementary state within the current category was encountered; these trials were further binned according to whether the trial rewarded a given action or provided no such reward. Among Good learners of either data set, a rewarded action was more likely to be repeated within the same state (3FH: *M* = 31.3%, *t*_*30*_ = 13.52, *p* = 10^−14^; 7CM: *M* = 34.4%, *t*_*15*_ = 8.34, *p* < 10^−6^); in contrast, a nonrewarded action was more likely to be repeated after being performed in the other state (3FH: *M* = 19.2%, *t*_*30*_ = 15.95, *p* < 10^−15^; 7CM: *M* = 16.9%, *t*_*15*_ = 9.08, *p* < 10^−7^), producing an interaction effect (3FH: *M* = 50.5%, *t*_*30*_ = 16.47, *p* < 10^−16^; 7CM: *M* = 51.3%, *t*_*15*_ = 9.46, *p* < 10^−7^). The Poor‐learner and Nonlearner groups did not exhibit this pattern in their choices (*p* > .05). Identical analyses of simulations in a second posterior predictive check—the first being qualitative—confirmed that the GRL model could reproduce these results with quantitative precision (3FH‐G: *p* < .05; 3FH‐P: *p* > .05; 3FH‐N: *p* > .05; 7CM‐G: *p* < .05; 7CM‐P: *p* > .05) (Figure 6b/e). By incorporating action‐specific bias and hysteresis (Colas et al., 2017; Lau & Glimcher, 2005; Schönberg et al., 2007), this extended model simultaneously matched reward‐independent effects on the dynamic base rates of action repetition or alternation as well.

Additional validation of model fitting could be found in (computational) model‐based psychometric functions of choices and RTs; the former maps onto the standard softmax function embedded within the present model (Luce, 1959; Shepard, 1957; Sutton & Barto, 1998), and the latter has been shown to be generally applicable to RL (Ballard & McClure, 2019; Fontanesi, Gluth, Spektor, et al., 2019; Fontanesi, Palminteri, & Lebreton, 2019; Frank et al., 2015; Luzardo et al., 2017; McDougle & Collins, 2021; Miletić et al., 2020, 2021; Millner et al., 2018; Pedersen et al., 2017; Pedersen & Frank, 2020; Ratcliff & Frank, 2012; Sewell et al., 2019; Sewell & Stallman, 2020; Shahar et al., 2019; Viejo et al., 2015). (Given the two‐alternative forced choice, this logistic softmax model is nested within not only signal‐detection theory (Green & Swets, 1966) but also the drift‐diffusion model encompassing RT (Laming, 1968; Ratcliff, 1978; Ratcliff et al., 2016; Stone, 1960).) For all five participant groups across data sets—including even “Nonlearners” who actually do exhibit subtle signatures of learning—the probability of repeating the most recent action (independent of state) increased as a function of the difference between action values *Q*_*t*_ *(s*_*t*_ *,a)* derived from the GRL model (3FH‐G: *β* = 1.902, *t*_*30*_ = 9.66, *p* < 10^−10^; 3FH‐P: *β* = 1.986, *t*_*8*_ = 2.70, *p* = .014; 3FH‐N: *β* = 0.332, *t*_*6*_ = 4.53, *p* = .002; 7CM‐G: *β* = 1.668, *t*_*15*_ = 7.44, *p* = 10^−6^; 7CM‐P: *β* = 1.034, *t*_*4*_ = 2.50, *p* = .033) (Figure 6c/f). Along with effects on choices, RT became faster as the absolute difference between action values increased for 4 out of 5 participant groups (3FH‐G: *β* = 62 ms, *t*_*30*_ = 2.78, *p* = .005; 3FH‐P: *β* = 120 ms, *t*_*8*_ = 3.01, *p* = .008; 3FH‐N: *β* = 107 ms, *t*_*6*_ = 3.07, *p* = .011; 7CM‐G: *p* > .05; 7CM‐P: *β* = 58 ms, *t*_*4*_ = 2.28, *p* = .042). (The RT results for the 7‐T Color/Motion version—one of which is the null result—are given less weight in consideration of the dynamic stimuli that require more time to recognize via perceptual decision making.)

### 2.4 Neural substrates of the RL framework

**FIGURE 7:**
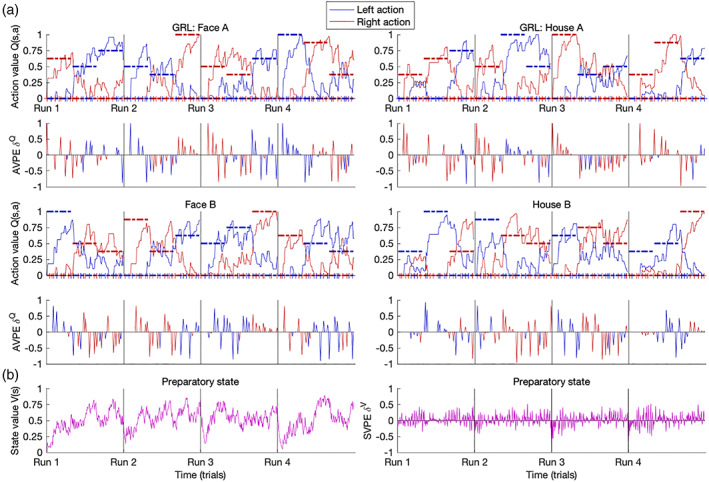
Predictions of the GRL model. Representative dynamics of value signals and learning signals generated by the GRL model are shown for the final participant in the Good‐learner group of the 3‐T Face/House data set. This modeling provided explicit quantitative predictions for internal decision variables within the (computational) model‐based fMRI analysis. Parameters were assigned as follows for this participant: *α* = 0.318, *g*_*A*_ = −0.710, *g*_*S*_ = −0.808, *λ* = 0.500, *τ* = 0.408, *β*_*0*_ = −0.067, *λ*_*β*_ = 0.753, and *β*_*R*_ = 0.178. (a) Tracking the probability of reward for the left and right actions (blue and red lines, respectively) in each of four active states, the model's estimates of action values *Q*_*t*_ *(s,a)* (solid lines) are plotted along with actual values (dashed lines) over the course of 12 blocks. Plotted below these value signals are time courses of the corresponding action‐value‐prediction error (AVPE) *δ* ^*Q*^_*t*_ signals. Discriminative state and action generalization are evident with counterfactual updates of values for the three nonexperienced state‐action pairs within a category (Figure S5). These additional updates occur despite only one state‐action pair being experienced with feedback. Each colored tick mark denotes an occurrence of the respective action. (b) Whereas active states were tracked by the Q‐learning component of this “critic/Q‐learner” (CQ) model, the preparatory state preceding each active state was tracked by the CQ model's critic module for passive states. Essentially tracking the probability of reward for the entire task, the model's estimates of state values *V*_*t*_ *(s*_*0*_ *)* are plotted alongside state‐value‐prediction error (SVPE) *δ* ^*V*^_*t*_ signals. From the temporal generalization of TD(*λ*), the value of the preparatory state was updated not only at the beginning of the trial but also at the end by way of the AVPE signal's eligibility trace.

Having demonstrated the efficacy of the GRL model and its fitted parameters with respect to behavior, a (computational) model‐based analysis followed suit for the neuroimaging data (O'Doherty et al., 2007). For each participant and their experienced sequence of events, this modeling generated explicit quantitative predictions for internal decision variables (Figures 7 and S5). The tripartite neural model was characterized by (1) learning signals as the generalized RPEs from the GRL model, (2) value signals from the GRL model, and (3) decision‐making signals as approximated by RT. Along with the hippocampus, the hypothesis space was constrained by focal regions of interest (ROIs) based on established precedents for the precise neural correlates of the RPE (Colas et al., 2017), subjective value (Bartra et al., 2013; Clithero & Rangel, 2014), and RT (Yarkoni et al., 2009). To further assess these neurophysiological signals in relation to learning performance evident in behavior, the participant groups were analyzed both collectively and separately for juxtaposition.

Reaction time served as a model‐independent proxy for neural decision‐making signals (Cisek, 2012; Cisek & Kalaska, 2010; Gold & Shadlen, 2007) that, with integration of sequential sampling, are characteristically ramping, bounded, and nonlinear (Colas, 2017; Usher & McClelland, 2001; Wang, 2002; Wong & Wang, 2006). Admittedly, limited temporal resolution translates to a risk of false positives at the level of interpretation when attempting to isolate decision signals among myriad other signals in the brain. Yet, although a measure of “time on task” is potentially relatable to constructs such as attention, arousal, difficulty, effort, engagement, or control, a longer RT essentially corresponds to greater cumulative neural activity for a dynamical decision‐making process that is integrated across time (Carp et al., 2010; Colas, 2017; Grinband et al., 2011; Hare et al., 2011; Shenhav et al., 2014; Weissman & Carp, 2013; Yarkoni et al., 2009). Trial‐by‐trial RT is a more direct proxy for decision signals than a model‐derived metric for normative difficulty such as the value difference—whether represented as the absolute difference *|Q(s,a*_1_ *)*−*Q(s,a*_2_ *)|* (unsigned) or as chosen value minus nonchosen value *Q(s,a)*−*Q(s,a′)* (signed) (Colas, 2017).

**FIGURE 8:**
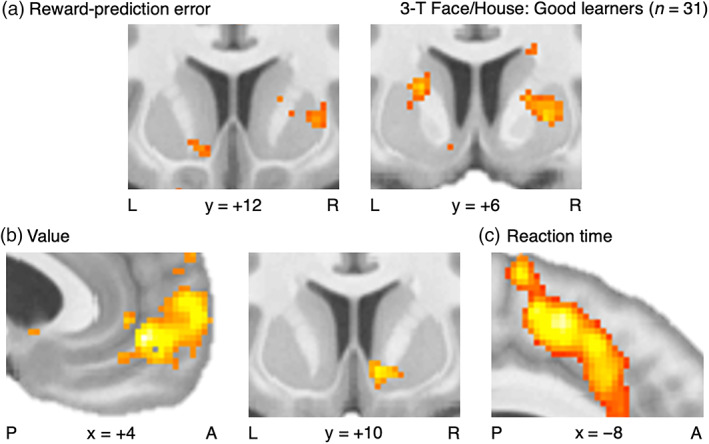
Neural substrates of the RL framework: 3‐T Face/House version. (a) At 3 T, reward‐prediction error (RPE) signals from the GRL model were significant at the set level (SVC *p*_*FWE*_ < 0.05) and identified throughout the striatum (*p* < .005), including the nucleus accumbens, the dorsal caudate nucleus, and the dorsal putamen (SVC *p*_*FWE*_ < 0.05). (b) Value signals from the GRL model were also significant at the set level (SVC *p*_*FWE*_ < 0.05) and identified in ventromedial prefrontal cortex (vmPFC) and the nucleus accumbens (*p* < .005, SVC *p*_*FWE*_ < 0.05). (c) As a proxy for decision‐making signals, reaction time (RT) was associated with greater activity in medial frontal cortex (MFC) (*p* < .005, SVC *p*_*FWE*_ < 0.05). “L”, “R”, “P”, and “A” orient the left, right, posterior, and anterior directions, respectively. This figure is related to Tables S16 and S18.

For the 3‐T Face/House images to first validate and expand the framework that the GRL model builds upon, analyses of the three key signals focused on the Good‐learner group in consideration of their more robust task‐relevant neural activity (Colas et al., 2017; Schönberg et al., 2007) (Figure 8, Tables S16 and S18; see S18 for summary). That is, learning signals in the brain are clearest among those who consistently learn well as reflected in their behavior. Sets of ROIs were specified a priori for mesostriatal RPE signals (7 ROIs) and corticostriatal value signals (4 ROIs). The networks identified as encoding RPE or value signals were both significant at the set level for these ROIs (SVC *p*_*FWE*_ < .05). RPE signals from the GRL model were identified throughout the striatum (*p* < .005), including the nucleus accumbens, the dorsal caudate nucleus, and the dorsal putamen (SVC *p*_*FWE*_ < .05) (Figure 8a). Regarding the dopaminergic midbrain, RPE signals were also observed in the substantia nigra (SN) (*p* < .005). Value signals from the GRL model were identified in vmPFC, the nucleus accumbens, and posterior cingulate cortex (PCC) (*p* < .005, SVC *p*_*FWE*_ < 0.05) (Figure 8b). In keeping with the decoupling of RPE and value signals in this paradigm, there were no common clusters in the striatum when testing for intersection of RPE and value networks (*p* > .005). Moreover, reaction time was associated with greater activity in medial frontal cortex (MFC) (*p* < .005, SVC *p*_*FWE*_ < 0.05) (Figure 8c).

**FIGURE 9:**
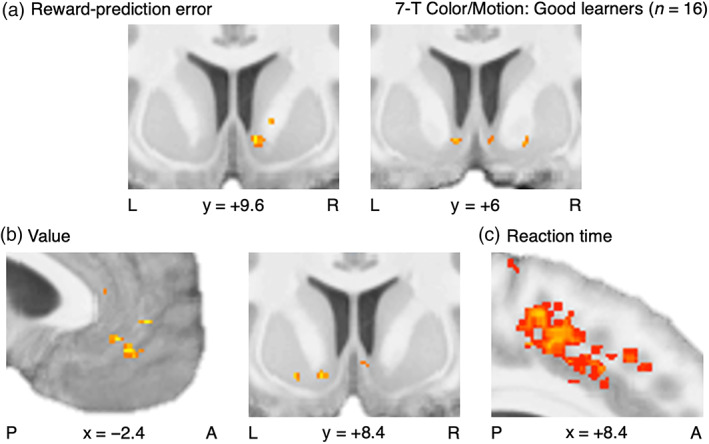
Neural substrates of the RL framework: 7‐T Color/Motion version. (a) At 7 T, RPE signals from the GRL model were again significant at the set level (SVC *p*_*FWE*_ < 0.05) and identified throughout the striatum (*p* < .005), including the nucleus accumbens (SVC *p*_*FWE*_ < 0.05). (b) Value signals from the GRL model were again significant at the set level (SVC *p*_*FWE*_ < 0.05) and identified in vmPFC and the nucleus accumbens (*p* < .005, SVC *p*_*FWE*_ < 0.05). (c) RT was again associated with greater activity in MFC (*p* < .005, SVC *p*_*FWE*_ < 0.05). “L”, “R”, “P”, and “A” orient the left, right, posterior, and anterior directions, respectively. This figure is related to Figure S6 and Tables S17 and S18.

The next portion of the fMRI analysis boasted greater spatial precision with high‐resolution imaging for the 7‐T Color/Motion data (Figures 9 and S6, Tables S17 and S18). The networks identified as encoding RPE or value signals were again both significant at the set level (SVC *p*_*FWE*_ < 0.05). RPE signals from the GRL model were localized within the SN and throughout the striatum (*p* < .005), including the nucleus accumbens (SVC *p*_*FWE*_ < 0.05) (Figures 9a and S6). Value signals from the GRL model were likewise identified in vmPFC and the nucleus accumbens (*p* < .005, SVC *p*_*FWE*_ < 0.05) (Figure 9b). Striatal RPE and value signals did not overlap here either (*p* > .005). For yet another replication, RT was again associated with greater activity in MFC (*p* < .005, SVC *p*_*FWE*_ < 0.05) (Figure 9c).

### 2.5 Neural substrates of the GRL model

Having elaborated on the RL framework within this paradigm, the second half of the neuroimaging analyses aimed to test additional predictions specific to the GRL model and as such entirely beyond the scope of basic RL. More precisely, interactions were tested for between RPE signals and either state generalization (i.e., −*g*_S_ */τ*) or action generalization (i.e., −*g*_A_ */τ*); such effects would be concordant with the hypothesis that there are relayed RPE signals mediating generalized updates of value representations that ultimately must interface with representations of states and actions such as in visual cortex and motor cortex, respectively (Lim et al., 2013; Magrabi et al., 2021; Philiastides et al., 2010). The topology of these representations and the relations between them is hypothesized to be encoded by a cognitive map maintained in the hippocampus, which would reflect downstream effects of generalized RPE signals from mesostriatal circuits without necessarily computing the RPE per se (cf. Ballard et al., 2019; Baram et al., 2021; Wimmer et al., 2012). These interaction effects were modeled with GRL parameters fitted at the level of individual subjects, including the temperature *τ* to factor in overall noise that diminishes the precision of the point estimates generated with the model's dynamics. Notably, the parameter for state generalization suggested greater emphasis given its greater intersubject variability and a more direct link to successful learning, but the less variable factor of action generalization was also investigated.

The fundamental conceptual dissociation between states and actions (Averbeck & O'Doherty, 2022; Colas et al., 2017; O'Doherty et al., 2004) suggested an a‐priori hypothesis that some mesostriatal and hippocampal circuitry would be uniquely implicated in either form of generalization. Accordingly, different categories of stimuli and different actions with different effectors evoked distinct neural representations that were amenable to fMRI by design—for example, engaging the FFA or the PPA with faces or houses, respectively. With implications for separable circuits for generalization, state and action representations were thus robust, specific, and discretized.

**FIGURE 10:**
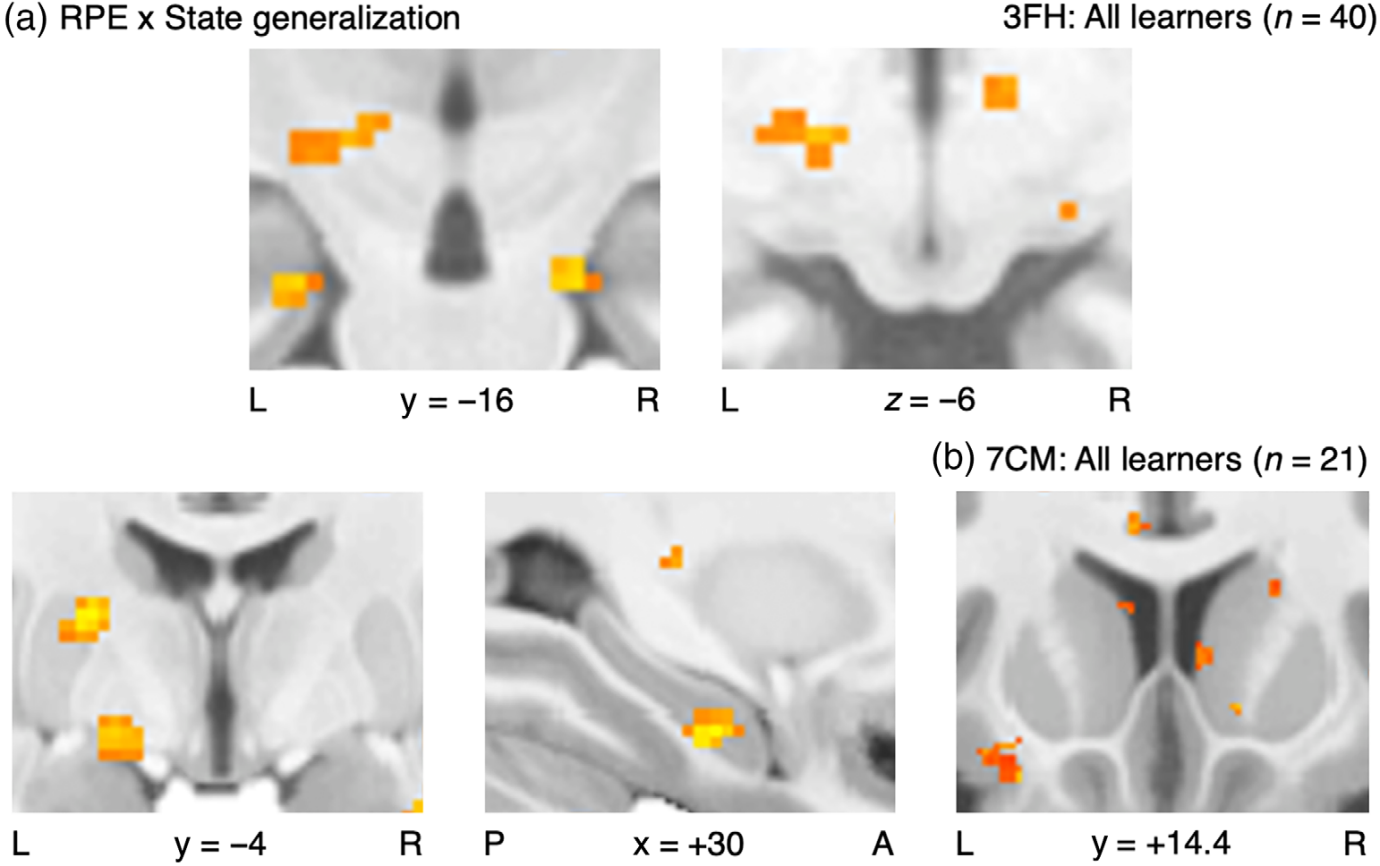
Neural substrates of the GRL model. (a) At 3 T, interaction effects between RPE signals and state generalization were significant at the set level (SVC *p*_*FWE*_ < 0.05) and identified in both the substantia nigra and the striatum (*p* < .005), including the posterior putamen in the vicinity of the dorsal caudate nucleus (SVC *p*_*FWE*_ < 0.05). In addition to mesostriatal circuits, generalization effects also modulated activity in the hippocampus (*p* < .005, SVC *p*_*FWE*_ < 0.05). (b) At 7 T, effects of state generalization were marginally significant at the set level (SVC *p*_*FWE*_ < 0.10) and identified throughout the striatum (*p* < .005), including the nucleus accumbens (SVC *p*_*FWE*_ < 0.05) and (with marginal significance) the anterior caudate nucleus (SVC *p*_*FWE*_ < 0.10). “L”, “R”, “P”, and “A” orient the left, right, posterior, and anterior directions, respectively. This figure is related to Figure S7 and Tables S19–S22.

First with the 3‐T Face/House images, this investigation of the GRL model warranted a wider sample of all learners for the sake of incorporating variability in generalization strategies or lack thereof (Figure 10a, Tables S19, S21, and S22; see S21 and S22 for summary). Crucially, those participants who did learn well were not necessarily taking advantage of the opportunities to generalize. With regard to the primary factor of state generalization, the network implicated in the interaction effect between RPE signals and the strength of generalization was significant at the set level for the same mesostriatal ROIs from the earlier RPE analysis (SVC *p*_*FWE*_ < 0.05). These state‐generalization interactions were aligned with the focal coordinate‐based ROI in the SN and also found in the striatum (*p* < .005), including the posterior putamen in the vicinity of the dorsal caudate nucleus (SVC *p*_*FWE*_ < 0.05) (cf. Doll, Duncan, et al., 2015; Horga et al., 2015; Lee et al., 2014; O'Doherty et al., 2003; Tricomi et al., 2009; Wunderlich et al., 2012) (Figure 10a). These generalization effects applied to the hippocampus as well (*p* < .005, SVC *p*_*FWE*_ < 0.05), confirming our hypothesis that this region is involved in relaying generalized learning signals to linked value representations.

For the 7‐T Color/Motion version (Figures 10b and S7, Tables S20–S22), effects of state generalization were most robustly identified in the nucleus accumbens (*p* < .005, SVC *p*_*FWE*_ < 0.05) (Figure 10b). Although the hippocampal result for state generalization did not extend across all learners in this case (*p* > .005), signals in the hippocampus among Good learners did yield generalization effects (*p* < .005, SVC *p*_*FWE*_ < 0.05). The results for generalization additionally comprised uncorrected effects elsewhere within the anatomical ROIs (SVC *p*_*FWE*_ < 0.10), and these high‐resolution findings are explored thoroughly in consideration of their novelty. Regarding state generalization, the loci of interaction effects included the anterior caudate nucleus (SVC *p*_FWE_ < 0.10) as well as both the SN and the ventral tegmental area (VTA) (*p* < .005) (Figure S7a). Effects of action generalization were found elsewhere in both the SN and the VTA (*p* < .005) (Figure S7b).

A control analysis determined that these findings were specific to generalization as opposed to nonspecific successful learning. Interaction effects between RPE signals and the learning rate (i.e., *α/τ*) (Tables S23–S25; see S25 for summary) were examined to check for overlap with generalization effects, which would suggest a confound if present. There was in fact no such overlap for either the 3‐T Face/House or 7‐T Color/Motion results (*p* > .005). Instead, both data sets confirmed the dorsal caudate nucleus as a site where RPE signals are more directly related to learning performance (*p* < .005), replicating previous findings (Colas et al., 2017; Schönberg et al., 2007).

## 3 DISCUSSION

Supported by multisite, multifield fMRI in conjunction with computational modeling of both behavioral and neural dynamics, the findings herein have confirmed the merits of the GRL model as representative of a class of RL models obscuring the boundary between model‐free caching and model‐based inference. This conceptual ambiguity suggests a false dichotomy in the notion of a unidimensional spectrum between these antipodes with ostensible mutual exclusivity; putative roles for dopamine are also complicated by such ambiguity (Botvinick, 2012; Bromberg‐Martin et al., 2010; Collins & Cockburn, 2020; da Silva & Hare, 2020; Delgado & Dickerson, 2012; Doll et al., 2012; Eckstein & Collins, 2020; Gardner et al., 2018; Langdon et al., 2018; Nakahara, 2014; Nakahara & Hikosaka, 2012; O'Doherty, 2012; Sadacca et al., 2016; Schultz, 2013). For a structured but challenging learning task that lends itself to implicit generalization with a cognitive map, augmenting the classical RL framework (Sutton & Barto, 1998) with associative and discriminative forms of state and action generalization improved the exposition of human performance at the rigorous individual level—here including idiosyncrasies in generalization. Rather than the unambiguously model‐based approaches of the SPE or the HMM that proved less effective here, the intuition of the GRL model parsimoniously remains within the established bounds of RL and its fundamental RPE signal that is integral to computational analysis of neurophysiology in mesostriatal and corticostriatal circuits. Moreover, this work marks a juxtapositional demonstration of the potential of high‐field fMRI for these particular signals and neural systems—especially with respect to the dopaminergic midbrain (cf. Colizoli et al., 2021; de Hollander et al., 2017; Fontanesi, Gluth, Rieskamp, et al., 2019).

Our tripartite neural model—representing interrelated value, decision, and learning signals in parallel—stands among the novel technical and computational contributions made here. Guided by precedents for classic RL (Colas et al., 2017), dynamic RPE signals derived from the GRL model were localized within the dopaminergic midbrain and both ventral and dorsal areas of the striatum. Dissociable value signals from the GRL model could be identified simultaneously in other subregions of the ventral striatum as well as in vmPFC and PCC, amounting to all of the areas hypothesized with meta‐analytic priors (Bartra et al., 2013; Clithero & Rangel, 2014). Neural correlates of RT were also controlled for and validated in MFC (Yarkoni et al., 2009) as a proxy for decision‐making signals. Furthermore, effects of state and action generalization per se were evident in modulation of RPE signaling in the basal ganglia that could only be accounted for with the GRL model as opposed to basic RL. These interaction effects reflect relaying of RPE signals to representations of other states and actions rather than merely that of the state‐action pair experienced at a given moment. The hippocampus was also identified as a hub for mediating this generalization across representations that here would correspond to motor and premotor cortex or visual cortex, including the FFA, the PPA, V4, and MT.

Generalization of knowledge is a ubiquitous cognitive phenomenon that is essential for processing the plethora of different stimuli that organisms encounter (Bush & Mosteller, 1951b; Ghirlanda & Enquist, 2003; Harlow, 1949; Shepard, 1957, 1987; Tenenbaum & Griffiths, 2001; Tversky, 1977), but the broad concept of generalization can manifest itself in myriad different ways depending on the situation. For example, whereas the present paradigm contrasts associative generalization against discriminative generalization among temporally interleaved states and actions that are explored in parallel, alternative paradigms have instead focused on more straightforward associative generalization from familiar or proximal stimuli to novel or distal stimuli of varying apparent similarity (Collins & Frank, 2013; Doll, Duncan, et al., 2015; Doll, Shohamy, & Daw, 2015; Gershman, 2017; Gershman & Niv, 2015; Kahnt et al., 2012; Kahnt & Tobler, 2016; Karagoz et al., 2022; Kool et al., 2016, 2017, 2018; Lesaint et al., 2014; Norbury et al., 2018; Stojić et al., 2020; Tomov et al., 2018; van Dam & Ernst, 2015). The discriminative generalization of GRL is distinguished from such feature‐based generalization in the arbitrary mapping of abstract states, thus going beyond simply generalizing across common perceptual features of cues or linked outcomes without state discrimination. Here, the state category is not processed as a unitary representation but rather as a set of representations with a cognitive map (whether implicit or explicit) that discriminates and determines relations within the set as defined by a hierarchical metastate. Another distinction can be drawn between generalized information and counterfactual information that is made explicit with directly observed feedback rather than inferred from assumptions of interdependence, producing contextual effects such as fictive regret or framing (Camille et al., 2004; Coricelli et al., 2005; D'Ardenne et al., 2013; Li & Daw, 2011; Lohrenz et al., 2007; Montague et al., 2006; Palminteri et al., 2015; Palminteri, Lefebvre, et al., 2017; Pischedda et al., 2020). For this and other reasons (see below), the present label of “generalized RL” is more precise than “counterfactual RL”, for example. The GRL model aims toward broader theoretical advancement for a still‐nascent perspective on quasi‐model‐based extensions of model‐free RL, and two dichotomies are formalized in doing so: state versus action generalization and associative versus discriminative generalization, where in this case the latter translates to suboptimal overgeneralization (or conflation) versus optimal inference or pseudoinference.

Not only these dichotomies but also the particular delta‐learning rules of the two‐dimensional GRL model distinguish it from previous modifications of model‐free RL. Often arrived at without the due diligence of model comparison, some modifications have simply yoked value representations—for example, *Q*_*t*_ *(s*_*t*_ *,a*_1_ *)* ≡ *−Q*_*t*_ *(s*_*t*_ *,a*_2_ *)*—or otherwise incorporated only one type of generalization (Aquino et al., 2020; Balcarras & Womelsdorf, 2016; Ballard et al., 2019; Baram et al., 2021; Charpentier et al., 2020; Collette et al., 2017; Daw & Shohamy, 2008; Gläscher et al., 2009; Hampton et al., 2007; Hauser et al., 2014, 2015; Lesage & Verguts, 2021; Liu et al., 2021; Matsumoto et al., 2007; Mattar & Daw, 2018; Reiter et al., 2017; Vinckier et al., 2016; Wimmer et al., 2012; Zaki et al., 2016). Moreover, such models are often formulated without parameterization (e.g., *g*_*A*_ = −1) or with a second, counterfactual RPE inverting the only outcome (i.e., *r′* = *−r* or *r′ = 0* for *r* > 0) in parallel—and, by extension, multiple RPEs as required—as opposed to the current algorithmic scheme of GRL with weighted duplications of the original RPE signal to be relayed to parallel representations of estimated values. The assumption of an inverted counterfactual outcome is not universally applicable and can also create scaling problems for value signals, including disproportionate RPEs as well as even illogical probability estimates (*P* < 0 or *P* > 1) or negative value estimates despite strictly positive outcomes (or vice versa). This issue is especially problematic for modeling that is less abstract in its application to an actual neural system. Another issue is counterfactual learning via separate hypothetical RPE signals each subtracting their respective reward predictions, which is less tractable for modeling than the present implementation based on a parameterized heuristic with relayed duplication of a single RPE signal: Relaying—or perhaps even multiplexing (cf. Nakahara, 2014; Nakahara & Hikosaka, 2012)—is less computationally demanding and more parsimonious. (A multiplexed signal in this context would additionally specify how the RPE is to be generalized.) It is less plausible that any number of hypothetical RPE signals could be distinguished in the brain in parallel with assumptions of unique RPE signals for each of the states or actions updated per single action performed—and particularly implausible for an action space that is more continuous rather than discrete. The GRL model therefore eschews a true “counterfactual RPE” in favor of a “generalized RPE” (but still can be regarded as a version of counterfactual learning). This formulation is readily scaled up for environments with arbitrary complexity in the numbers of state‐action pairs or category‐state‐action triplets.

This efficient approach is analogous to the “TD(*λ*)” eligibility trace (Dayan, 1992; Dayan & Sejnowski, 1994; Klopf, 1972; Sutton, 1988; Sutton & Barto, 1981, 1998) that forgoes separate RPEs for antecedent events in favor of more conservatively duplicating, reweighting, and relaying the current RPE back in time with decay along the memory trace (Figure 2). This perspective of TD(*λ*) as temporal generalization could consider it as a particular form of associative generalization across linear time, which evokes the temporal spread of the law of effect (Thorndike, 1911, 1933). In addition to the dimension of time, the RPE in this “GRL(*λ*)” model is generalized across dimensions in the abstract space of state and action representations with nonlinear temporal mapping (cf. Jocham et al., 2016). This topological space could be encoded in nonspatial cognitive maps analogous to the location‐based spatial maps (Moser et al., 2008; O'Keefe & Nadel, 1978) represented in the medial temporal lobe, including the hippocampus and entorhinal cortex (Ballard et al., 2019; Baram et al., 2021; Behrens et al., 2018; Bernardi et al., 2020; Cazé et al., 2018; Daw & Shohamy, 2008; Gerraty et al., 2014; Liu et al., 2019, 2021; Mattar & Daw, 2018; Momennejad et al., 2018; Park et al., 2020; Wimmer et al., 2012; Wimmer & Shohamy, 2012). Such abstract cognitive mapping (Tolman, 1948) follows from the intrinsic topology of mental representations as postulated in field theory (Lewin, 1935, 1936). Simulated replay of experienced (or even hypothetical) events by the hippocampus is a potential mechanism for recapitulating task‐relevant information (Cazé et al., 2018; Eldar et al., 2020; Gershman et al., 2014; Kurth‐Nelson et al., 2016; Liu et al., 2019, 2021; Mattar & Daw, 2018; Momennejad et al., 2018; Schuck & Niv, 2019; Wimmer et al., 2020), which for the present purposes could contribute to the associations underpinning generalization. This hippocampal replay is reminiscent of the model‐free but quasi‐model‐based “Dyna” architecture from machine learning that approximates model‐based dynamic programming as indirect RL with quasi‐inferential iterations of simulated experiences (Sutton, 1990, 1991).

The algorithm described here follows a rising trend toward model‐based alternatives to the model‐free RL framework, including hybrid models that integrate multiple learning systems (Daw et al., 2005; Doll et al., 2012; O'Doherty et al., 2017, 2021). With the simplest case of two systems receiving the most examination, the theoretical dichotomy of model‐free and model‐based processes is analogous to an extent with that between habitual (Pavlov, 1927; Thorndike, 1898, 1911) and goal‐directed (Tolman, 1948) learning. The most commonly studied domain of model‐based inference has typically been characterized with a modular system engaged in explicit forward planning of future behaviors in parallel with the caching of model‐free associations (Charpentier et al., 2020; Daw et al., 2005, 2011; Gläscher et al., 2010; Lee et al., 2014). For example, dynamic programming can achieve optimal goal‐directed behavior in a multistep Markov decision process (MDP) with the learning of transition functions for states and state‐action pairs (Bellman, 1957; Sutton & Barto, 1998), which can be arrived at with computation of another type of SPE analogous to the RPE (Gläscher et al., 2010; Lee et al., 2014). However, the broader model‐based umbrella can also encompass cognitive maps and certain mechanisms for generalization in learning, including the HMM (Ghahramani, 2001; Hampton et al., 2006; Prévost et al., 2013) and other Bayesian processes (Tenenbaum & Griffiths, 2001) as well as the novel formulation of the SPE developed here (with an “MPE” for metastates). Such generalization represents a potential domain of overlap between model‐based and model‐free processes (Bromberg‐Martin et al., 2010; Doll et al., 2012; Doll, Duncan, et al., 2015; Doll, Shohamy, & Daw, 2015; Hampton et al., 2006, 2007; Karagoz et al., 2022; Kool et al., 2016, 2017, 2018; Liu et al., 2021; Mattar & Daw, 2018; O'Doherty, 2012; Sadacca et al., 2016; Wimmer et al., 2012; Wunderlich et al., 2011).

As GRL features an implicit model for generalization while prioritizing parsimony and computational efficiency, this scheme—by way of analogy to Dyna—is not neatly encapsulated by either extreme of the model‐free/model‐based dichotomy. GRL thus also joins the ranks of the successor‐representation algorithms that operate with analogous ambiguity in shortcut solutions based on a compressed transition function, which would be more applicable to a learning task with multiple steps per episode (or trial) (Akam et al., 2015; Dayan, 1993; Momennejad et al., 2017; Russek et al., 2017, 2021). Likewise, whereas GRL frugally accounts for generalization across states and actions within a task, by extension, heuristic algorithms based on reward‐predictive state abstractions have been proposed for generalization across tasks, which can be represented by unique transition functions (Franklin & Frank, 2018; Lehnert et al., 2020; Li et al., 2006).

As the GRL model forgoes supplanting model‐free RL altogether, so too does it forgo complementing a model‐free system with a model‐based system operating in parallel (cf. Doll, Duncan, et al., 2015; Doll, Shohamy, & Daw, 2015; Karagoz et al., 2022; Kool et al., 2016, 2017, 2018)—instead opting for quasi‐model‐based augmentation of model‐free RL that is still effectively characterized by a single system. From the perspective of control theory, an HMM (Ghahramani, 2001) can provide an optimal Bayesian solution to this generalization problem with a fully model‐based approach to structural inference (Hampton et al., 2006; Prévost et al., 2013), but this avenue entails assumptions of more complex computations as well as ambiguity concerning the physical implementation of the implied neural mechanisms (cf. Gläscher et al., 2009; Hampton et al., 2007); the latter can be an obstacle to achieving comprehensive triangulation across levels of analysis (Marr, 1982). Although engagement of multiple systems for a model‐free and model‐based hybrid remains within the realm of possibility, going down this route of additional moving parts with two systems—let alone in excess of two—is even more problematic in this regard (cf. Daw et al., 2011). Presently, GRL outperformed dual‐systems alternatives despite whatever viability they could have. In addition to the virtue of Occam's razor on the theoretical side of parsimonious modeling (Myung, 2000), there are practical advantages for fitting and interpretability with the simpler RL‐based approach in settings where fully model‐based learning is less essential or even counterproductive for a dynamic environment. That is, a primarily model‐free strategy is often sufficient for at least near‐optimal performance, and humans (like other animals) often fail to achieve optimal performance anyway, as was evident here and in another study (Aquino et al., 2020). These benefits extend to modeling of not only behavior but also neurophysiology, where a parsimonious model grounded in well‐defined concepts can provide a stable foundation with utility such as for interpreting performance of varied tasks or for identifying nodes in relevant networks (Bassett et al., 2018; Gerraty et al., 2018; Mattar et al., 2018). In this case, subjective value and the RPE naturally fill roles as part of a trichotomy of value, decision, and learning signals in the brain that collectively function as the interface between sensory input and motor output.

This modeling sets the stage for further inquiry concerning how arbitration among strategies for generalization might be implemented; here lies an analogy with reliability‐based arbitration among modular model‐free and model‐based systems to integrate information across a “mixture of experts” (Charpentier et al., 2020; Daw et al., 2005; Lee et al., 2014; O'Doherty et al., 2017; O'Doherty et al., 2021) as in machine learning (Hamrick et al., 2017; Jacobs et al., 1991; Masoudnia & Ebrahimpour, 2014; Yuksel et al., 2012). Instead of tracking absolute prediction errors or entropy in updates of cached value functions or modeled transition functions, the “experts” for generalization would be concerned with tracking regularities or irregularities across inputs for the structural models embedded in such functions (as well as tracking task demands warranting effort) (cf. Hampton et al., 2006; Karagoz et al., 2022; Kool et al., 2017, 2018; Lehnert et al., 2020; Liu et al., 2021; Mattar & Daw, 2018; Prévost et al., 2013; Schulz et al., 2018, 2020; Wu et al., 2019; Wu, Schulz, Garvert, et al., 2018; Wu, Schulz, Speekenbrink, et al., 2018; Wunderlich et al., 2011). However, modeling such arbitrated metalearning for the present experiment is precluded by certain practical limitations—in particular, the issue of both forms of discriminative generalization always being optimal strategies and hence not being modulated with sufficient variability. The GRL model in its current form does not distinguish between prior assumptions about generalizable structure and learned information about structure acquired through serial observations. Feasibly translating the static generalization effects of the current GRL model to dynamical generalization processes will require an experimental paradigm with dynamic structure more directly catered to manipulating cognitive models for generalization—for example, including alternation across correlation, anticorrelation, and independence. Nevertheless, the presently static generalization parameters suffice as an initial proof of concept for such extensions of RL and in particular both associative and discriminative generalization across both states and actions.

This endeavor has justified the GRL model as a viable and practical tool in a growing model space that need not be limited to purely model‐free learning and purely model‐based learning. These results localize a modular network of brain regions that orchestrate evaluation and value‐based learning and decision making in a setting characterized by generalizable patterns across both states and actions. By identifying the nodes of a network mediating reinforcement learning and concomitant generalization to link representations of stimuli or motor responses within a cognitive map, this (computational) model‐based mapping lays the groundwork for further investigation of network dynamics (Bassett et al., 2018; Gerraty et al., 2018; Mattar et al., 2018) with the potential to yield yet more comprehensive understanding of the causal chain of information flow between sensation and action in a reward‐based environment that is noisy and dynamic but also predictably structured.

## 4 METHODS

### 4.1 Participants

Forty‐seven (male:female = 27:20; age: *M* = 25.5 y, SD = 4.9 y) and twenty‐two (male:female = 12:10; age: *M* = 28.0 y, SD = 6.0 y) human participants volunteered for the 3‐T and 7‐T versions of the study, respectively. This collaborative multisite study was conducted at six separate facilities for magnetic‐resonance imaging (MRI), such that participants were recruited from the respective universities and local communities of each laboratory. All participants were screened for MRI contraindications; all were right‐handed and generally healthy adults between 18 and 43 years old. Participants in the 7‐T Color/Motion version were also screened for color blindness. Participants provided informed written consent according to protocols approved by the respective Institutional Review Boards of each scanning site—namely, the California Institute of Technology; Columbia University; New York University; the University of Pennsylvania; the University of California, Santa Barbara; and the University of Southern California. Upon completing the study, participants were paid $10 for minimizing head movement plus the amount of money earned within the task.

### 4.2 Experimental procedures: 3‐T Face/House version

Shown in Figure 1 is a schematic of the hierarchical reversal‐learning task that includes outcome probabilities for every combination of state and action within one of 12 blocks defined by said probabilities. (A complete session of 12 blocks is detailed in Figure 7.) At the onset of each episodic (i.e., separate) trial, one of four predictive cues was presented with equal probability, but trials were also ordered in a series of randomized and counterbalanced quartets that each included four cues representing separate states. These quartets were constrained such that a cue never appeared in consecutive trials. The onset of a trial was marked by an image of a face or a house appearing against a white background subtending 8.1° × 8.1° of visual angle at the center of the display—first flanked by two white arrows to the left and right each subtending 1.0° × 8.1° and centered at an eccentricity of 4.9°. The participant was allotted 2 s to respond to this two‐armed bandit by pressing one of two buttons with the corresponding index finger of either the left or right hand. To confirm the response while minimizing eye movement, the arrow corresponding to the nonchosen action was removed from the display between the time of response and stimulus offset. A fixed interstimulus interval (ISI) of 3 s separated the cue and the outcome. In consideration of the sensitivity of a TD learning algorithm to the timing of outcomes (McClure et al., 2003; O'Doherty et al., 2003; O'Doherty et al., 2004; Sutton, 1988; Sutton & Barto, 1998), jitter—otherwise typical of rapid event‐related designs in functional MRI (fMRI)—was forgone with the ISI in favor of a design that induced stable prediction‐error signals.

The transition probabilities for the action given the state determined whether the outcome following the ISI was a rewarded state or a nonrewarded state. Delivery of an actual reward of $0.30 was symbolized by a black dollar sign against a white background again subtending 8.1° × 8.1° for 1 s, whereas a scrambled dollar sign signified an absence of monetary reward for that trial. This scrambled image was generated by randomly rearranging segments of the dollar sign as a regular 8 × 15 grid. Only a white fixation cross subtending 0.7° × 0.7° of visual angle was presented at the center of a black background throughout the ISI and the intertrial interval (ITI). This fixation cross also remained in the foreground of the display with a black outline during stimulus presentation. The duration of the jittered ITI was drawn without replacement within a run from a discrete uniform distribution ranging from 3 to 7 s in increments of 41.7 ms. If the participant failed to respond in time, the nonrewarded outcome appeared immediately as the fixation cross turned red for 1 s; the ISI would then be merged with the subsequent ITI.

Representing each active state, four new cues were assigned randomly every run with two pairs of images each respectively drawn from two state categories. In the 3‐T version of the experiment, these categories were faces and houses, which share common low‐level visual features as a control. Face stimuli were extracted from the Chicago Face Database (Ma et al., 2015), which also includes subjective ratings of the stimuli along various dimensions. A set of eight face images were selected for depicting an adult male who was consistently classified in the “White” ethnic group (*M* = 97.5%, SD = 2.0%) and rated as neither especially attractive nor especially unattractive (Likert scale [1, 7]: *M* = 3.55, SD = 0.23). All portraits were intended to display a neutral facial expression. These selection criteria minimized the potential for hedonic evaluation of the arbitrary stimuli themselves to interfere with experimental manipulations of value‐based associations based on rewards in the task (Chien et al., 2016). In keeping with these controls, all images were converted to grayscale. House stimuli were extracted from the DalHouses database (Filliter et al., 2016), which included subjective ratings of facial pareidolia and other attributes. A set of eight house images were selected for being rated as minimally facelike (Likert scale [1, 7]: *M* = 2.22, SD = 0.10) and being distinctive relative to the rest of the set. As the human brain is endowed with innate expertise for recognizing faces but not houses (Kanwisher, 2000), the face stimuli were selected to be homogenous while the house stimuli were instead selected to maximize the heterogeneity of the set.

Rather than sheer randomness, which especially limits interpretation of individual differences, meticulously controlled counterbalancing was crucial for eliminating confounds within and across individual sessions. For each participant, different conditions were randomized and counterbalanced to evenly distribute rewards for categories, states, and actions in a factorial design defining 12 blocks that included hierarchical reversals of instrumental learning. Four scanning runs including three blocks each and 32 trials per block made for 384 trials in total. (Prior to the actual experiment, the participant completed 10‐trial practice sessions with separate stimuli both outside and inside the scanner.)

Nearly attaining a 3 × 2 × 4 design for the 12 blocks, the 3 × 2 and 3 × 4 crosses were fully counterbalanced while the 2 × 4 cross could only be partially balanced given the number of blocks. By virtue of this counterbalancing, choosing the same action for every single trial of the session was guaranteed to yield exactly half of the available rewards. Likewise, each state category preceded exactly half of the available rewards within each run. Moreover, with reward probabilities in units of sixteenths, each run included exactly or nearly one quarter of the rewards for the entire session. Yet the reward probabilities for state‐action pairs fluctuated from block to block so as to facilitate variability in the dynamics of neural signals of interest. Across the session, what remained constant amid these fluctuations was the anticorrelational pattern between actions within a state and between states within a category. The categories were independent of each other without any such structured pattern between them.

The first condition (“3” in the 3 × 2 × 4 design), having three possibilities also counterbalanced within a run, determined whether the face category had greater, lesser, or equivalent value relative to the house category. For the unequal conditions, the category with greater value included reward probabilities of 62.5% and 100%, whereas the category with lesser value included reward probabilities of only 43.75%. For the equal condition, both categories included reward probabilities of 43.75% and 81.25%. These exact probabilities were all divisible by sixteenths and so were evenly split between two 32‐trial blocks with 8 trials per state. (For the odd probabilities of 43.75% and 81.25%, the more‐rewarded halves of the distributions were evenly distributed within a condition sampled across runs: The net probability of 43.75% (7/16) was the average of 37.5% (6/16) and 50% (8/16), and net 81.25% (13/16) was the average of 75% (12/16) and 87.5% (14/16).) A nonzero reward probability was only assigned to one action per state, always leaving an alternative action with zero probability of reward. This complementarity between actions within a state was designed to reveal action generalization.

The second condition (“2”), having two possibilities partially counterbalanced with a 2:1 ratio within a run, concerned which state (arbitrarily “A” or “B”) had the greater value within a category if the category included two different reward probabilities for a given block.

The third condition (“4”), having four possibilities, concerned the mapping of a category's reward probabilities to actions, such that the two states (“A” and “B”) within a category always symmetrically provided rewards for opposite actions. This complementarity between states within a category was designed to reveal state generalization. The possibilities for this condition could be summarized across all four active states like so: “LR&LR”, “LR&RL”, “RL&LR”, or “RL&RL”, where the example of “LR&RL” can be expanded as “AL/BR & AR/BL” for the binary hierarchical metastates of the face and house categories, respectively. That is, “LR&RL” (or “AL/BR & AR/BL”) would mean that the left action is rewarded for face A and house B while the right action is rewarded for face B and house A.

Between blocks, the design was constrained for a single remapping—that is, reversals of rewarded actions within only one category—to mark the onset of a new block within a run. The two categories were remapped in turn in a random order counterbalanced across runs, such that each category had one between‐block remapping per run. Although the participant was informed that the reward probabilities could change throughout the session, no explicit indications were provided as to how or when such changes might occur.

Stimuli were projected onto a screen that was viewed with an angled mirror in the MRI scanner. The viewing distance was 100 cm in the case of the Caltech sample, which served as the basis for the approximate stimulus sizes reported here, but there was slight variability in stimulus sizes across laboratories. The display was presented with a resolution of 1024 × 768 pixels and a refresh rate of 60 Hz. The primary stimuli had a resolution of 375 × 375 pixels. The interface was programmed with MATLAB (MathWorks) and the Psychophysics Toolbox (Brainard, 1997).

### 4.3 Experimental procedures: 7‐T Color/Motion version

Conducted in parallel, the second version of the experiment was mostly matched to the first but was not entirely identical. Only differences between versions are emphasized in this section.

This 7‐T version substituted dynamic colors and directions of motion in lieu of faces and houses as state categories. Moreover, these color and motion stimuli were not replaced every run as with the 3‐T version's faces and houses. Although the two pairs of stimuli comprising the two categories remained constant across the entire session, the factorial design of the 3‐T version was preserved such that the reward probabilities for these constant states still rotated as before. The 7‐T version was fully counterbalanced as before, and the constant cues allowed for even further counterbalancing such that each cue preceded exactly one quarter of the available rewards in a session.

The color stimuli were flickering dot arrays that alternated between two colors for each state. One stimulus alternated six times between red dot arrays and green arrays at a rate of 6 Hz, and the other similarly alternated between blue and yellow dots. These color stimuli were essentially arranged as static frames of the motion category's random‐dot kinetograms (Newsome & Paré, 1988). Apparent motion was generated by displacement of the dots in a consistent direction every frame with 100% motion coherence. The two states in this category were represented with upward or downward motion, respectively. The speed of displacement was 2.1° per second.

For both color and motion stimuli, unique dot arrays were randomly generated with every trial. These arrays contained over 100 square dots randomly positioned against a black background. The array was framed by a gray square subtending 4.1° × 4.1° at the center of the display—first flanked by two gray arrows each subtending 0.5° × 4.1° and centered at an eccentricity of 2.5°. If the participant failed to respond in time, the nonrewarded outcome appeared immediately as the white fixation cross subtending 0.4° × 0.4° turned gray.

### 4.4 Data acquisition: 3‐T Face/House version

For the first version of the experiment, MRI data were collected with a common set of protocols across five sites housing 3‐tesla Magnetom Prisma scanners (Siemens Medical Solutions, Malvern, PA) equipped with 2‐channel body‐transmit and 32‐channel head‐receive coils. The first structural volume covered the whole brain and was acquired to guide subsequent functional imaging with a single‐inversion T1‐weighted (T1w) 3‐dimensional (3D) magnetization‐prepared rapid gradient‐echo (MPRAGE) sequence that had the following parameters: repetition time (TR): 2400 ms, echo time (TE): 2.32 ms, inversion time (TI): 800 ms, RAGE flip angle (FA): 10°, in‐plane GRAPPA acceleration factor (R): 2, voxel: 0.9 mm isotropic, field of view (FOV): 187 × 230 × 230 mm. A second structural volume was acquired after the experiment with a T2‐weighted (T2w) 3D SPACE (“sampling perfection with application‐optimized contrasts using different flip‐angle evolutions”) sequence that had the following parameters: TR: 3200 ms, TE: 564 ms, FA: variable, R: 2, voxel: 0.9 mm isotropic, FOV: 187 × 230 × 230 mm.

During the experiment, functional images were acquired from the whole brain using a blood‐oxygen‐level‐dependent (BOLD) contrast with a T2*‐weighted gradient‐echo echo‐planar imaging (EPI) sequence (Center for Magnetic Resonance Research, Department of Radiology, University of Minnesota) featuring both in‐plane GRAPPA (“generalized autocalibrating partially parallel acquisitions”) (Griswold et al., 2002) and multiband slice excitation (Feinberg & Setsompop, 2013; Moeller et al., 2010) and having the following parameters: TR: 1120 ms, TE: 30 ms, FA: 54°, multiband acceleration factor (M): 4, R: 2, voxel: 2.0 mm isotropic, FOV: 144 × 192 × 192 mm. Off‐resonance distortion correction was based on phase‐encoding polarity‐reversed spin‐echo EPI image pairs with geometry, acceleration, and EPI echo spacing all matched to the BOLD fMRI series (TR: 5130 ms, TE: 41.4 ms, FA: 90°, voxel: 2.0 mm isotropic, FOV: 144 × 192 × 192 mm). The session consisted of four functional runs each having a duration of 17.7 min and each preceded by field maps.

Peripheral cardiac and respiratory signals were recorded during scanning by way of scanner‐integrated wireless sensors. The pulse sensor was attached to the ring finger of either the left hand or the right hand, such that this factor was counterbalanced across subjects. The pneumatic sensor was secured under a strap to measure external displacement of the lungs.

### 4.5 Data acquisition: 7‐T Color/Motion version

For the second version of the experiment, MRI data were collected at a single site using a 7‐tesla Siemens Magnetom Terra scanner equipped with a single‐channel head‐transmit volume coil and a 32‐channel head‐receive coil. In light of the tradeoff between the signal‐to‐noise ratio (SNR) or the contrast‐to‐noise ratio (CNR) and either spatial or temporal resolution, high‐field neuroimaging allows for a superior SNR and CNR (De Martino et al., 2018; Dumoulin et al., 2018; Torrisi et al., 2018; Uğurbil, 2018) that could be relied upon here to achieve higher spatial resolution. In the interest of maximizing spatial resolution at 7 T, temporal resolution for the EPI sequence was also compromised somewhat relative to the 3‐T protocol, but simultaneous multislice acquisition still enabled viable temporal resolution. Enhancing the volumetric resolution by a factor of 4.6, this approach boasted more precise discernment of mesencephalic nuclei (Eapen et al., 2011) in particular. Otherwise, the 7‐T protocols were matched to the 3‐T protocols as closely as possible to allow for direct comparison.

The session again began with a whole‐brain structural volume acquired using a dual‐inversion T1w 3D “MP2RAGE” sequence (Choi et al., 2019) (TR: 4010 ms, TE: 2.86 ms, TI_1_: 1050 ms, TI_2_: 3200 ms, FA_1_: 6°, FA_2_: 4°, R: 2, voxel: 0.8 mm isotropic, FOV: 179 × 220 × 220 mm). The complementary structural volume was acquired after the experiment with a T2w 3D SPACE sequence (TR: 4270 ms, TE: 315 ms, FA: variable, R: 3, voxel: 0.7 mm isotropic, FOV: 168 × 224 × 224 mm).

During the experiment, functional images were acquired from the whole brain using a BOLD contrast with a T2*‐weighted gradient‐echo EPI sequence (Center for Magnetic Resonance Research, Department of Radiology, University of Minnesota) (TR: 1960 ms, TE: 22 ms, FA: 45°, M: 4, R: 2, voxel: 1.2 mm isotropic, FOV: 125 × 192 × 192 mm). Off‐resonance distortion correction was based on phase‐encoding polarity‐reversed spin‐echo EPI image pairs with geometry, acceleration, and EPI echo spacing all matched to the BOLD fMRI series (TR: 7680 ms, TE: 30.6 ms, FA: 90°, voxel: 1.2 mm isotropic, FOV: 125 × 192 × 192 mm). Cardiac and respiratory signals were again recorded via wireless sensors during scanning.

### 4.6 Data preprocessing

Real‐valued, signed T1w images generated by the MP2RAGE sequence at 7 T were first masked with Otsu thresholding (Otsu, 1979) of auxiliary magnitude data generated by the same sequence, thereby eliminating background noise in surrounding air. This initial step ensured compatibility with subsequent structural preprocessing as part of a common pipeline applied to both 3‐T and 7‐T images.

Neuroimaging data were primarily preprocessed using fMRIPrep version 1.2.5 (Esteban et al., 2019). This software package includes elements from the FMRIB Software Library (FSL) v5.0.9 (Centre for fMRI of the Brain, University of Oxford) (Smith et al., 2004), Advanced Normalization Tools (ANTs) v2.1.0 (Avants et al., 2010), FreeSurfer v6.0.1 (Laboratory for Computational Neuroimaging, Athinoula A. Martinos Center for Biomedical Imaging) (Fischl, 2012), and Analysis of Functional NeuroImages (AFNI) v16.2.07 (Scientific and Statistical Computing Core, National Institute of Mental Health) (Cox, 1996)—all compiled with the Nipype interface (Gorgolewski et al., 2011) and often facilitated by the Nilearn toolbox (Abraham et al., 2014).

Rather than utilizing the scanning system's internal bias‐field correction, each T1w volume was first corrected for intensity nonuniformity using “N4BiasFieldCorrection” (ANTs) (Tustison et al., 2010). Skull stripping was then performed with “antsBrainExtraction” (ANTs) using the Open Access Series of Imaging Studies (OASIS) template (Marcus et al., 2007). Incorporating information from both T1w and T2w volumes, brain surfaces were reconstructed using “recon‐all” (FreeSurfer) (Dale et al., 1999). The previously estimated brain mask was refined with a custom variation of the method to reconcile ANTs‐derived and FreeSurfer‐derived segmentations of cortical gray matter from Mindboggle (Klein et al., 2017). All images were converted to the common Montreal Neurological Institute (MNI) space (Collins et al., 1994). Spatial normalization to the MNI152‐based ICBM 2009c Nonlinear Asymmetric template (Fonov et al., 2009) was performed through nonlinear registration with “antsRegistration” (ANTs) (Avants et al., 2008), combining brain‐extracted versions of both the T1w volume and the template. Segmentation of brain tissue into cerebrospinal fluid (CSF), white matter, and gray matter was performed on the brain‐extracted T1w volume using FMRIB's Automated Segmentation Tool (FAST) (FSL) (Zhang et al., 2001).

For functional BOLD images, slice‐time correction was applied with “3dTshift” (AFNI). Motion correction was applied using Motion Correction with FMRIB's Linear Image Registration Tool (MCFLIRT) (FSL) (Jenkinson et al., 2002). Utilizing field maps, distortion correction and unwarping was performed with an implementation of the TOPUP technique (Andersson et al., 2003) using “3dQwarp” (AFNI). Functional volumes were coregistered to the corresponding structural T1w volume via boundary‐based registration (Greve & Fischl, 2009) with 9 degrees of freedom, as implemented by “bbregister” (FreeSurfer). All transformations for motion correction, distortion correction, BOLD‐to‐T1 coregistration, and T1‐to‐template coregistration were concatenated and applied in a single step using “antsApplyTransforms” (ANTs) with Lanczos interpolation.

First using only imaging data itself for denoising, dynamics of sources of noise such as head motion, physiological events, and measurement (i.e., scanner) artifacts were estimated by different approaches to generate confound regressors of no interest for the general linear model (GLM). Notably, BOLD signals throughout the brainstem have an intrinsically low SNR and a low CNR and are especially susceptible to physiological artifacts (Barry et al., 2013; Dagli et al., 1999; de Hollander et al., 2015, 2017; Düzel et al., 2009, 2015; Enzmann & Pelc, 1992; Soellinger et al., 2007). The proximity of the pulsatile interpeduncular cistern to the tegmentum further compromises signals of purely neural origin in the key region of the dopaminergic midbrain. To address these issues as well as possible differences in output between the different scanners for multisite fMRI, further extensive efforts were dedicated to eliminating contaminant noise as follows.

Six rigid‐body motion parameters—corresponding to three axes for translation and three for rotation—were estimated relative to a reference image and subsequently added to the design matrix. Time series of signals averaged within the CSF mask, within the white‐matter mask, or globally across the entire brain mask were included next. Framewise displacement quantified bulk head motion within each functional run (Power et al., 2012, 2014). An index for the rate of signal change across the entire brain was provided with the standardized temporal derivative of root‐mean‐squared variance over voxels (DVARS) (Power et al., 2012, 2014; Smyser et al., 2010). Initial time points identified as nonsteady states according to global signals were marked with unique indicator variables for each outlier volume. Furthermore, these outliers were omitted from the following denoising procedures.

For temporal high‐pass filtering, a discrete cosine transform (DCT) (Ahmed et al., 1974) was employed to detect low‐frequency signal drift. Fifteen DCT basis functions were generated as regressors after omitting the aforementioned outliers. The CompCor method for denoising relied on principal‐component analysis, and principal components were generated with the two variants of the algorithm—namely, “temporal” (tCompCor) and “anatomical” (aCompCor) (Behzadi et al., 2007). A mask to exclude signals with cortical origins was first obtained by eroding the brain mask so as to ensure it only contained subcortical structures. Six tCompCor components were then estimated with voxels above the 95^th^ percentile for signal variability within the eroded subcortical mask. Another six aCompCor components were estimated within the intersection of the subcortical mask and the union of CSF and white‐matter masks in T1w space after projection to the native space of each functional run. Employing probabilistic spatial independent‐component analysis (ICA) as implemented by Multivariate Exploratory Linear Decomposition into Independent Components (MELODIC) (FSL) (Beckmann & Smith, 2004), the “aggressive” ICA‐based strategy for Automatic Removal of Motion Artifacts (ICA‐AROMA) (Pruim et al., 2015) was utilized to distinguish signal and noise components with dimensionality constrained to a maximum of 200 components.

Additional preprocessing was performed outside of fMRIPrep using Statistical Parametric Mapping (SPM) v12.7219 (Wellcome Centre for Human Neuroimaging, University College London) (Friston et al., 1995). Spatial smoothing was a final step, convolving functional images with an isotropic Gaussian kernel having a full width at half maximum (FWHM) of 6 mm for the 3‐T data set. As the aim of the 7‐T protocol was to maximize spatial resolution, the FWHM parameter was reduced to 2 mm for 7‐T data and thus preserved the fine granularity critical for detecting mesencephalic signals (Chase et al., 2015; de Hollander et al., 2015).

Moreover, the PhysIO toolbox (Kasper et al., 2017) was used to produce confound regressors derived not with imaging data but rather with peripheral cardiac and respiratory recordings. The retrospective image correction (RETROICOR) method (Glover et al., 2000) generated third‐order Fourier expansion of the cardiac phase (i.e., 6 terms for sine and cosine functions), fourth‐order expansion of the respiratory phase (8 terms), and first‐order expansion of cardiorespiratory interactions (4 terms) (as parameterized optimally in Harvey et al., 2008).

### 4.7 Computational modeling: Generalized reinforcement learning

As a quasi‐model‐based extension of model‐free “reinforcement learning” (RL) (Bush & Mosteller, 1951a; Rescorla & Wagner, 1972; Sutton & Barto, 1998) with the temporal‐difference (TD) prediction method (Dayan, 1992; Dayan & Sejnowski, 1994; Sutton, 1988), this “generalized reinforcement learning” (GRL) model introduced the dichotomies of associative versus discriminative generalization and state versus action generalization within the “critic/Q‐learner” (CQ) model (Colas et al., 2017) (Figure 2). The CQ model integrates the “critic” component of the “actor/critic” model (i.e., state‐value learning) (Barto et al., 1983, 2021; Sutton, 1984; Witten, 1977) with the Q‐learning model (i.e., action‐value learning) (Watkins, 1989; Watkins & Dayan, 1992) for passive and active states, respectively. If it were instead a question of one model or the other for a paradigm such as this having few discrete and constant actions, the Q‐learning model typically provides more accurate fits to behavior (Colas et al., 2017; Hampton et al., 2006; O'Doherty et al., 2004); the actor/critic model is instead ideal for a broad, continuous, or dynamic action space. Although further hybridization of the two algorithms has been demonstrated (Colas et al., 2017), the “actor” module of the proper “actor/critic/Q‐learner” (ACQ) model was omitted here because adding a costly free parameter for this module is less essential for a task with only one cue and two possible actions per trial; in any case, this additional complexity was beyond the scope of the present study despite the otherwise relevant handling of passive and active states. Here, this richer account of internal decision variables—one that goes beyond what is immediately evident in behavior—facilitated not only theory but also the interpretability of the neuroimaging analysis for triple dissociation of value signals, RPE signals, and decision signals modulated by value.

By design, the model is scalable for arbitrary numbers of hierarchically organized actions, states, and state categories. Yet, for clarity, the equations herein are not written in their general form (see Colas et al., 2017, for CQ(*λ*) sans generalization) but rather are tailored to only what is applicable for the present paradigm. The CQ model employs two variants of the reward‐prediction error (RPE) to learn value‐based associations—namely, the state‐value‐prediction error (SVPE) and the action‐value‐prediction error (AVPE). A more precise label for the GRL model postulated here could be the “generalized critic/Q‐learner” (GCQ) model, but generalized Q learning was the primary mechanism under scrutiny. Whereas such a critic module would feature only state generalization, the “generalized Q‐learner” (GQL) module features both state and action generalization.

To begin with, only the preparatory state of the ITI was represented by the CQ model's critic module as a passive state *s*_*0*_. As representing priors in the absence of previous associations would entail some kind of internal model, a naïve model‐free agent initializes the value of this novel state *V*_*t*_ *(s*_*0*_ *)* to zero (Li et al., 2011):

$$V_{0}\left(s_{0}\right)=0$$

The Q‐learner module is instead concerned with the active states. The beginning of a run marks initialization of action values *Q*_*t*_ *(s,a)* for all novel state‐action pairs—again at zero:

$$\forall \;\left(s,a\right):Q_{0}\left(s,a\right)=0$$

Upon transitioning from the preparatory state to an active state, an SVPE *δ* ^*V*^_*t*_ is computed as the difference between cached values per a TD algorithm. Despite not necessarily being relevant for behavior at the moment of exposure, passive states are tracked automatically because behavioral relevance can be unpredictable in the real world (Colas et al., 2017). For the sake of parsimony, the relevant input here is the prespecified action value rather than an additionally posited state value that could be represented in parallel by the critic module (cf. Colas et al., 2017). There was only one opportunity for action per episode in the present paradigm, so as far as fitting behavior is concerned, the “off‐policy” Q‐learning method could not be distinguished from an “on‐policy” alternative such as the state‐action‐reward‐state‐action (SARSA) method (Rummery & Niranjan, 1994). The former computes an RPE using the maximal value across subsequently available actions, whereas the latter computes an RPE using the value of the action actually chosen according to the current policy. Distinguishing these particular algorithms would require at least one additional step with an active state per episode. Yet, as the original standard for an action‐value‐learning algorithm, Q learning was assumed for neural modeling without further consideration of the SARSA model. Additionally, the standard discount factor *γ* was omitted here (i.e., *γ* = 1) inasmuch as only one reward could be delivered after a constant delay within episodic trials, leaving this reduced delta‐learning rule:

$$\delta_{t}^{V}=\max_{a}Q_{t}\left(s_{t+1},a\right)-V_{t}\left(s_{0}\right)$$

The value of the preparatory state is updated in turn with a fitted learning rate *α* (for 0 ≤ *α* ≤ 1) as follows:

$$V_{t+1}\left(s_{0}\right)=V_{t}\left(s_{0}\right)+\alpha \delta_{t}^{V}$$

Upon transitioning from an active state to an outcome state, an AVPE *δ* ^*Q*^_*t*_ is determined by the discrepancy between the current action‐value estimate *Q*_*t*_ *(s*_*t*_ *,a*_*t*_ *)* and the reward (or lack thereof) *r*_*t+1*_ presented in the binary outcome state:

$$\delta_{t}^{Q}=r_{t+1}-Q_{t}\left(s_{t},a_{t}\right)$$

As with any standard RL model, the value of the chosen state‐action pair is updated accordingly once the outcome has been processed. The learned information is assumed to be integrated immediately, which would be an optimal use of the time preceding the next trial:

$$Q_{t+1}\left(s_{t},a_{t}\right)=Q_{t}\left(s_{t},a_{t}\right)+\alpha \delta_{t}^{Q}$$

Considering that the reward magnitude is fixed for this paradigm, state values and action values effectively correspond to the probability of reward. To prevent duplicated and relayed prediction errors from producing an illogical expected value for probabilistic outcomes (i.e., 0 ≤ *P* ≤ 1), the function *f(x)* constrains state and action values between zero and unity as an ad‐hoc solution for this case where probability is equivalent to value. Inasmuch as a guaranteed improvement in fit in the absence of this constraint would be uninterpretable here, it is not a possibility that is considered for now: Probability estimates above unity or below zero would be meaningless as probabilities per se, and the latter would also correspond to negative valence despite an absence of punishment. Although reference dependence and normalization are mechanisms of relevance to value‐based learning (Carandini & Heeger, 2012; Kahneman & Tversky, 1979; Palminteri & Lebreton, 2021; Rangel & Clithero, 2012), the present paradigm is not suitably amenable to these complexities. When applied to the (computational) model‐based neuroimaging analysis, these simulated signals have substantial implications for the interpretation of value signals in the brain, which should be maximized with certain reward and range from neutral to appetitive rather than including anything in the aversive range. The *x* here refers to a transformation for an updated value estimate:

$$f\left(x\right)=\max\left\{0,\min\left\{1,x\right\}\right\}$$

With the addition of the “TD(*λ*)” eligibility trace (Dayan, 1992; Dayan & Sejnowski, 1994; Klopf, 1972; Sutton, 1988; Sutton & Barto, 1981, 1998), this “CQ(*λ*)” model learns more rapidly with credit assignment across serial events. The eligibility trace of the TD(*λ*) prediction‐error signal weights updates prior to the most immediate one according to the eligibility parameter *λ* (for 0 ≤ *λ* ≤ 1) as the base (i.e., inverse decay rate) of an exponential function modulating the learning rate *α*. With discretely episodic paradigms such as the present one, the eligibility trace only propagates back to the onset of the trial. Owing to this temporal generalization, the preparatory state is updated by the AVPE as well:

$$V_{t+1}\left(s_{0}\right)=f\left(V_{t}\left(s_{0}\right)+\lambda \alpha \delta_{t}^{Q}\right)$$

Thus far, the CQ model has been described in its original form. Aside from generalization, the value of any state‐action pair not encountered remains as is rather than being subject to decay or “forgetting” with potential for overfitting (Barraclough et al., 2004; Ito & Doya, 2009; Kato & Morita, 2016; Morita & Kato, 2014; Toyama et al., 2017, 2019). (There are intriguing parallels in the mathematics of value decay and counterfactual updating for nonencountered representations that remain to be investigated elsewhere.) In contrast to previous RL models, the GRL (or GQL) model introduced here additionally applies a common AVPE signal to learning of other state‐action pairs belonging to the same category as the current state. Presently, the two‐alternative forced choice allows for a straightforward model of discriminative action generalization, such that the nonchosen action *a′*_*t*_ receives an inverse value update as the complement of the chosen action *a*_*t*_ (where prime notation refers to complementarity here). The variables *a*_*L*_ and *a*_*R*_ stand for the left action and the right action, respectively:

$$a_{t}^{\prime }=\left\{\begin{array}{c} \begin{array}{cc} \;a_{R}, & a_{t}=a_{L} \end{array}\\ \begin{array}{cc} \;a_{L}, & a_{t}=a_{R} \end{array} \end{array}\right.$$

This counterfactual update is regulated by a negative parameter for the action‐generalization weight *g*_*A*_ (for −1 ≤ *g*_*A*_ ≤ 0) that modulates the original learning rate. Although associative action generalization is a possibility elsewhere, this parameter is not allowed to be positive here because the effective input to the choice function is the difference between two action values, rendering overgeneralization across actions essentially indistinguishable from a mere absence of learning. The constraint that absolute generalization weights do not exceed unity resolves the potential nonidentifiability issue of multiplied free parameters for generalized delta learning. More importantly, this constraint reflects the assumption—one shared with TD(*λ*)—that here generalized RPE signals would not be relayed with greater gain than the original RPE signal but rather lesser or equal gain. (In a different setting, this assumption might be relaxed under the appropriate circumstances.) As with state generalization, this equation is analogous to the previous one for the temporal generalization of the TD(*λ*) eligibility trace:

$$Q_{t+1}\left(s_{t},a_{t}^{\prime }\right)=f\left(Q_{t}\left(s_{t},a_{t}^{\prime }\right)+g_{A}\alpha \delta_{t}^{Q}\right)$$

Likewise, with only two states per category, state generalization entails an analogous formula where—in addition to the encountered state *s*_*t*_—the other, complementary state within the category *s*'_*t*_ receives a value update. The variables *s*_*A*_ and *s*_*B*_ refer to state A and state B (arbitrarily designated as such):

$$s_{t}^{\prime }=\left\{\begin{array}{c} \begin{array}{cc} \;s_{B}, & s_{t}=s_{A} \end{array}\\ \begin{array}{cc} \;s_{A}, & s_{t}=s_{B} \end{array} \end{array}\right.$$

This update is regulated by a state‐generalization weight *g*_*S*_ (for −1 ≤ *g*_*S*_ ≤ 1) that modulates the learning rate. Unlike overgeneralization across actions here, overgeneralization across states within a category can be detected. That is, the agent could incorrectly operate as if the category itself is a unitary state or at least partially conflate exemplars within a category. As the present paradigm is characterized by anticorrelational linkage between states within a category, a negative sign for *g*_*S*_ produces correct discriminative generalization, while a positive sign for *g*_*S*_ produces incorrect associative overgeneralization:

$$Q_{t+1}\left(s_{t}^{\prime },a_{t}\right)=f\left(Q_{t}\left(s_{t}^{\prime },a_{t}\right)+g_{S}\alpha \delta_{t}^{Q}\right)$$

As an intuitive constraint for the 7‐parameter model, the two factors of action generalization and state generalization interact multiplicatively to also update the complementary action for the complementary state. (This assumption in lieu of a third generalization parameter is also pragmatic in the interest of avoiding overfitting here, but it does not necessarily apply universally.) In the ideal case combining discriminative generalization across both dimensions (i.e., −1 ≤ *g*_*A*_ < 0 and −1 ≤ *g*_*S*_ < 0), this interactive state‐action generalization weight would appropriately be associative (0 < *g*_*S*_ *g*_*A*_ ≤ 1) for the one state‐action pair that is correlated with the original pair rather than anticorrelated:

$$Q_{t+1}\left(s_{t}^{\prime },a_{t}^{\prime }\right)=f\left(Q_{t}\left(s_{t}^{\prime },a_{t}^{\prime }\right)+g_{S}g_{A}\alpha \delta_{t}^{Q}\right)$$

Although the preceding constraint was hypothesized to be an appropriate one here, the possibility of an unconstrained interaction term as part of an 8‐parameter model was also considered and tested. Yet, in keeping with the initial constraint of only negative action generalization (i.e., −1 ≤ *g*_*A*_ ≤ 0), the partial constraint that the two updates for the complementary state could not share a common (nonzero) sign remained such that the interaction term was not determined by a wholly independent free parameter. Rather, a more general version of the preceding equation (from the nested case of *g*_*SA*_ = *g*_*A*_) includes the third factor of interactive state‐and‐action generalization *g*_*SA*_ (for −1 ≤ *g*_*SA*_ ≤ 0) as follows:

$$Q_{t+1}\left(s_{t}^{\prime },a_{t}^{\prime }\right)=f\left(Q_{t}\left(s_{t}^{\prime },a_{t}^{\prime }\right)+g_{S}g_{\mathrm{SA}}\alpha \delta_{t}^{Q}\right)$$

These learned action values serve as inputs to a probabilistic action‐selection policy *π*_*t*_ *(s,a)* characterized by the Boltzmann‐Gibbs softmax model as a discriminative (rather than generative) model of decision making (Luce, 1959; Shepard, 1957; Sutton & Barto, 1998). The approximation of a softmax (with perfect subtraction between two alternatives) does have limitations in accounting for decision‐making processes in an actual brain (Colas, 2017), but this component can suffice for the present purposes as a standard assumption for learning models. The choice function also includes inputs that simultaneously incorporate learning‐independent effects of action‐specific bias and hysteresis (Colas et al., 2017). For any interactive environment, including these terms is imperative—not only to account for additional variance but also to dissociate illusory mimicry of learning via sequential dependence from actual learning. That is, as learning promotes consistent repetition of responses within a state, so too can autocorrelational effects of hysteresis producing response repetition or alternation that coincidentally aligns with rotating states. (For example, perseveration offers a more parsimonious explanation for action repetition that could otherwise be attributed to an optimistic confirmation bias (Frank et al., 2004; Sharot, 2011; Sharot et al., 2011; Thorndike, 1932, 1933); in RL terms, the latter could translate to an asymmetry in learning rates favoring positive over negative outcomes (Cazé & van der Meer, 2013; Daw et al., 2002; Frank et al., 2007, 2009; Niv et al., 2012)—but at the cost of susceptibility to overfitting (relative to hysteresis) (Chambon et al., 2020; Gershman, 2016; Katahira, 2015, 2018; Palminteri, 2021; Sugawara & Katahira, 2021).) The baseline hysteresis model includes a dynamic perseveration (or alternation) bias *β*_*t*_ *(a)* (cf. Lau & Glimcher, 2005; Schönberg et al., 2007) as well as a constant lateral bias *β*_*R*_ with the arbitrary convention that rightward is positive. These internal biases complemented the learned external action values to dictate the policy's probabilities for each action via the following softmax function with temperature *τ* (for *τ* > 0), which regulates the stochasticity of choices reflecting noise as well as exploration against exploitation (Cohen et al., 2007; Daw et al., 2006; Gershman, 2018; Schulz & Gershman, 2019; Speekenbrink & Konstantinidis, 2015; Sutton & Barto, 1998; Thompson, 1933; Wilson et al., 2014). This equation reduces to a logistic function in the present two‐alternative forced‐choice task:

$$\pi_{t}\left(s_{t},a\right)=P\left(a_{t}=a\;|\;s_{t}\right)=\frac{\exp\{(Q_{t}\left(s_{t},a\right)+\beta_{t}\left(a\right)+\beta_{R}I_{R}\left(a\right))/\tau \}}{\sum_{a^{*}}\exp\{(Q_{t}\left(s_{t},a^{*}\right)+\beta_{t}\left(a^{*}\right)+\beta_{R}I_{R}\left(a^{*}\right))/\tau \}}$$

Modeling action hysteresis in terms of the dynamics of cumulative perseveration or alternation biases first requires an initialization of *β*_*t*_ *(a)*, which is here notated so as not to be confused with the parameter *β*_*0*_ described later:

$$\forall \;a:\beta_{t=0}\left(a\right)=0$$

A counter variable *N*_*t*_ is initialized at the beginning of each run to index the total number of actions performed within the run:

$$N_{0}=0$$

This action‐counter variable is simply incremented with each action performed successfully:

$$\forall \;a_{t}:N_{t}=N_{t-1}+1$$

Using this action index, the indicator function $I_{N_{t}}\left(a\right)$ tracks the action history across the run:

$$I_{N_{t}}\left(a\right)=\left\{\begin{array}{c} \begin{array}{cc} \;0, & a\neq a_{t} \end{array}\\ \begin{array}{cc} \;1, & a=a_{t} \end{array} \end{array}\right.$$

The exponentially decaying hysteretic bias is determined by its initial magnitude *β*_0_ and inverse decay rate *λ*_*β*_ (for 0 ≤ *λ*_*β*_ ≤ 1). A positive magnitude for such autocorrelation represents a perseveration bias in favor of repeating previous actions, whereas a negative magnitude represents an alternation bias in favor of switching between actions—that is, “antiperseveration”. The decay parameter is notated with the convention adopted for the eligibility trace, such that bases *λ* and *λ*_*β*_ both correspond to the complement of (i.e., unity minus) the exponential decay rate. The exponential decay of the bias proceeds with each action executed, as described in the following equation that integrates cumulative hysteretic biases:

$$\beta_{t+1}\left(a\right)=\sum_{i=0}^{N_{t}-1}\beta_{0}\lambda_{\beta }^{i}I_{N_{t}-i}\left(a\right)$$

The indicator function *I*_*R*_ *(a)* is used for a constant lateral bias with the arbitrary convention that a positive sign for *β*_*R*_ corresponds to a rightward bias while a negative sign corresponds to a leftward bias:

$$I_{R}\left(a\right)=\left\{\begin{array}{c} \begin{array}{cc} \;0, & a=a_{L} \end{array}\\ \begin{array}{cc} \;1, & a=a_{R} \end{array} \end{array}\right.$$

The final GRL model presently includes seven free parameters altogether—namely, learning rate *α*, action‐generalization weight *g*_*A*_, state‐generalization weight *g*_*S*_, softmax temperature *τ*, rightward (or leftward) bias *β*_*R*_, and initial magnitude *β*_*0*_ coupled with inverse decay rate *λ*_*β*_ for the exponential decay of the perseveration (or alternation) bias. For a paradigm such as this, the eligibility parameter *λ* cannot be tuned as a free parameter without a multistep Markov decision process (MDP) including intermediate states. As the time steps are discretized with a single step back per trial here, this element was fixed at *λ* = 0.5 by default for predicting dynamics of neural activity. This assignment, which did not substantially impact the results if changed, is also in agreement with previous fitted results (mean *λ* = 0.684) arrived at with a two‐step MDP and otherwise comparable methodology (Colas et al., 2017).

### 4.8 Computational modeling: Model‐based learning

Rather than the implicit model of task structure that emerges from the discriminative generalization of GRL, a cognitive map (i.e., model) could instead be represented explicitly as part of a proper model‐based algorithm. The following (cognitive) model‐based algorithms track a hierarchical metastate that corresponds to the generalizable structure within each state category (e.g., faces or houses). From this intuition, the possible hypotheses *h* for the binary metastate can be summarized as “AL/BR” or “AR/BL” for a given category *c* with complementarity between states and actions (Figure 1c), where “AR/BL” means state *s*_*A*_ rewards the right action *a*_*R*_ while state *s*_*B*_ rewards the left action *a*_*L*_. Whereas the model‐free learner naïvely initialized at zero, the model‐based learner initializes the estimated probabilities of the metastate hypotheses *P*_*t*_ *(h|c)* at 1/2 for a uniform prior within each category:

$$\forall \;\left(c,\left\{h\;|\;c\right\}\right):P_{0}\left(h\;|\;c\right)=\frac{1}{\left|\left\{h\;|\;c\right\}\right|}=\frac{1}{2}$$

For both types of model‐based systems that follow, the consistent hypothesis *ĥ(s*_*t*_ *,a*_*t*_ *,r*_*t+1*_ *)* for an observed state‐action‐outcome sequence is first inferred according to a binary rule. Yet the trial's consistent hypothesis *ĥ* (“*h*‐hat”) is not necessarily true to the block's actual metastate for the category because of the stochastic nature of the environment. In other words, this initial inference only functions as an intermediate input to either model‐based learning process:

$$\hat{h}\left(s_{t},a_{t},r_{t+1}\right)=\left\{\begin{array}{c} \begin{array}{cc} \;\left(\mathrm{AR},\mathrm{BL}\right), & \left|\begin{array}{c} \begin{array}{c} \;s_{t}=s_{A},a_{t}=a_{L},r_{t+1}=0\\ \;s_{t}=s_{A},a_{t}=a_{R},r_{t+1}=1 \end{array}\\ \begin{array}{c} \;s_{t}=s_{B},a_{t}=a_{L},r_{t+1}=1\\ \;s_{t}=s_{B},a_{t}=a_{R},r_{t+1}=0 \end{array} \end{array}\right. \end{array}\\ \begin{array}{cc} \;\left(\mathrm{AL},\mathrm{BR}\right), & \left|\begin{array}{c} \begin{array}{c} \;s_{t}=s_{A},a_{t}=a_{L},r_{t+1}=1\\ \;s_{t}=s_{A},a_{t}=a_{R},r_{t+1}=0 \end{array}\\ \begin{array}{c} \;s_{t}=s_{B},a_{t}=a_{L},r_{t+1}=0\\ \;s_{t}=s_{B},a_{t}=a_{R},r_{t+1}=1 \end{array} \end{array}\right. \end{array} \end{array}\right.$$

Given the two possibilities, the trial's alternative hypothesis *ĥ′(s*_*t*_ *,a*_*t*_ *,r*_*t+1*_ *)* is represented with the previous convention for prime notation such that “*h*‐hat‐prime” is complementary to “*h*‐hat”:

$${\hat{h}}^{\prime }\left(s_{t},a_{t},r_{t+1}\right)=\left\{\begin{array}{c} \begin{array}{cc} \;\left(\mathrm{AL},\mathrm{BR}\right), & \hat{h}\left(s_{t},a_{t},r_{t+1}\right)=\left(\mathrm{AR},\mathrm{BL}\right) \end{array}\\ \begin{array}{cc} \;\left(\mathrm{AR},\mathrm{BL}\right), & \hat{h}\left(s_{t},a_{t},r_{t+1}\right)=\left(\mathrm{AL},\mathrm{BR}\right) \end{array} \end{array}\right.$$

### 4.9 Computational modeling: State‐prediction error

The simpler model‐based algorithm operates with a heuristic analogous to the delta learning of model‐free RL but computes a state‐prediction error (SPE) *δ* ^SPE^_*t*_ rather than a reward‐prediction error (RPE). The SPE is essentially a generalized prediction error in its own right. Whereas for a multistep MDP the transition function for states and state‐action pairs (Bellman, 1957; Sutton & Barto, 1998) could be learned with another type of SPE as part of a dynamic‐programming algorithm (cf. Gläscher et al., 2010; Lee et al., 2014), this novel type of SPE is instead concerned with the generalizable metastate of the active state's category *c*_*t*_. Hence the SPE here is a “metastate‐prediction error” (MPE). Unlike the signed RPE, the unsigned SPE or MPE takes the difference between unity and the probability estimate for the state‐action‐outcome sequence's consistent hypothesis:

$$\delta_{t}^{\mathrm{SPE}}=1-P_{t}\left(\hat{h}\left(s_{t},a_{t},r_{t+1}\right)\;|\;c_{t}\right)$$

The update of the probability estimate is weighted by a model‐based learning rate *α*_*SPE*_ (for 0 ≤ *α*_*SPE*_ ≤ 1), which is again analogous to RL:

$$P_{t+1}\left(\hat{h}\left(s_{t},a_{t},r_{t+1}\right)\;|\;c_{t}\right)=P_{t}\left(\hat{h}\left(s_{t},a_{t},r_{t+1}\right)\;|\;c_{t}\right)+\alpha_{\mathrm{SPE}}\delta_{t}^{\mathrm{SPE}}$$

Moreover, the probability estimate for the trial's alternative hypothesis *ĥ′(s*_*t*_ *,a*_*t*_ *,r*_*t+1*_ *)* is proportionally decreased as well, thus fixing the sum of the probabilities to unity:

$$P_{t+1}\left({\hat{h}}^{\prime }\left(s_{t},a_{t},r_{t+1}\right)\;|\;c_{t}\right)=P_{t}\left({\hat{h}}^{\prime }\left(s_{t},a_{t},r_{t+1}\right)\;|\;c_{t}\right)-\alpha_{\mathrm{SPE}}P_{t}\left({\hat{h}}^{\prime }\left(s_{t},a_{t},r_{t+1}\right)\;|\;c_{t}\right)$$

### 4.10 Computational modeling: Hidden Markov model

The more complex model‐based algorithm utilizes Bayesian optimization while specifying an even more explicit and complete model of the exploitable structure in this environment. Whereas previous implementations of the hidden Markov model (HMM) (Ghahramani, 2001) have emphasized reversals between linked states or actions (as a Markov process) (cf. Aquino et al., 2020; Hampton et al., 2006; Prévost et al., 2013), the hidden state in this HMM uniquely corresponds to the hierarchical metastate of a category subsuming active states—that is, a “hidden metastate”. The likelihood function for an outcome given the preceding state‐action pair and an assumed hypothesis *P(r*_*t+1*_ *|h,(s*_*t*_ *,a*_*t*_ *))* is determined by a consistency parameter *θ*_*0*_ (for ½ ≤ *θ*_*0*_ ≤ 1) for a binary distribution, representing the agent's belief about the consistency of the rule for a given metastate's probabilistic outcomes:

$$P^{\text{Likelihood}}(r_{t+1}|\;\;h,\left(s_{t},a_{t}\right))=\left\{\begin{array}{cc} \;\theta_{0}, & h=\hat{h}\left(s_{t},a_{t},r_{t+1}\right)\\ \;1-\theta_{0}, & \;h={\hat{h}}^{\prime }\left(s_{t},a_{t},r_{t+1}\right) \end{array}\right.$$

An optimal Bayesian learner tasked with reversal learning such as this can employ belief propagation (Jordan, 1998), such that knowledge of reversing contingencies is directly factored into the integration of changing evidence. For the full HMM, a second fitted parameter represented the reversal rate *θ*_*1*_ (for 0 ≤ *θ*_*1*_ ≤ 1) applied to either complementary hypothesis *h*′, but the reduced HMM0 variant omits this parameter (*θ*_*1*_ = 0) so as to not represent any specific expectation of metastate reversals. By the onset of a new trial, the preceding posterior forms the new prior with an update determined by this baseline reversal rate:

$$P_{t}^{\text{Prior}}\left(h\;|\;c_{t}\right)=\theta_{1}P_{t-1}^{\text{Posterior}}\left(h^{\prime }\;|\;c_{t}\right)+\left(1-\theta_{1}\right)P_{t-1}^{\text{Posterior}}\left(h\;|\;c_{t}\right)$$

Following Bayes' rule, the updated posterior upon experiencing a new state‐action‐outcome sequence integrates prior knowledge with the likelihood of the observation given a hypothesis for the category's metastate:

$$P_{t+1}^{\text{Posterior}}\left(h\;|\;c_{t}\right)=\frac{P^{\text{Likelihood}}(r_{t+1}\;|\;h,\left(s_{t},a_{t}\right))P_{t}^{\text{Prior}}\left(h\;|\;c_{t}\right)}{\sum_{h^{*}}P^{\text{Likelihood}}(r_{t+1}\;|\;h^{*},\left(s_{t},a_{t}\right))P_{t}^{\text{Prior}}\left(h^{*}\;|\;c_{t}\right)}$$

### 4.11 Computational modeling: Model‐based value

Unlike RL and GRL, the model‐based algorithms just described do not learn about value per se; rather, these algorithms track the probabilities of hypothesized metastates and with inference translate these to subjective value in an additional layer of computation. As an alternative to a cached value estimate, the model‐based action value *Q* ^*MB*^_*t*_ *(c,s,a)* is inferred for the hierarchy of a category‐state‐action triplet from probability estimates for the category's metastate. Note that, as an input to model‐based decision making in this setting, this action‐value estimate is not equivalent to the action's expected value for the actual reward yield of the outcome *E*_*t*_ *[r*_*t+1*_ *|c*_*t*_ *,s*_*t*_ *,a]*. For the Bayesian HMM, that expectation also factors in the likelihood function with beliefs about rule consistency:

$$E_{t}\left[r_{t+1}\;|\;c_{t},s_{t},a\right]=\sum_{h}\sum_{r}P^{\text{Likelihood}}(r\;|\;h,\left(s_{t},a\right))P_{t}^{\text{Prior}}\left(h\;|\;c_{t}\right)r$$

Although analogous to the cached reward prediction of RL, the preceding expected value would instead be computed on the fly by the HMM. However, this expectation was not the relevant input to the action‐selection policy. Rather, the HMM follows the SPE in more efficiently choosing according to the estimated probability that an action's congruent hypothesis for the category and state is correct. This feature is optimal for the HMM here and was not just implemented in the interest of control in model comparison: For the complementary hypotheses of this paradigm, it proportionately amplifies the difference between action values so as to create greater opportunity for greedy exploitation of presumed knowledge while still achieving exploration through counterfactual learning. Hence the model‐based action value is yoked to the dynamic beliefs of either algorithm with a shared equation more directly translating the probability estimates for the metastate hypotheses:

$$Q_{t}^{\mathrm{MB}}\left(c,s,a\right)=\sum_{r}P_{t}\left(\hat{h}\left(s,a,r\right)\;|\;c\right)r=\left\{\begin{array}{c} \begin{array}{c} \begin{array}{cc} \;P_{t}(\left(\mathrm{AL},\mathrm{BR}\right)\;|\;c), & s=s_{A},a=a_{L} \end{array}\\ \begin{array}{cc} \;P_{t}(\left(\mathrm{AR},\mathrm{BL}\right)\;|\;c), & s=s_{A},a=a_{R} \end{array} \end{array}\\ \begin{array}{c} \begin{array}{cc} \;P_{t}(\left(\mathrm{AR},\mathrm{BL}\right)\;|\;c), & s=s_{B},a=a_{L} \end{array}\\ \begin{array}{cc} \;P_{t}(\left(\mathrm{AL},\mathrm{BR}\right)\;|\;c), & s=s_{B},a=a_{R} \end{array} \end{array} \end{array}\right.$$

### 4.12 Computational modeling: Dual systems

The different model‐based models and basic RL were all nested within dual‐systems models that combine model‐based and model‐free techniques in parallel. The action‐selection policy can thus be expanded with another parameter as the model‐based weight *w*_*MB*_ (for 0 ≤ *w*_*MB*_ ≤ 1), which modulates the weight of either model‐based system's estimate of action value. Model‐based weighting can reflect the fidelity of the model‐based system as well as the arbitrating agent's confidence in its reliability (Daw et al., 2011; Gläscher et al., 2010; Lee et al., 2014). The nested cases of *w*_*MB*_ = 0 and *w*_*MB*_ = 1 correspond to purely model‐free and purely model‐based agents, respectively. Whereas a model‐free system—including GRL despite linked representations—caches value for only state‐action pairs, inferential model‐based value explicitly factors in the hierarchical metastates characterizing category‐state‐action triplets:

$$\pi_{t}\left(c_{t},s_{t},a\right)=\frac{\exp\left\{\left(w_{\mathrm{MB}}Q_{t}^{\mathrm{MB}}\left(c_{t},s_{t},a\right)+\left(1-w_{\mathrm{MB}}\right)Q_{t}\left(s_{t},a\right)+\beta_{t}\left(a\right)+\beta_{R}I_{R}\left(a\right)\right)/\tau \right\}}{\sum_{a^{*}}\exp\left\{\left(w_{\mathrm{MB}}Q_{t}^{\mathrm{MB}}\left(c_{t},s_{t},a^{*}\right)+\left(1-w_{\mathrm{MB}}\right)Q_{t}\left(s_{t},a^{*}\right)+\beta_{t}\left(a^{*}\right)+\beta_{R}I_{R}\left(a^{*}\right)\right)/\tau \right\}}$$

### 4.13 Model fitting

A total of 17 learning models were tested against each other and the hysteresis model (*α* = *g*_*A*_ = *g*_*S*_ = 0) (Table 2). A subset of 11 models corresponded to a factorial model comparison including every nested permutation with respect to the two dimensions of generalization in GRL—to wit, no generalization (*g*_*A*_ = *g*_*S*_ = 0), maximally optimal discriminative action generalization (*g*_*A*_ = −1, *g*_*S*_ = 0), free action generalization (−1 ≤ *g*_*A*_ ≤ 0, *g*_*S*_ = 0), maximally suboptimal associative state generalization (*g*_*A*_ = 0, *g*_*S*_ = 1), maximally optimal discriminative state generalization (*g*_*A*_ = 0, *g*_*S*_ = −1), free state generalization (*g*_*A*_ = 0, −1 ≤ *g*_*S*_ ≤ 1), maximally optimal discriminative action generalization and maximally suboptimal associative state generalization (*g*_*A*_ = −1, *g*_*S*_ = 1), maximally optimal discriminative action and state generalization (*g*_*A*_ = *g*_*S*_ = −1), free action and state generalization with a shared parameter (*g*_*A*_ = min{0, *g*_*S*_}, −1 ≤ *g*_*S*_ ≤ 1), free action generalization plus free state generalization with dual parameters (−1 ≤ *g*_*A*_ ≤ 0, −1 ≤ *g*_*S*_ ≤ 1), and free interaction between state and action generalization (*g*_*SA*_ ≠ *g*_*A*_). Competing degenerate models thus benefited from having fewer degrees of freedom to penalize.

The (cognitive) model‐based models were tested similarly alongside the others. The SPE model was nested within a dual‐systems “SPE+RL” model. Nested within the full HMM was the HMM0 variant without explicit reversals of the hidden state (i.e., metastate) (*θ*_*1*_ = 0). These two models were nested within their respective dual‐systems models that included RL in parallel—that is, “HMM0+RL” and “HMM+RL”. The instantiation of the GRL model optimally tuned with *g*_*A*_ = *g*_*S*_ = −1 is also of special note for serving as a model‐free approximation (cf. Gläscher et al., 2009; Hampton et al., 2007) of a model‐based scheme for an idealized optimal agent in an environment with perfectly anticorrelated states and actions. (Under different circumstances elsewhere, differing degrees of generalization in proportion to the statistics of another environment could be better suited to partially or dynamically correlated or anticorrelated states and actions.) As the most interpretable model comparison is one grounded in a factorial design with systematic testing of parameters, emphasis is due for commensurable models within a single class such as RL (including GRL in this context).

In capturing action‐specific bias and hysteresis, the 4‐parameter hysteresis model offers a nested null model that is more viable as a control than a zero‐parameter chance model with random choices or even an intercept model, which has only one parameter for the probability of an arbitrary action *P(A*_*1*_ *)*. Thus, sensitivity to learnable outcomes or lack thereof can be detected with greater precision by setting the fitting performance of the hysteresis model as a benchmark for comparison with candidate models that feature relevant learning; a participant could then be set aside in the Nonlearner group for demonstrating a lack of reward sensitivity across all learning models. (In the absence of learning, inclusion of *τ* is redundant in practice but nevertheless maintained as a degree of freedom because of its conceptual relevance as the stochasticity parameter as opposed to a learning parameter per se.)

This uniquely comprehensive modeling approach—that is, the foundation of a 5‐parameter model (Colas et al., 2017) rather than the standard 2‐parameter model with only learning rate and temperature—also aims to enhance parameter identifiability with respect to actual learning as opposed to other sources of variance that may obscure or mimic learning (Lau & Glimcher, 2005; Schönberg et al., 2007). Whereas alternative solutions find recourse in regularization via fully group‐level estimation (i.e., concatenating data sets or averaging parameters) or the intermediate approach of hierarchical Bayesian modeling across individuals (Ahn et al., 2017; Daw, 2011; Gershman, 2016), the present solution of a more complete yet parsimonious model—in this case accounting for action‐specific bias and hysteresis—avoids compromising the independence of separate data sets. As per the bias‐variance tradeoff, even reducing variance with the constraints of hierarchical group‐level estimation would necessarily introduce bias both toward the average across individuals and toward the specifications of a parametric probability distribution. In other words, the present technique cannot be diminished by potentially inappropriate assumptions that a given participant is learning and furthermore learning in a particular way merely because other participants in the aggregate have mostly demonstrated learning and an overall tendency to learn in a particular way. Added complexities such as idiosyncratic strategies for generalization impose even greater demands for accommodating individual differences. This subject‐level interpretability also extends to (computational) model‐based analysis of neurophysiological data (O'Doherty et al., 2007), where advantages can include more precise estimation of signal dynamics and parameters of interest—including between‐subject analyses—as well as the capacity to classify distinct types of performance in subgroup analyses—for example, learners versus nonlearners (Colas et al., 2017) or associative generalizers versus discriminative generalizers.

The competing models were all fitted to empirical behavior at the level of individual subjects via maximum‐likelihood estimation. Free parameters were optimized for overall goodness of fit to a subject's sequence of actions with randomly seeded iterations of the Nelder‐Mead simplex algorithm (Nelder & Mead, 1965). All modeling and fitting procedures were programmed with MATLAB. The Akaike information criterion with correction for finite sample size (AICc) (Akaike, 1974; Hurvich & Tsai, 1989) provided a means to adjust for model complexity when comparing models that differ in degrees of freedom. The preferred model was also to provide the basis for the subsequent neuroimaging analysis.

To verify the discriminability of the preferred 7‐parameter GRL model, each fitted instantiation of the model was subsequently used to simulate a data set yoked to that of the respective subject. Another complete model comparison was conducted for these simulated data as a test of model recovery that would indicate whether this model could be discriminated reliably among the competing alternatives. The same procedure was repeated with simulations conversely derived from 5‐parameter basic RL for additional reassurance that the original results could not be reduced to mere overfitting.

### 4.14 Data analysis: Behavior

Performance on the learning task was assessed for each participant by calculating overall accuracy as the proportion of choices of the option that could result in delivery of a reward, excluding choices made for initial encounters with novel cues. Accuracy was compared with the chance level of 50% for each participant using a one‐tailed binomial test. A subset of participants was initially set aside as the “Good learner” group if the accuracy score was significantly greater than the chance level (Schönberg et al., 2007); subsequent modeling could also confirm that this label was appropriate for each individual within the group. The remaining participants with accuracy not significantly greater than chance were subsequently assigned to either the “Poor learner” group or the “Nonlearner” group according to whether or not a learning model could yield a significant improvement in goodness of fit relative to a hysteresis model without sensitivity to actual outcomes (Colas et al., 2017). Reaction time (RT) was also compared between these primary groups via one‐tailed independent‐samples *t* tests hypothesizing a speed‐accuracy tradeoff.

Across the two learner groups, the second stage of model comparison reclassified these individuals in secondary “Discriminative generalizer”, “Nongeneralizer”, and “Associative generalizer” groups for the cases of *g*_*S*_ < 0, *g*_*S*_ = 0, and *g*_*S*_ > 0, respectively, as determined by the individually fitted GRL model. A subset of diagnostic trials were selected to represent the first opportunities for generalization of reward within each block. Sixteen trials in total corresponded to the two categories each having two newly rewarded actions per each of four runs (i.e., 2 × 2 × 4). First‐generalization accuracy was compared against chance with one‐tailed one‐sample *t* tests within each model‐defined group; Discriminative generalizers were hypothesized to perform above chance, whereas Nongeneralizers and especially Associative generalizers were hypothesized to perform below chance in the absence of direct reinforcement for the new reward contingencies. One‐tailed independent‐samples *t* tests followed to verify the presumed ranking of Discriminative generalizers, Nongeneralizers, then Associative generalizers. Moreover, a correlation between the GRL model's fitted parameter and first‐generalization accuracy was tested for using linear regression with a one‐tailed one‐sample *t* test and the Pearson correlation coefficient. For a posterior predictive check of each generative model with respect to these results, simulated data sets were yoked to the empirical data sets and analyzed in the same fashion after averaging across 1,000 simulations.

Taking the free parameters fitted for each subject, the overall reward sensitivity of each instantiation of the GRL model was quantified as *log(α(1*−*g*_*A*_−*g*_*S*_ *+g*_*S*_ *g*_*A*_ *)/τ)* (cf. Colas et al., 2017; Schönberg et al., 2007) with a logarithmic transformation for more interpretable rescaling prior to presentation of the results. Relative to the softmax temperature *τ*, this formula factors in the magnitudes of all four possible updates of action values with each duplicated and relayed RPE signal—that is, for the current (*α*) and complementary (*g*_*A*_ *α*) actions within the current state as well as the current (*g*_*S*_ *α*) and complementary (*g*_*S*_ *g*_*A*_ *α*) actions within the complementary state. Considering that fitted RL models are typically characterized by a correlation between learning rate and softmax temperature that reflects elongated maxima in their joint likelihood function (Daw, 2011), this sensitivity ratio is a more precise and more relevant measure of a learning model's sensitivity than either the learning rate or the temperature alone. Such an alpha‐tau correlation was observed across Learner groups in both data sets (3FH: *r* = 0.518, *t*_*38*_ = 3.73, *p* < 10^−3^; 7CM: *r* = 0.427, *t*_*19*_ = 2.05, *p* = .027). Accordingly, sensitivity was compared between the Good‐learner and Poor‐learner groups by way of a one‐tailed independent‐samples *t* test. Post‐hoc one‐tailed independent‐samples *t* tests were subsequently conducted for action generalization and state generalization. To test for correlations between sensitivity and accuracy or RT, between action generalization and accuracy or RT, between state generalization and accuracy or RT, and between action generalization and state generalization, linear regression was performed with one‐tailed one‐sample *t* tests and reported with the Pearson correlation coefficient.

Across all trials, analyses of choice data based on preceding outcomes first separated the most recent trials in which either the same (i.e., current) state was encountered or the other, complementary state within the current category was encountered. These trials were further binned according to whether the trial rewarded a given action or provided no such reward. The probability of repeating the prior trial's action was calculated within each of four bins: “same/reward”, “same/no‐reward”, “other/reward”, and “other/no‐reward”. Given the complementarity of states within a category to facilitate discriminative state generalization by design, the hypothesis for repeating actions from the “other” state was an inversion of the hypothesis for the “same” state: A previous reward in the same state was supposed to increase repetition of the action, whereas a previous reward in the other state was supposed to decrease repetition. For each participant group, one‐tailed one‐sample *t* tests compared the probability of repeating the respective state's last action between the “reward” and “no‐reward” conditions either within same‐state trials or within other‐state trials. Moreover, another set of one‐tailed one‐sample *t* tests assessed the between‐state interaction of the effect between the “reward” and “no‐reward” conditions. To verify that the GRL model could quantitatively reproduce these results as well, simulated data sets yoked to the empirical data sets were analyzed in the same manner for a second posterior predictive check.

As computational modeling provided quantitative trial‐by‐trial estimates of action‐value representations, these dynamic variables could in turn be related to psychometric functions for choices and RTs. A logistic‐regression model first modeled the probability of repeating the most recent action (independent of state) as a function of the normalized difference between action values. A linear‐regression model likewise modeled the RT as a function of the normalized absolute value of the difference between action values. In order to accommodate intersubject variability in the range of estimated values, differences in action values were normalized with respect to the maximum absolute value for each subject. Parameters for these mixed‐effects models were first estimated at the level of individual subjects and subsequently assessed within each subject group using one‐tailed one‐sample *t* tests.

### 4.15 Data analysis: Neuroimaging

Analysis of the fMRI data was conducted with SPM and carried out identically within each of the 3‐T and 7‐T data sets. This (computational) model‐based analysis (O'Doherty et al., 2007) was grounded in the explicit quantitative dynamics predicted by the GRL model with subject‐specific parameters (Figure 7). The GLM of BOLD signals was essentially a tripartite model characterized by parametric regressors for value, RPE, and RT. For a paradigm such as this with a single‐step cue‐outcome sequence, disambiguating all three types of signals is nontrivial (as alluded to previously with reference to the CQ model).

Indicator variables modeled as boxcar functions described all of the events within the sequence of each trial. These indicators included decision time (with variable duration), face (or color) cues (with a duration of 2 s), house (or motion) cues (2 s), left‐hand responses (2 s), right‐hand responses (2 s), the ISI (3 s), outcomes (1 s), and the ITI (3–7 s). In the case of a missed trial marked by failure to respond in the 2‐s window, events were coded as separate indicator variables for face (or color) cues (2 s), house (or motion) cues (2 s), error feedback (1 s), late left‐hand responses within a 1‐s window (1 s), late right‐hand responses within a 1‐s window (1 s), and the ITI (6–10 s). In preventing the complications of temporal prediction‐error signals such as in TDRL (McClure et al., 2003; O'Doherty et al., 2003; O'Doherty et al., 2004; Sutton, 1988; Sutton & Barto, 1998), the fixed ISI was sufficient and did not result in rank deficiency for the design matrix because of not only jitter in the ITI but also the quantitative precision of narrowly specified (computational) model‐based regressors.

The RT regressor was specified as a boxcar function aligned with cue onset and extending with a variable duration corresponding to trial‐by‐trial RT. Value signals were continuous and included the state value *V*_*t*_ *(s*_*0*_ *)* of the preparatory state, the chosen action value *Q*_*t*_ *(s*_*t*_ *,a*_*t*_ *)* for the active state, and the value of the outcome state. Similarly, learning signals in the form of RPE signals included both the SVPE *δ* ^*V*^_*t*_ computed upon encountering the cue—as per the TD algorithm—and the AVPE *δ* ^*Q*^_*t*_ computed upon encountering the outcome. Value and learning signals were modeled as parametric modulators of boxcar functions, and the duration of each boxcar function corresponded to the duration of the respective stimulus with one exception: Value signals were assumed to persist beyond stimulus offset through the subsequent ISI. The reason for this convention is that the expectation for value should remain the same with negligible temporal discounting. Although the distinctions between state value and action value—or between the SVPE and the AVPE—are important in general (Averbeck & O'Doherty, 2022; Colas et al., 2017; O'Doherty et al., 2004), such distinctions are beyond the scope of the present study and were necessarily omitted here in consideration of the single‐step cue‐outcome sequence, which challenges dissociability.

As orthogonalization was forgone to avoid potential distortions of the parameter estimates or their interpretation (Mumford et al., 2015), the complete predictions of this TDRL model were taken advantage of to minimize inevitable multicollinearity (Colas et al., 2017; cf. Behrens et al., 2008; Zhang et al., 2020). In addition to effects of value on RT (Busemeyer & Townsend, 1993; Colas, 2017; Laming, 1968; Luce, 1986; Ratcliff, 1978; Usher & McClelland, 2001), there is also a relation between value and the RPE; the latter is a linear combination (i.e., subtraction) of outcome value and estimated value. By collapsing events across a trial into unitary regressors for value and learning signals, the correlation between value and the RPE could be mitigated to a tractable level of dissociability (3FH: mean *r* ^*2*^ = 0.431 across subjects; 7CM: mean *r* ^*2*^ = 0.389). Dissociation was also achieved between value and RT (3FH: mean *r* ^*2*^ = 0.008; 7CM: mean *r* ^*2*^ = 0.007) as well as between the RPE and RT (3FH: mean *r* ^*2*^ = 0.014; 7CM: mean *r* ^*2*^ = 0.021), such that triply dissociated value, decision, and learning signals could all be accounted for in parallel.

All of the aforementioned predictor variables were convolved with a canonical double‐gamma hemodynamic‐response function as inputs to the GLM. The design matrix also included the confound regressors without convolution—to wit, motion parameters, CSF signal, white‐matter signal, global signal, framewise displacement, standardized DVARS, indicators for nonsteady states (i.e., outlier volumes), DCT basis functions, tCompCor components, aCompCor components, ICA‐AROMA noise components, and RETROICOR components from cardiac and respiratory data. Also among the nonconvolved regressors were a first‐degree autoregressive (i.e., “AR(1)”) term and a constant term. GLMs were first estimated at the level of an individual subject, and contrasts of parameter estimates were subsequently computed for the parametric regressors at the group level as part of a mixed‐effects analysis. The groups corresponded to Good learners, Poor learners, Nonlearners, or all learners (including both Good and Poor learners). Positive effects of these contrasts were tested for using one‐tailed one‐sample *t* tests.

Strictly aiming for the rigor of quantitative parametric regressors, the default thresholds for statistical significance and cluster extent were preset at standard levels of *p* < .005 and *k* ≥ 10 voxels (Forman et al., 1995; Lieberman & Cunningham, 2009). Whereas whole‐brain correction for multiple comparisons was precluded by so many voxels being sampled with high resolution—and especially so at 7 T—precise regions of interest (ROIs) could constrain the hypothesis space a priori with established precedents for the neural correlates of evaluation and value‐based decision making and learning. Spherical coordinate‐based ROIs with 6‐mm radii were applied to small‐volume correction (SVC) controlling for the familywise error rate (FWE) at *p* < .05. A given set of ROIs was first tested as a network; post‐hoc tests followed for individual ROIs within the set. For visualization, statistical‐parametric maps were overlaid on averages of processed anatomical images from the respective participants included in a given analysis.

For learning signals, a prior high‐resolution study with comparable methodology (Colas et al., 2017) provided focal coordinates for variants of the RPE signal in the left anterior caudate nucleus at (−8, 18, −8) or (−8.4, 18, −8.4) for 3‐T or 7‐T images, respectively; the right nucleus accumbens at (8, 12, −4) or (8.4, 12, −3.6); the right ventral putamen at (18, 12, −12); the left nucleus accumbens at (−12, 10, −6) or (−12, 9.6, −6); the right dorsal putamen at (28, 6, 0) or (27.6, 6, 0); the left dorsal caudate nucleus at (−18, 2, 16) or (−18, 2.4, 15.6); and the left SN at (−10, −14, −12) or (−9.6, −14.4, −12). More broadly, exploratory anatomical ROIs to be searched for uncorrected results included the entire striatum and the dopaminergic midbrain, comprising the SN and the ventral tegmental area.

For value signals, a pair of meta‐analytic studies with largely compatible results (Bartra et al., 2013; Clithero & Rangel, 2014) provided coordinates for the correlates of monetary value in bilateral ventromedial prefrontal cortex (vmPFC) at (0, 46, −8) or (0, 45.6, −8.4) for 3‐T or 7‐T images, respectively; the right nucleus accumbens at (10, 16, −6) or (9.6, 15.6, −6); the left nucleus accumbens at (−10, 10, −6) or (−9.6, 9.6, −6); and bilateral posterior cingulate cortex (PCC) at (−2, −34, 38) or (−2.4, −33.6, 38.4). Coordinates were averaged between the two meta‐analyses, which identified a common set of regions for the pertinent contrast. Exploratory ROIs to be searched for uncorrected results included the entirety of vmPFC, the striatum, and PCC.

For decision signals, a meta‐analysis (Yarkoni et al., 2009) provided coordinates for the correlates of RT across different tasks in bilateral medial frontal cortex (MFC) at (0, 12, 48). Encompassing the vicinity of the supplementary motor area (SMA), the pre‐SMA, and dorsal anterior cingulate cortex, MFC as a whole also served as an exploratory ROI to be searched for uncorrected results. Although other brain areas such as premotor cortex and posterior parietal cortex have been implicated in decision‐making processes as well (Cisek, 2012; Cisek & Kalaska, 2010; Gold & Shadlen, 2007), greater effector‐specific lateralization in these regions limits their interpretability with respect to the more abstract value‐based decision making sought here. In any case, the scope of the present study is limited such that the gamut of diverse decision‐making signals is not investigated in the fullest detail.

Regressors for specific effects of state generalization and action generalization were quantified as *−g*_S_ */τ* and *−g*_A_ */τ*, respectively, to test for interactions with RPE signals at the second level between subjects. The ROIs applied to the original RPE contrast were utilized here as well. However, the paradigm of the study that these ROIs were derived from (Colas et al., 2017) did not include the present factor of generalization in any form. As such, the hypotheses motivating these ROIs in their original context were less definitive for exploration in this new context. On the other hand, the ROIs still can serve as candidates for first‐pass investigation. Lacking proper precedent, exploratory investigation throughout the striatum and especially the dopaminergic midbrain was considered more openly here.

Additionally for effects of generalization, the most active locus within the hippocampus was extracted from broad meta‐analytic results in the Neurosynth database (Yarkoni et al., 2011). Across results including the term “hippocampus” in the report's abstract, the peak activations derived from uniformity and association tests coincided at (−28, −18, −16), which was reflected across hemispheres with bilateral SVC as (±28, −18, −16) or (±27.6, −18, −15.6) for 3‐T or 7‐T images, respectively. Another exploratory ROI further included the entire hippocampal region.

With regard to state generalization, presenting categorical stimuli elicited activation in the expected cortical regions: The categories of faces, houses, colors, and directions of motion activated the FFA (Kanwisher et al., 1997), the PPA (Epstein & Kanwisher, 1998), color‐sensitive visual area V4 (or V8) (Beauchamp et al., 1999; Hadjikhani et al., 1998; Wade et al., 2002; Zeki et al., 1991), and the motion‐sensitive middle‐temporal area MT (or V5) (Tootell et al., 1995; Watson et al., 1993; Zeki et al., 1991), respectively (*p* < .005). With regard to action generalization, actions executed with the left and right hands generated the expected activation in contralateral motor cortex and ipsilateral cerebellar cortex (Grafton et al., 1992) at both 3 T and 7 T (*p* < .005). In the interest of being concise, results for these less critical contrasts are not reported in further detail.

As a control analysis, a regressor for the normalized learning rate was quantified as *α/τ* to test for interactions with RPE signals at the second level. This contrast was juxtaposed with the generalization contrasts for the same sets of ROIs—the goal being to determine if the findings for generalization specifically were possibly confounded with a nonspecific effect of learning performance.

## Supporting information


**Table S1.** Model comparison: 3‐T Face/House version (Good‐learner group). Listed first for 3 nonlearning models and 17 learning models fitted to empirical data are absolute scores for deviance and the corrected Akaike information criterion (AICc) (where a lower score is better). These absolute scores were translated to residual goodness of fit relative to the hysteresis model (where a higher score is better). Winning results determined by the AICc are highlighted with boldface and italics. “df” stands for degrees of freedom. This table is related to Figure 3. The conventions for displaying this table also apply for Tables S2–S15.
**Table S2**. Model comparison: 3‐T Face/House version (Poor‐learner group). This table is related to Figure 3.
**Table S3**. Model comparison: 3‐T Face/House version (Nonlearner group). Nonlearners were defined as such in cases where the hysteresis model provided the best fit post‐correction.
**Table S4**. Model comparison: 7‐T Color/Motion version (Good‐learner group). This table is related to Figure 4.
**Table S5**. Model comparison: 7‐T Color/Motion version (Poor‐learner group). This table is related to Figure 4.
**Figure S1**. Discriminability of the GRL model: 3‐T Face/House version. Compare to Figure 3. Each fitted instantiation of the 7‐parameter “generalized reinforcement learning” (GRL) model (“AX|SY”) was used to simulate a data set yoked to that of the respective subject. Replications of the results from the original model comparison were achieved with these simulations as a demonstration of the discriminability of this preferred model with its additional degrees of freedom. This figure is related to Tables S6–S8.
**Table S6**. Discriminability of the GRL model: 3‐T Face/House version (Good‐learner group). This table is related to Figure S1.
**Table S7**. Discriminability of the GRL model: 3‐T Face/House version (Poor‐learner group). This table is related to Figure S1.
**Table S8**. Discriminability of the GRL model: 3‐T Face/House version (Nonlearner group). Compare to Table S3. The hysteresis model also provided the best fit for Nonlearners in silico.
**Figure S2**. Discriminability of the GRL model: 7‐T Color/Motion version. Compare to Figures 4 and S1. This figure is related to Tables S9 and S10.
**Table S9**. Discriminability of the GRL model: 7‐T Color/Motion version (Good‐learner group). This table is related to Figure S2.
**Table S10**. Discriminability of the GRL model: 7‐T Color/Motion version (Poor‐learner group). This table is related to Figure S2.
**Figure S3**. Discriminability of the basic RL model: 3‐T Face/House version. Compare to Figure S1. The basic RL model was recovered in lieu of the GRL model when substituting data simulated with basic RL. This converse model recovery again demonstrates an absence of overfitting. This figure is related to Tables S11–S13.
**Table S11**. Discriminability of the basic RL model: 3‐T Face/House version (Good‐learner group). This table is related to Figure S3.
**Table S12**. Discriminability of the basic RL model: 3‐T Face/House version (Poor‐learner group). This table is related to Figure S3.
**Table S13**. Discriminability of the basic RL model: 3‐T Face/House version (Nonlearner group). Compare to Tables S3 and S8. The hysteresis model also provided the best fit for Nonlearners in silico.
**Figure S4**. Discriminability of the basic RL model: 7‐T Color/Motion version. Compare to Figures S2 and S3. This figure is related to Tables S14 and S15.
**Table S14**. Discriminability of the basic RL model: 7‐T Color/Motion version (Good‐learner group). This table is related to Figure S4.
**Table S15**. Discriminability of the basic RL model: 7‐T Color/Motion version (Poor‐learner group). This table is related to Figure S4.
**Figure S5**. Predictions of the basic RL model. Compare to Figure 7. Representative dynamics generated by the basic RL model (*g*_*A*_ = *g*_*S*_ = 0) are shown for the same participant. Parameters were assigned as follows for this participant: *α* = 0.724, *λ* = 0.500, *τ* = 0.567, *β*_0_ = −0.084, *λ*_*β*_ = 0.715, and *β*_*R*_ = 0.252. Unlike GRL, basic RL updates the value of only the state‐action pair experienced on a given trial.
**Table S16**. Neural substrates of the RL framework: 3‐T Face/House version. Listed for every significant cluster (*p* < 0.005, *k* ≥ 10) are anatomical regions; hemispheres (“H”) as left (“L”), right (“R”), or bilateral (“B”); stereotactic coordinates in MNI space in mm (*x*, *y*, *z*); test statistics (*t*_*df*_); probability values (*p*); cluster extents in voxels (*k*); and results of small‐volume correction (SVC) at the cluster level (“C”) or the peak level (“P”) (*p*_*FWE*_ < 0.05), where marginally significant (“c” or “p” in lower case) (0.05 < *p*_*FWE*_ < 0.10) or uncorrected (“U”) (*p* < 0.005) results are also listed if the most stringent threshold for SVC was not attained within the region of interest. All relevant groupings of participants are included. The conventions for displaying this table also apply for Tables S17, S19, S20, S23, and S24. This table is related to Figure 8 and Table S18.
**Figure S6**. Neural substrates of the RL framework: 7‐T Color/Motion version (Dopaminergic midbrain). At 7 T, reward‐prediction error (RPE) signals from the GRL model were further localized to the substantia nigra (SN) (*p* < 0.005). This figure is related to Figure 9 and Tables S17 and S18.
**Table S17**. Neural substrates of the RL framework: 7‐T Color/Motion version. This table is related to Figures 9 and S6 and Table S18.
**Table S18**. Neural substrates of the RL framework: Summary. The first portion of fMRI analyses across data sets and participant groups (i.e., “All”, “Good”, and “Poor” learners) are summarized for the RL framework that serves as the foundation of the GRL model. Regions of interest (ROIs) were informed by prior studies modeling the reward‐prediction error, value, and reaction time. Initially, broader exploratory ROIs were defined anatomically and tested for uncorrected results (“U”) (*p* < 0.005). For RPE and value signals, coordinate‐based ROIs were first tested collectively via SVC at the set level (“S”) (*p*_*FWE*_ < 0.05). Post‐hoc tests followed for individual ROIs via SVC at the cluster level (“C”) or the peak level (“P”) (*p*_*FWE*_ < 0.05); marginally significant (“s”, “c”, or “p” in lower case) (0.05 <*p*_*FWE*_ < 0.10) or uncorrected (“U”) (*p* < 0.005) results are listed as well if the most stringent threshold for SVC was not attained. Left (“L”), right (“R”), and bilateral (“B”) refer to hemispheres for each ROI. The conventions for displaying this table also apply for Tables S21, S22, and S25. This table is related to Figures 8, 9, and S6 and Tables S16 and S17.
**Table S19**. Neural substrates of the GRL model: 3‐T Face/House version. This table is related to Figure 10 and Table S21.
**Figure S7**. Neural substrates of the GRL model: 7‐T Color/Motion version (Dopaminergic midbrain). (a) At 7 T, interaction effects between RPE signals and state generalization were localized to both the SN and the ventral tegmental area (VTA) (*p* < 0.005). (b) Interaction effects between RPE signals and action generalization were likewise observed in both the SN and the VTA (*p* < 0.005). This figure is related to Figure 10 and Tables S20 and S21.
**Table S20**. Neural substrates of the GRL model: 7‐T Color/Motion version. This table is related to Figures 10 and S7 and Table S21.
**Table S21**. Neural substrates of the GRL model: Summary (Basal ganglia). The second portion of the fMRI analyses are first summarized for the basal ganglia as further validation of the GRL model. As these effects lack precedent, the ROIs (as before) originated from a prior study that modeled the RPE without including any effects of generalization. This table is related to Figures 10 and S7 and Tables S19 and S20.
**Table S22**. Neural substrates of the GRL model: Summary (Hippocampus). This qualitative summary of the second portion of the fMRI analyses examines the hippocampus. This table is related to Figure 10 and Tables S19 and S20.
**Table S23**. Neural substrates of the learning rate: 3‐T Face/House version. This table is related to Table S25.
**Table S24**. Neural substrates of the learning rate: 7‐T Color/Motion version. This table is related to Table S25.
**Table S25**. Neural substrates of the learning rate: Summary. The absence of overlap between specific effects of generalization and effects of learning performance indicates that the former are not confounded with the latter. This table is related to Tables S23 and S24.

## Data Availability

Data are available at https://neurovault.org/collections/RLVWMYCQ/.
