# Supplementary material for: Reinforcement learning with associative or discriminative generalization across states and actions: fMRI at 3 T and 7 T
Source: Hum Brain Mapp. 2022 Jul 21;43(15):4750–90. doi: 10.1002/hbm.25988 (PMC9491297; doi:10.1002/hbm.25988)
Supplement: Supplementary file 1 — Table S1. Model comparison: 3‐T Face/House version (Good‐learner group). Listed first for 3 nonlearning models and 17 learning models fitted to empirical data are absolute scores for deviance and the corrected Akaike information criterion (AICc) (where a lower score is better). These absolute scores were translated to residual goodness of fit relative to the hysteresis model (where a higher score is better). Winning results determined by the AICc are highlighted with boldface and italics. “df” stands for degrees of freedom. This table is related to Figure 3. The conventions for displaying this table also apply for Tables S2–S15. Table S2. Model comparison: 3‐T Face/House version (Poor‐learner group). This table is related to Figure 3. Table S3. Model comparison: 3‐T Face/House version (Nonlearner group). Nonlearners were defined as such in cases where the hysteresis model provided the best fit post‐correction. Table S4. Model comparison: 7‐T Color/Motion version (Good‐learner group). This table is related to Figure 4. Table S5. Model comparison: 7‐T Color/Motion version (Poor‐learner group). This table is related to Figure 4. Figure S1. Discriminability of the GRL model: 3‐T Face/House version. Compare to Figure 3. Each fitted instantiation of the 7‐parameter “generalized reinforcement learning” (GRL) model (“AX|SY”) was used to simulate a data set yoked to that of the respective subject. Replications of the results from the original model comparison were achieved with these simulations as a demonstration of the discriminability of this preferred model with its additional degrees of freedom. This figure is related to Tables S6–S8. Table S6. Discriminability of the GRL model: 3‐T Face/House version (Good‐learner group). This table is related to Figure S1. Table S7. Discriminability of the GRL model: 3‐T Face/House version (Poor‐learner group). This table is related to Figure S1. Table S8. Discriminability of the GRL model: 3‐T Face/House version (Nonlearner group). C [file HBM-43-4750-s001.pdf]

Reinforcement learning with associative or discriminative generalization across states and actions: fMRI at 3 T and 7 T

Jaron T. Colas[1,2,3*], Neil M. Dundon[1,4], Raphael T. Gerraty[5,6,7], Natalie M. Saragosa-Harris[8,9], Karol P. Szymula[10], Koranis Tanwisuth[2,11], J. Michael Tyszka[2], Camilla van Geen[6,12], Harang Ju[13], Arthur W. Toga[14], Joshua I. Gold[15], Dani S. Bassett[10,16,17,18,19,20], Catherine A. Hartley[8,21], Daphna Shohamy[5,6,22], Scott T. Grafton[1**], & John P. O'Doherty[2,3**]

[1]Department of Psychological and Brain Sciences, University of California, Santa Barbara, CA
[2]Division of the Humanities and Social Sciences, California Institute of Technology, Pasadena, CA
[3]Computation and Neural Systems Program, California Institute of Technology, Pasadena, CA
[4]Department of Child and Adolescent Psychiatry, Psychotherapy, and Psychosomatics, University of Freiburg, Freiburg im Breisgau, Germany
[5]Department of Psychology, Columbia University, New York, NY
[6]Zuckerman Mind Brain Behavior Institute, Columbia University, New York, NY
[7]Center for Science and Society, Columbia University, New York, NY
[8]Department of Psychology, New York University, New York, NY
[9]Department of Psychology, University of California, Los Angeles, CA
[10]Department of Bioengineering, University of Pennsylvania, Philadelphia, PA
[11]Department of Psychology, University of California, Berkeley, CA
[12]Department of Psychology, University of Pennsylvania, Philadelphia, PA
[13]Neuroscience Graduate Group, University of Pennsylvania, Philadelphia, PA
[14]Laboratory of Neuro Imaging, USC Stevens Neuroimaging and Informatics Institute, Keck School of Medicine of USC, University of Southern California, Los Angeles, CA
[15]Department of Neuroscience, University of Pennsylvania, Philadelphia, PA
[16]Department of Electrical and Systems Engineering, University of Pennsylvania, Philadelphia, PA
[17]Department of Neurology, University of Pennsylvania, Philadelphia, PA
[18]Department of Psychiatry, University of Pennsylvania, Philadelphia, PA
[19]Department of Physics and Astronomy, University of Pennsylvania, Philadelphia, PA
[20]Santa Fe Institute, Santa Fe, NM
[21]Center for Neural Science, New York University, New York, NY
[22]Kavli Institute for Brain Science, Columbia University, New York, NY

*Correspondence: jcolas@ucsb.edu
**Co-senior authors

2022, *Human Brain Mapping*

https://doi.org/10.1002/hbm.25988

Supporting Information:
Supplementary Figures and Tables

3-T Face/House		Good learner (*n* = 31)			
Empirical data		Absolute		Residual	
Model	df	Dev.	AICc	Dev.	AICc
Chance	0	501.26	501.26	-11.46	-3.35
Intercept	1	497.80	499.81	-8.00	-1.90
Hysteresis	4	489.80	497.91	0	0
A0 I S0	5	427.99	438.16	61.81	59.76
A- I S0	5	434.12	444.30	55.67	53.62
AX I S0	6	422.57	434.81	67.23	63.10
A0 I S+	5	485.94	496.11	3.86	1.80
A0 I S-	5	432.66	442.83	57.14	55.08
A0 I SY	6	422.67	434.91	67.12	63.01
A- I S+	5	487.87	498.04	1.93	-0.13
A- I S-	5	444.43	454.60	45.37	43.31
AW I SW	6	421.49	433.73	68.31	64.18
AX I SY	7	***419.25***	***433.57***	***70.55***	***64.34***
AX I SY I Z	8	417.92	434.33	71.88	63.58
SPE	5	453.01	463.18	36.79	34.73
SPE+RL	7	421.59	435.91	68.21	62.00
HMM0	5	468.23	478.41	21.56	19.51
HMM	6	439.55	451.79	50.25	46.12
HMM0+RL	7	426.46	440.78	63.34	57.13
HMM+RL	8	420.13	436.54	69.67	61.37

Table S1. Model comparison: 3-T Face/House version (Good-learner group). Listed first for 3 nonlearning models and 17 learning models fitted to empirical data are absolute scores for deviance and the corrected Akaike information criterion (AICc) (where a lower score is better). These absolute scores were translated to residual goodness of fit relative to the hysteresis model (where a higher score is better). Winning results determined by the AICc are highlighted with boldface and italics. "df" stands for degrees of freedom. This table is related to Figure 3. The conventions for displaying this table also apply for Tables S2-S15.

3-T Face/House Empirical data		Poor learner (*n* = 9)			
		Absolute		Residual	
Model	df	Dev.	AICc	Dev.	AICc
Chance	0	489.05	489.05	-39.39	-31.27
Intercept	1	469.39	471.41	-19.73	-13.62
Hysteresis	4	449.67	457.78	0	0
A0 I S0	5	425.49	435.67	24.17	22.12
A- I S0	5	438.05	448.22	11.62	9.56
AX I S0	6	424.63	436.88	25.03	20.90
A0 I S+	5	446.84	457.01	2.83	0.77
A0 I S-	5	446.79	456.97	2.87	0.82
A0 I SY	6	423.03	435.28	26.64	22.51
A- I S+	5	446.13	456.30	3.54	1.48
A- I S-	5	448.97	459.15	0.69	-1.36
AW I SW	6	***422.23***	***434.48***	***27.44***	***23.31***
AX I SY	7	420.73	435.06	28.94	22.73
AX I SY I Z	8	420.12	436.54	29.55	21.24
SPE	5	449.56	459.74	0.10	-1.96
SPE+RL	7	425.46	439.79	24.20	17.99
HMM0	5	445.87	456.05	3.79	1.73
HMM	6	445.37	457.61	4.30	0.17
HMM0+RL	7	425.40	439.73	24.26	18.05
HMM+RL	8	424.46	440.88	25.21	16.90

Table S2. Model comparison: 3-T Face/House version (Poor-learner group). This table is related to Figure 3.

3-T Face/House		Nonlearner (n = 7)			
Empirical data		Absolute		Residual	
Model	df	Dev.	AICc	Dev.	AICc
Chance	0	481.64	481.64	-28.92	-20.80
Intercept	1	468.01	470.03	-15.29	-9.19
Hysteresis	4	***452.72***	***460.84***	***0***	***0***
A0 I S0	5	452.50	462.68	0.22	-1.84
A- I S0	5	452.51	462.69	0.21	-1.85
AX I S0	6	452.42	464.67	0.31	-3.83
A0 I S+	5	452.52	462.70	0.20	-1.86
A0 I S-	5	452.53	462.71	0.19	-1.87
A0 I SY	6	452.12	464.37	0.60	-3.53
A- I S+	5	452.43	462.61	0.29	-1.77
A- I S-	5	452.48	462.66	0.24	-1.82
AW I SW	6	452.06	464.31	0.66	-3.47
AX I SY	7	451.26	465.60	1.46	-4.76
AX I SY I Z	8	450.51	466.94	2.21	-6.10
SPE	5	452.51	462.69	0.21	-1.85
SPE+RL	7	452.37	466.71	0.35	-5.87
HMM0	5	452.44	462.62	0.28	-1.77
HMM	6	452.31	464.56	0.42	-3.72
HMM0+RL	7	452.50	466.84	0.22	-6.00
HMM+RL	8	452.21	468.64	0.51	-7.80

Table S3. Model comparison: 3-T Face/House version (Nonlearner group). Nonlearners were defined as such in cases where the hysteresis model provided the best fit post-correction.

7-T Color/Motion		Good learner (n = 16)			
Empirical data		Absolute		Residual	
Model	df	Dev.	AICc	Dev.	AICc
Chance	0	478.79	478.79	-17.13	-9.01
Intercept	1	470.80	472.82	-9.14	-3.04
Hysteresis	4	461.66	469.78	0	0
A0 I S0	5	423.83	434.01	37.83	35.77
A- I S0	5	404.11	414.29	57.55	55.49
AX I S0	6	399.51	411.76	62.15	58.02
A0 I S+	5	457.19	467.37	4.47	2.41
A0 I S-	5	419.36	429.54	42.30	40.24
A0 I SY	6	409.20	421.45	52.47	48.33
A- I S+	5	459.62	469.80	2.04	-0.02
A- I S-	5	421.81	431.99	39.85	37.79
AW I SW	6	404.13	416.39	57.53	53.40
AX I SY	7	***396.84***	***411.17***	***64.82***	***58.61***
AX I SY I Z	8	395.32	411.76	66.34	58.02
SPE	5	433.90	444.08	27.76	25.70
SPE+RL	7	407.90	422.23	53.76	47.55
HMM0	5	453.50	463.68	8.16	6.10
HMM	6	422.17	434.42	39.39	35.36
HMM0+RL	7	422.06	436.40	39.60	33.38
HMM+RL	8	407.47	423.91	54.19	45.87

Table S4. Model comparison: 7-T Color/Motion version (Good-learner group). This table is related to Figure 4.

7-T Color/Motion		Poor learner (*n* = 5)			
Empirical data		Absolute		Residual	
Model	df	Dev.	AICc	Dev.	AICc
Chance	0	447.50	447.50	-50.57	-42.43
Intercept	1	428.76	430.78	-31.83	-25.71
Hysteresis	4	396.93	405.06	0	0
A0 I S0	5	393.22	403.42	3.71	1.64
A- I S0	5	394.36	404.56	2.57	0.51
AX I S0	6	391.58	403.86	5.35	1.20
A0 I S+	5	391.06	401.26	5.87	3.80
A0 I S-	5	394.82	405.02	2.11	0.04
A0 I SY	6	387.78	400.06	9.15	5.00
A- I S+	5	392.46	402.66	4.47	2.41
A- I S-	5	394.99	405.19	1.94	-0.13
AW I SW	6	***387.72***	***400.00***	***9.21***	***5.06***
AX I SY	7	386.62	401.00	10.31	4.07
AX I SY I Z	8	384.06	400.55	12.87	4.51
SPE	5	394.81	405.01	2.12	0.05
SPE+RL	7	391.80	406.18	5.13	-1.12
HMM0	5	396.71	406.91	0.22	-1.84
HMM	6	394.83	407.11	2.10	-2.05
HMM0+RL	7	393.22	407.60	3.71	-2.54
HMM+RL	8	391.81	408.30	5.12	-3.23

Table S5. Model comparison: 7-T Color/Motion version (Poor-learner group). This table is related to Figure 4.

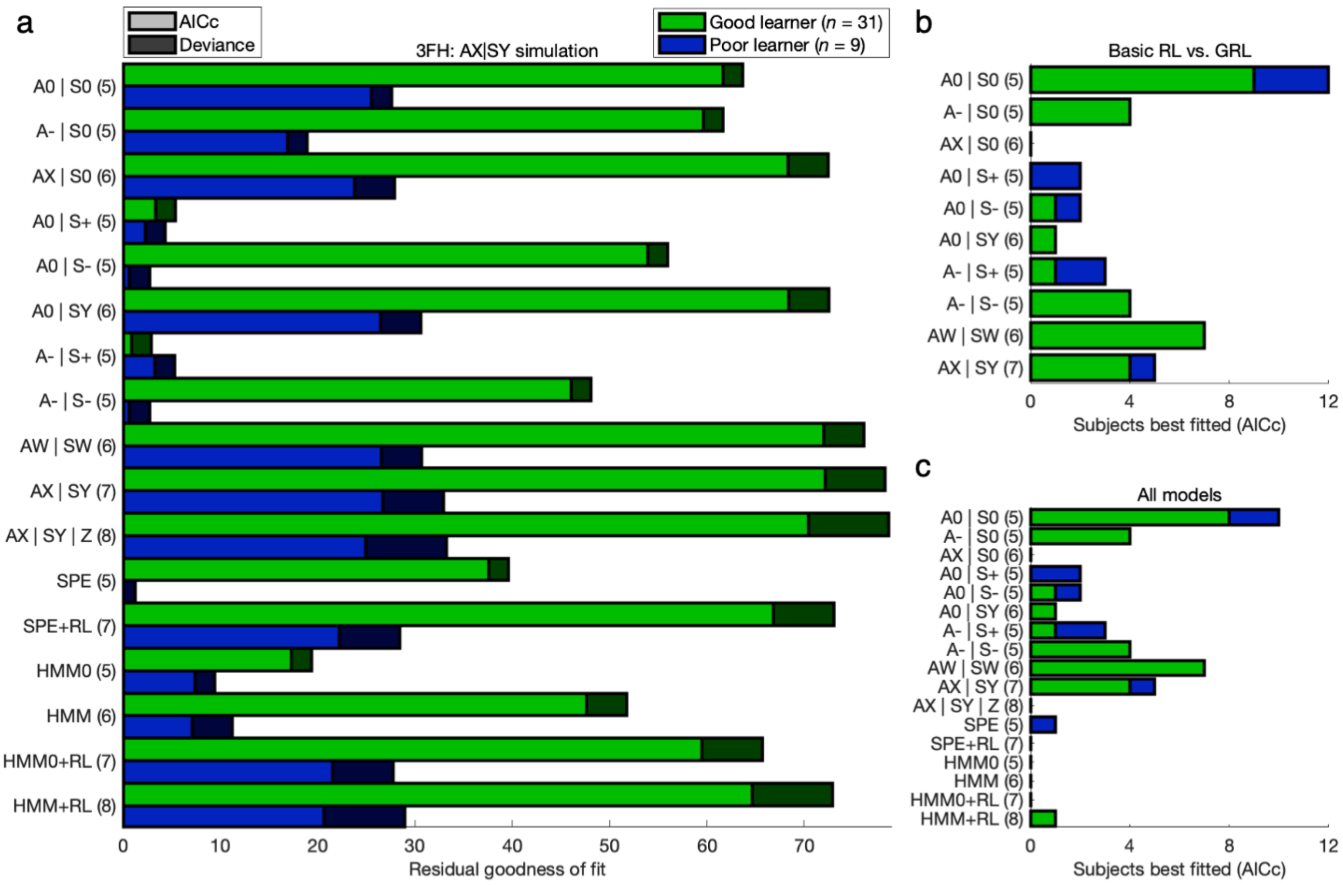
a
AICc
Deviance
3FH: AX|SY simulation
Good learner (n = 31)
Poor learner (n = 9)
A0 | S0 (5)
A- | S0 (5)
AX | S0 (6)
A0 | S+ (5)
A0 | S- (5)
A0 | SY (6)
A- | S+ (5)
A- | S- (5)
AW | SW (6)
AX | SY (7)
AX | SY | Z (8)
SPE (5)
SPE+RL (7)
HMM0 (5)
HMM (6)
HMM0+RL (7)
HMM+RL (8)
0
10
20
30
40
50
60
70
Residual goodness of fit
b
Basic RL vs. GRL
0
4
8
12
Subjects best fitted (AICc)
c
All models
Subjects best fitted (AICc)

Figure S1. Discriminability of the GRL model: 3-T Face/House version. Compare to Figure 3. Each fitted instantiation of the 7-parameter "generalized reinforcement learning" (GRL) model ("AX I SY") was used to simulate a data set yoked to that of the respective subject. Replications of the results from the original model comparison were achieved with these simulations as a demonstration of the discriminability of this preferred model with its additional degrees of freedom. This figure is related to Tables S6-S8.

3-T Face/House<br>AX I SY sim.<br>Model	df	Good learner (n = 31)<br>Absolute<br>Dev.	 <br>AICc	 Residual<br>Dev.	 <br>AICc
Chance	0	501.26	501.26	-14.25	-6.14
Intercept	1	496.29	498.30	-9.29	-3.18
Hysteresis	4	487.01	495.12	0	0
A0 I S0	5	423.30	433.47	63.71	61.65
A- I S0	5	425.33	435.51	61.67	59.61
AX I S0	6	414.55	426.79	72.46	68.33
A0 I S+	5	481.62	491.80	5.38	3.32
A0 I S-	5	431.04	441.21	55.96	53.90
A0 I SY	6	414.51	426.75	72.49	68.36
A- I S+	5	484.09	494.26	2.92	0.86
A- I S-	5	438.90	449.07	48.11	46.05
AW I SW	6	410.90	423.14	76.10	71.98
AX I SY	7	***408.68***	***423.01***	***78.32***	***72.11***
AX I SY I Z	8	408.32	424.73	78.69	70.38
SPE	5	447.36	457.53	39.64	37.58
SPE+RL	7	414.00	428.32	73.01	66.80
HMM0	5	467.64	477.81	19.37	17.31
HMM	6	435.28	447.52	51.73	47.60
HMM0+RL	7	421.33	435.65	65.67	59.46
HMM+RL	8	414.09	430.51	72.91	64.61

Table S6. Discriminability of the GRL model: 3-T Face/House version (Good-learner group). This table is related to Figure S1.

3-T Face/House AX I SY sim. Model	df	Poor learner (n = 9) Absolute Dev.	AICc	Residual Dev.	AICc
Chance	0	489.05	489.05	-42.53	-34.41
Intercept	1	461.11	463.12	-14.58	-8.48
Hysteresis	4	446.53	454.64	0	0
A0 I S0	5	418.93	429.11	27.60	25.54
A- I S0	5	427.58	437.76	18.94	16.88
AX I S0	6	418.58	430.83	27.95	23.82
A0 I S+	5	442.22	452.39	4.31	2.25
A0 I S-	5	443.81	453.99	2.72	0.66
A0 I SY	6	415.89	428.13	30.64	26.51
A- I S+	5	441.22	451.39	5.31	3.25
A- I S-	5	443.81	453.99	2.72	0.66
AW I SW	6	415.83	428.07	30.70	26.57
AX I SY	7	***413.59***	***427.92***	***32.94***	***26.73***
AX I SY I Z	8	413.31	429.73	33.22	24.91
SPE	5	445.26	455.44	1.27	-0.79
SPE+RL	7	418.09	432.42	28.44	22.22
HMM0	5	437.09	447.26	9.44	7.38
HMM	6	435.31	446.56	11.21	7.08
HMM0+RL	7	418.76	433.09	27.77	21.55
HMM+RL	8	417.53	433.96	28.99	20.69

Table S7. Discriminability of the GRL model: 3-T Face/House version (Poor-learner group). This table is related to Figure 1.

3-T Face/House AX I SY sim. Model	df	Nonlearner (n = 7) Absolute Dev.	Absolute AICc	Residual Dev.	Residual AICc
Chance	0	481.64	481.64	-33.47	-25.36
Intercept	1	466.24	468.25	-18.07	-11.97
Hysteresis	4	***448.16***	***456.28***	***0***	***0***
A0 I S0	5	447.48	457.66	0.68	-1.38
A- I S0	5	446.77	456.94	1.40	-0.66
AX I S0	6	446.46	458.71	1.70	-2.43
A0 I S+	5	447.20	457.38	0.96	-1.10
A0 I S-	5	447.24	457.42	0.92	-1.14
A0 I SY	6	445.30	457.55	2.86	-1.27
A- I S+	5	446.73	456.91	1.43	-0.63
A- I S-	5	447.32	457.50	0.84	-1.22
AW I SW	6	445.04	457.29	3.13	-1.01
AX I SY	7	443.28	457.61	4.89	-1.33
AX I SY I Z	8	442.44	458.87	5.72	-2.59
SPE	5	447.45	457.63	0.71	-1.34
SPE+RL	7	446.82	461.15	1.35	-4.87
HMM0	5	447.99	458.17	0.17	-1.89
HMM	6	447.43	459.68	0.73	-3.40
HMM0+RL	7	447.48	461.81	0.68	-5.53
HMM+RL	8	447.04	463.47	1.12	-7.19

Table S8. Discriminability of the GRL model: 3-T Face/House version (Nonlearner group). Compare to Table S3. The hysteresis model also provided the best fit for Nonlearners in silico.

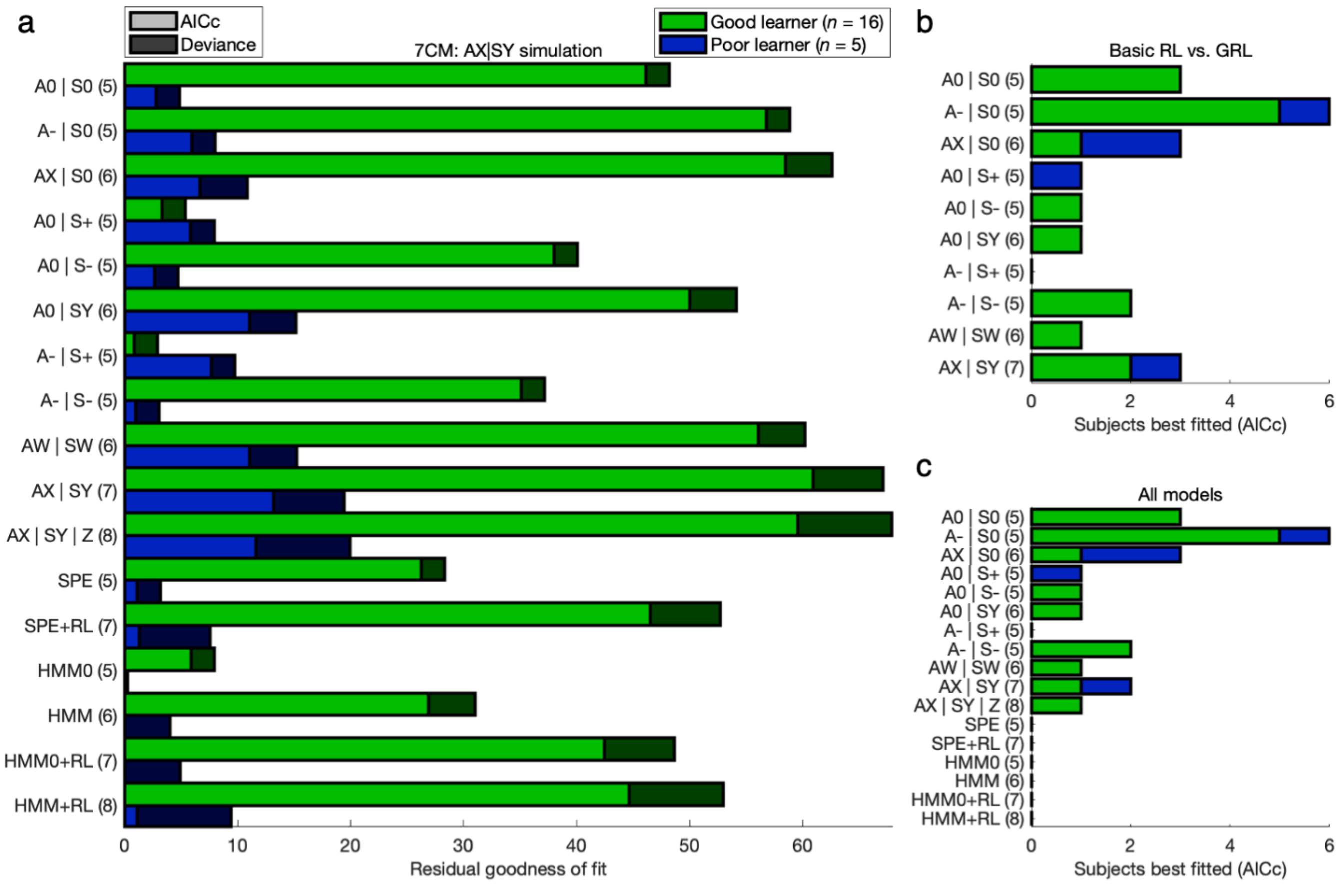

a
AICc
Deviance
Good learner (n = 16)
Poor learner (n = 5)
7CM: AX|SY simulation
A0 | S0 (5)
A- | S0 (5)
AX | S0 (6)
A0 | S+ (5)
A0 | S- (5)
A0 | SY (6)
A- | S+ (5)
A- | S- (5)
AW | SW (6)
AX | SY (7)
AX | SY | Z (8)
SPE (5)
SPE+RL (7)
HMM0 (5)
HMM (6)
HMM0+RL (7)
HMM+RL (8)
0 10 20 30 40 50 60
Residual goodness of fit
b
Basic RL vs. GRL
Subjects best fitted (AICc)
c
All models
Subjects best fitted (AICc)

Figure S2. Discriminability of the GRL model: 7-T Color/Motion version. Compare to Figures 4 and S1. This figure is related to Tables S9 and S10.

7-T Color/Motion AX I SY sim. Model	df	Good learner (n = 16) Absolute Dev.	AICc	Residual Dev.	AICc
Chance	0	478.79	478.79	-19.00	-10.88
Intercept	1	472.41	474.42	-12.62	-6.51
Hysteresis	4	459.79	467.81	0	0
A0 I S0	5	411.55	421.72	48.24	46.18
A- I S0	5	400.91	411.09	58.88	56.82
AX I S0	6	397.20	409.45	62.59	58.46
A0 I S+	5	454.41	464.59	5.38	3.32
A0 I S-	5	419.71	429.89	40.07	38.01
A0 I SY	6	405.64	417.89	54.15	50.01
A- I S+	5	456.83	467.01	2.96	0.90
A- I S-	5	422.61	432.79	37.17	35.11
AW I SW	6	399.57	411.82	60.22	56.08
AX I SY	7	***392.63***	***406.96***	***67.16***	***60.94***
AX I SY I Z	8	391.86	408.30	57.92	59.61
SPE	5	431.43	441.60	28.36	26.30
SPE+RL	7	407.04	421.38	52.75	46.53
HMM0	5	451.83	462.01	7.96	5.90
HMM	6	428.73	440.98	31.06	26.93
HMM0+RL	7	411.10	425.44	48.68	42.47
HMM+RL	8	406.78	423.22	53.00	44.69

Table S9. Discriminability of the GRL model: 7-T Color/Motion version (Good-learner group). This table is related to Figure S2.

7-T Color/Motion		Poor learner (n = 5)			
AX I SY sim.		Absolute		Residual	
Model	df	Dev.	AICc	Dev.	AICc
Chance	0	447.50	447.50	-56.38	-48.25
Intercept	1	420.10	422.11	-28.99	-22.87
Hysteresis	4	391.11	399.25	0	0
A0 I S0	5	386.21	396.41	4.90	2.84
A- I S0	5	383.08	393.28	8.03	5.97
AX I S0	6	380.26	392.54	10.86	6.71
A0 I S+	5	383.18	393.38	7.94	5.87
A0 I S-	5	386.37	396.57	4.74	2.68
A0 I SY	6	375.92	388.20	15.19	11.04
A- I S+	5	381.32	391.52	9.80	7.73
A- I S-	5	388.05	398.25	3.06	0.99
AW I SW	6	375.88	388.17	15.23	11.08
AX I SY	7	***371.68***	***386.06***	***19.43***	***13.19***
AX I SY I Z	8	371.12	387.60	20.00	11.64
SPE	5	387.89	398.09	3.22	1.15
SPE+RL	7	383.55	397.93	7.56	1.32
HMM0	5	390.85	401.05	0.27	-1.80
HMM	6	387.05	399.33	4.06	-0.09
HMM0+RL	7	386.21	400.59	4.91	-1.34
HMM+RL	8	381.66	398.15	9.45	1.10

Table S10. Discriminability of the GRL model: 7-T Color/Motion version (Poor-learner group). This table is related to Figure S2.

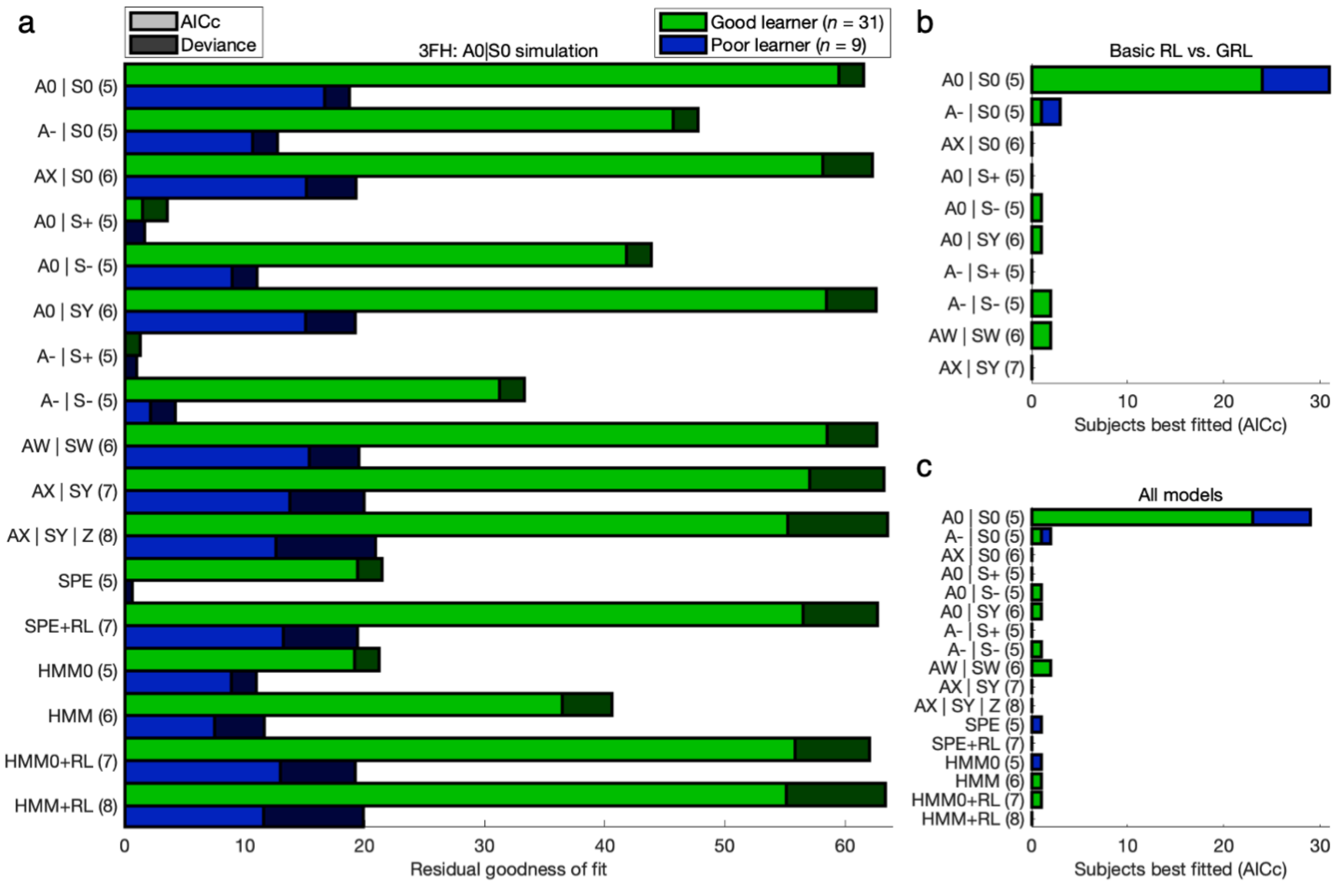
a
AICc
Deviance
Good learner (n = 31)
Poor learner (n = 9)
3FH: A0|S0 simulation
A0 | S0 (5)
A- | S0 (5)
AX | S0 (6)
A0 | S+ (5)
A0 | S- (5)
A0 | SY (6)
A- | S+ (5)
A- | S- (5)
AW | SW (6)
AX | SY (7)
AX | SY | Z (8)
SPE (5)
SPE+RL (7)
HMM0 (5)
HMM (6)
HMM0+RL (7)
HMM+RL (8)
0
10
20
30
40
50
60
Residual goodness of fit
b
Basic RL vs. GRL
Subjects best fitted (AICc)
c
All models
Subjects best fitted (AICc)

Figure S3. Discriminability of the basic RL model: 3-T Face/House version. Compare to Figure S1. The basic RL model was recovered in lieu of the GRL model when substituting data simulated with basic RL. This converse model recovery again demonstrates an absence of overfitting. This figure is related to Tables S11-S13.

3-T Face/House A0 I S0 sim.		Good learner (n = 31)			
		Absolute		Residual	
Model	df	Dev.	AICc	Dev.	AICc
Chance	0	501.26	501.26	-14.78	-6.67
Intercept	1	496.62	498.63	-10.15	-4.04
Hysteresis	4	486.47	494.59	0	0
A0 I S0	5	***424.94***	***435.11***	***61.53***	***59.47***
A- I S0	5	438.74	448.91	47.73	45.67
AX I S0	6	424.23	436.47	62.24	58.12
A0 I S+	5	482.94	493.11	3.54	1.48
A0 I S-	5	442.59	452.76	43.88	41.83
A0 I SY	6	423.93	436.17	62.55	58.42
A- I S+	5	485.14	495.31	1.33	-0.73
A- I S-	5	453.18	463.35	33.3	31.24
AW I SW	6	423.83	436.07	62.64	58.52
AX I SY	7	423.20	437.53	63.27	57.06
AX I SY I Z	8	422.92	439.34	63.55	55.25
SPE	5	465.01	475.18	21.47	19.41
SPE+RL	7	423.78	438.10	62.70	56.49
HMM0	5	465.23	475.41	21.24	19.18
HMM	6	445.88	458.12	40.59	36.47
HMM0+RL	7	424.45	438.77	62.02	55.81
HMM+RL	8	423.09	439.50	63.39	55.09

Table S11. Discriminability of the basic RL model: 3-T Face/House version (Good-learner group). This table is related to Figure S3.

3-T Face/House<br>A0 l S0 sim.<br>Model	df	Poor learner (*n* = 9)<br>Absolute<br>Dev.	 <br>AICc	 Residual<br>Dev.	 <br>AICc
Chance	0	489.05	489.05	-40.78	-32.66
Intercept	1	465.73	467.74	-17.45	-11.34
Hysteresis	4	448.28	456.39	0	0
A0 l S0	5	***429.53***	***439.71***	***18.74***	***16.69***
A- l S0	5	435.55	445.73	12.73	10.67
AX l S0	6	429.00	441.25	19.27	15.15
A0 l S+	5	446.60	456.77	1.68	-0.38
A0 l S-	5	437.27	447.45	11.00	8.95
A0 l SY	6	429.06	441.30	19.22	15.09
A- l S+	5	447.28	457.46	0.99	-1.06
A- l S-	5	444.05	454.23	4.22	2.17
AW l SW	6	428.74	440.99	19.53	15.40
AX l SY	7	428.31	442.64	19.97	13.76
AX l SY l Z	8	427.37	443.80	20.91	12.60
SPE	5	447.64	457.81	0.64	-1.42
SPE+RL	7	428.88	443.21	19.40	13.19
HMM0	5	437.34	447.51	10.94	8.88
HMM	6	436.64	448.89	11.63	7.50
HMM0+RL	7	429.09	443.42	19.19	12.98
HMM+RL	8	428.40	444.83	19.87	11.57

Table S12. Discriminability of the basic RL model: 3-T Face/House version (Poor-learner group). This table is related to Figure S3.

3-T Face/House<br>A0 I S0 sim.<br>Model	df	Nonlearner (n = 7)<br>Absolute<br>Dev.	 <br>AICc	 Residual<br>Dev.	 <br>AICc
Chance	0	481.64	481.64	-30.70	-22.58
Intercept	1	466.57	468.59	-15.63	-9.53
Hysteresis	4	***450.94***	***459.06***	***0***	***0***
A0 I S0	5	450.33	460.51	0.61	-1.45
A- I S0	5	450.52	460.70	0.42	-1.64
AX I S0	6	450.20	462.45	0.74	-3.39
A0 I S+	5	449.07	459.24	1.87	-0.18
A0 I S-	5	450.33	460.51	0.61	-1.45
A0 I SY	6	448.29	460.53	2.66	-1.48
A- I S+	5	450.91	461.09	0.03	-2.03
A- I S-	5	450.81	460.99	0.13	-1.93
AW I SW	6	448.35	460.60	2.59	-1.54
AX I SY	7	447.33	461.67	3.61	-2.61
AX I SY I Z	8	444.90	461.42	5.95	-2.37
SPE	5	450.59	460.76	0.36	-1.70
SPE+RL	7	450.14	464.47	0.80	-5.42
HMM0	5	450.88	461.06	0.06	-2.00
HMM	6	450.04	462.29	0.90	-3.23
HMM0+RL	7	450.33	464.66	0.61	-5.60
HMM+RL	8	449.42	465.85	1.52	-6.79

Table S13. Discriminability of the basic RL model: 3-T Face/House version (Nonlearner group). Compare to Tables S3 and S8. The hysteresis model also provided the best fit for Nonlearners in silico.

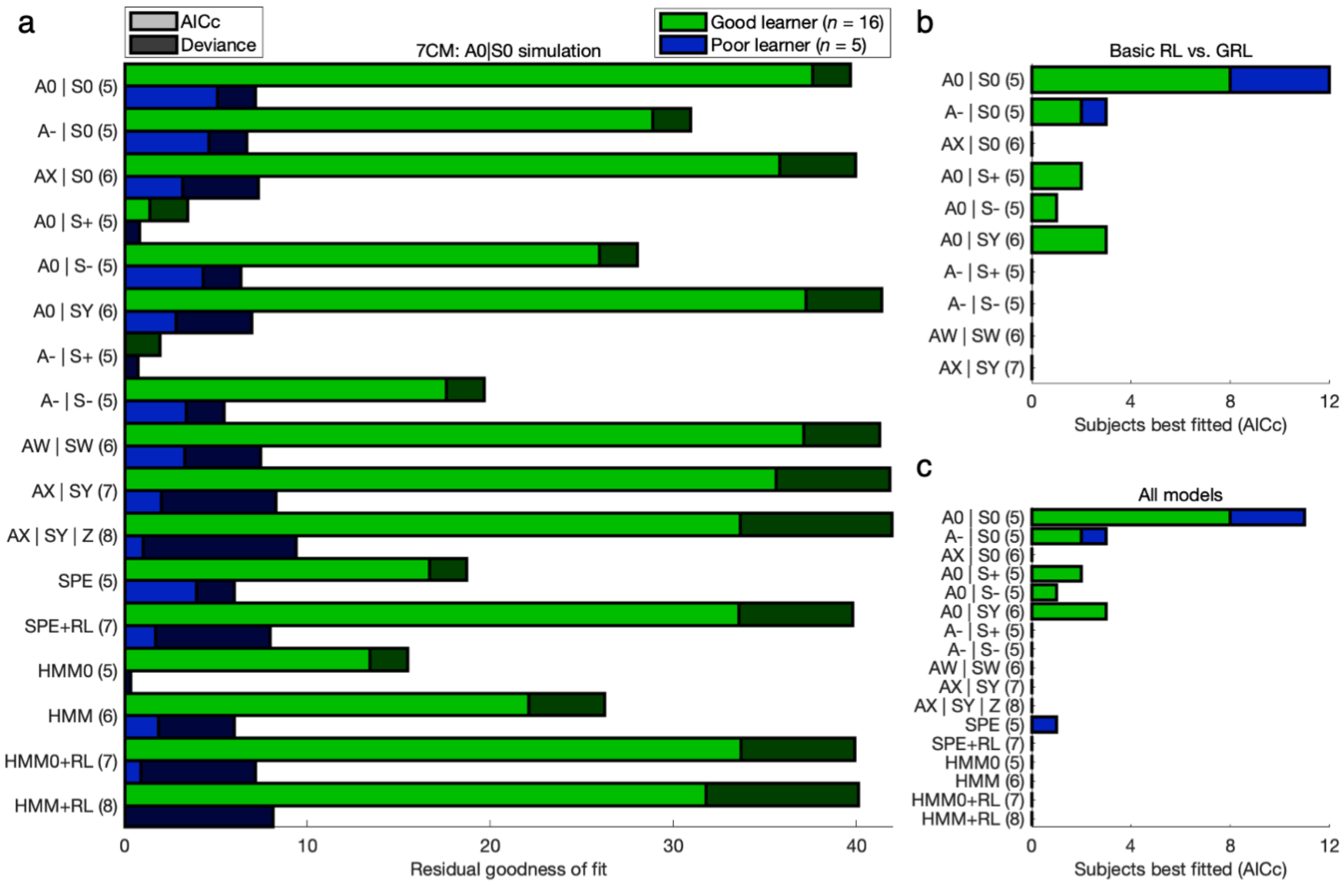
a
AICc
Deviance
Good learner (n = 16)
Poor learner (n = 5)
7CM: A0|S0 simulation
A0 | S0 (5)
A- | S0 (5)
AX | S0 (6)
A0 | S+ (5)
A0 | S- (5)
A0 | SY (6)
A- | S+ (5)
A- | S- (5)
AW | SW (6)
AX | SY (7)
AX | SY | Z (8)
SPE (5)
SPE+RL (7)
HMM0 (5)
HMM (6)
HMM0+RL (7)
HMM+RL (8)
0
10
20
30
40
Residual goodness of fit
b
Basic RL vs. GRL
A0 | S0 (5)
A- | S0 (5)
AX | S0 (6)
A0 | S+ (5)
A0 | S- (5)
A0 | SY (6)
A- | S+ (5)
A- | S- (5)
AW | SW (6)
AX | SY (7)
0
4
8
12
Subjects best fitted (AICc)
c
All models
A0 | S0 (5)
A- | S0 (5)
AX | S0 (6)
A0 | S+ (5)
A0 | S- (5)
A0 | SY (6)
A- | S+ (5)
A- | S- (5)
AW | SW (6)
AX | SY (7)
AX | SY | Z (8)
SPE (5)
SPE+RL (7)
HMM0 (5)
HMM (6)
HMM0+RL (7)
HMM+RL (8)
0
4
8
12
Subjects best fitted (AICc)

Figure S4. Discriminability of the basic RL model: 7-T Color/Motion version. Compare to Figures S2 and S3. This figure is related to Tables S14 and S15.

7-T Color/Motion<br>A0 I S0 sim.<br>Model	df	Good learner (n = 16)<br>Absolute<br>Dev.	 <br>AICc	 Residual<br>Dev.	 <br>AICc
Chance	0	478.79	478.79	-18.76	-10.64
Intercept	1	472.35	474.36	-12.31	-6.20
Hysteresis	4	460.03	468.15	0	0
A0 I S0	5	***420.37***	***430.55***	***39.66***	***37.60***
A- I S0	5	429.11	439.29	30.93	28.87
AX I S0	6	420.09	432.34	39.94	35.81
A0 I S+	5	456.58	466.76	3.45	1.39
A0 I S-	5	432.00	442.18	28.04	25.98
A0 I SY	6	418.66	430.91	41.38	37.24
A- I S+	5	458.12	468.30	1.92	-0.14
A- I S-	5	440.39	450.57	19.65	17.59
AW I SW	6	418.79	431.04	41.24	37.11
AX I SY	7	418.23	432.56	41.81	35.59
AX I SY I Z	8	418.07	434.51	41.96	33.64
SPE	5	441.31	451.49	18.72	16.66
SPE+RL	7	420.24	434.57	39.80	33.58
HMM0	5	444.57	454.75	15.46	13.40
HMM	6	433.79	446.04	26.24	22.11
HMM0+RL	7	420.14	434.48	39.89	33.67
HMM+RL	8	419.95	436.38	40.09	31.77

Table S14. Discriminability of the basic RL model: 7-T Color/Motion version (Good-learner group). This table is related to Figure S4.

7-T Color/Motion<br>A0 I S0 sim.<br>Model	df	Poor learner (n = 5)<br>Absolute<br>Dev.	 <br>AICc	 Residual<br>Dev.	 <br>AICc
Chance	0	447.50	447.50	-61.44	-53.31
Intercept	1	424.85	426.86	-38.79	-32.67
Hysteresis	4	386.05	394.19	0	0
A0 I S0	5	***378.90***	***389.10***	***7.16***	***5.09***
A- I S0	5	379.38	389.58	6.67	4.61
AX I S0	6	378.75	391.04	7.30	3.15
A0 I S+	5	385.23	395.43	0.83	-1.24
A0 I S-	5	379.69	389.89	6.37	4.30
A0 I SY	6	379.09	391.37	6.96	2.81
A- I S+	5	385.33	395.53	0.73	-1.34
A- I S-	5	380.61	390.82	5.44	3.37
AW I SW	6	378.63	390.91	7.42	3.27
AX I SY	7	377.78	392.16	8.27	2.03
AX I SY I Z	8	376.69	393.17	9.37	1.01
SPE	5	380.05	390.25	6.00	3.93
SPE+RL	7	378.11	392.49	7.94	1.70
HMM0	5	385.72	395.92	0.33	-1.74
HMM	6	380.04	392.32	6.01	1.87
HMM0+RL	7	378.89	393.27	7.16	0.91
HMM+RL	8	377.94	394.43	8.11	-0.24

Table S15. Discriminability of the basic RL model: 7-T Color/Motion version (Poor-learner group). This table is related to Figure S4.

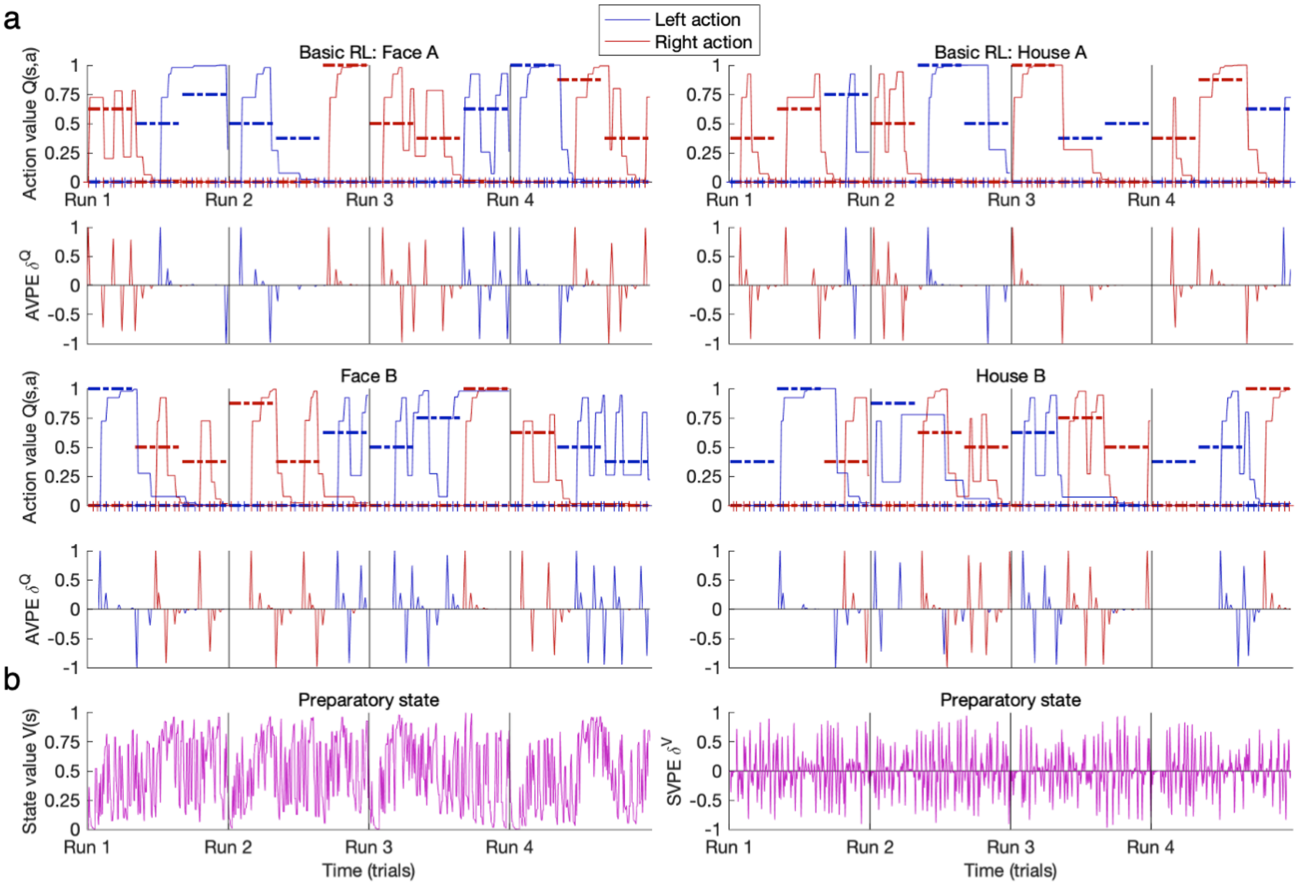
a
Left action
Right action
Basic RL: Face A
Basic RL: House A
Action value Q(s,a)
AVPE δ^Q
Face B
House B
b
Preparatory state
State value V(s)
SVPE δ^V
Run 1
Run 2
Run 3
Run 4
Time (trials)

Figure S5. Predictions of the basic RL model. Compare to Figure 7. Representative dynamics generated by the basic RL model ($g_A = g_S = 0$) are shown for the same participant. Parameters were assigned as follows for this participant: $\alpha = 0.724$, $\lambda = 0.500$, $\tau = 0.567$, $\beta_0 = -0.084$, $\lambda_\beta = 0.715$, and $\beta_R = 0.252$. Unlike GRL, basic RL updates the value of only the state-action pair experienced on a given trial.

Group (3-T Face/House)<br>Variable<br>Region	H	*x, y, z*	t_{df}	*p*	*k*	SVC
All learners (*n* = 40)			t_{39}			
Reward-prediction error						
Nucleus accumbens	L	-12, 14, -8	4.88	$< 10^{-5}$	128	CP
Nucleus accumbens	R	16, 12, -12	3.60	$< 10^{-3}$	15	CP
Dorsal putamen &<br>Dorsal caudate nucleus	R	26, 6, 4	3.98	10^{-4}	103	CP
Posterior putamen	R	26, -6, 4	3.08	0.002	15	-
Posterior putamen	L	-28, -13, 4	4.22	$< 10^{-4}$	28	-
Posterior putamen	R	30, -16, -4	3.46	$< 10^{-3}$	44	-
Value						
Frontopolar cortex	R	12, 66, 4	4.09	10^{-4}	106	-
Frontopolar cortex	L	-4, 64, 2	4.96	$< 10^{-5}$	108	-
Ventromedial prefrontal cortex	B	6, 52, -14	4.00	10^{-4}	118	CP
Anterior cingulate cortex	R	10, 46, 2	3.63	$< 10^{-3}$	10	-
Anterior caudate nucleus	L	-12, 24, 2	3.93	$< 10^{-3}$	18	-
Nucleus accumbens &<br>Ventral putamen	R	16, 16, -10	3.15	0.002	12	-
Nucleus accumbens	R	8, 14, -4	3.95	$< 10^{-3}$	30	CP
Posterior cingulate cortex	L	-2, -26, 38	3.46	$< 10^{-3}$	13	p
Posterior cingulate cortex	B	0, -38, 36	3.24	10^{-3}	21	Cp
Reaction time						
Medial frontal cortex &<br>Other regions	B	-6, 10, 54	9.13	$< 10^{-10}$	4575	CP
Good learners (*n* = 31)			t_{30}			
Reward-prediction error						
Nucleus accumbens	L	-12, 14, -8	3.70	$< 10^{-3}$	23	CP

Dorsal caudate nucleus & Dorsal putamen	L	-18, 6, 12	4.37	$< 10^{-4}$	34	CP
Dorsal putamen & Dorsal caudate nucleus	R	24, 6, 2	4.52	$< 10^{-4}$	138	CP
Posterior putamen	L	-28, -6, 6	4.78	$< 10^{-4}$	120	-
Substantia nigra	R	12, -4, -16	3.34	10^{-3}	17	-
Value						
Ventromedial prefrontal cortex	B	-6, 62, -2	6.48	$< 10^{-6}$	886	CP
Anterior cingulate cortex	L	-4, 40, 0	3.88	$< 10^{-3}$	21	-
Anterior caudate nucleus	L	-12, 24, 2	3.59	$< 10^{-3}$	12	-
Anterior caudate nucleus	R	14, 24, -6	4.25	$< 10^{-4}$	41	cp
Nucleus accumbens	R	10, 2, -14	5.21	$< 10^{-5}$	80	CP
Posterior cingulate cortex	B	2, -24, 34	3.94	$< 10^{-3}$	49	cP
Reaction time						
Medial frontal cortex & Other regions	B	-6, 10, 54	8.84	$< 10^{-9}$	8854	CP
Poor learners ($n = 9$)			t_8			
Reward-prediction error						
Nucleus accumbens	L	-8, 10, -6	4.64	$< 10^{-3}$	20	Cp
Value						
Frontopolar cortex	R	14, 66, -10	5.10	$< 10^{-3}$	10	-
Posterior cingulate cortex	L	-6, -40, 52	4.55	$< 10^{-3}$	11	-
Reaction time						
Medial frontal cortex	R	2, 28, 38	8.93	$< 10^{-5}$	70	-
Dorsal anterior cingulate cortex	L	-10, 18, 34	9.43	$< 10^{-5}$	35	-
Medial frontal cortex	R	6, 14, 46	5.60	$< 10^{-3}$	13	U
Medial frontal cortex	L	-6, 6, 52	7.72	$< 10^{-4}$	101	CP
Nonlearners ($n = 7$)			t_6			

Reaction time						
Medial frontal cortex	L	-12, 8, 52	10.86	$< 10^{-4}$	13	-
Medial frontal cortex	B	0, 2, 56	7.41	$< 10^{-3}$	54	-
Medial frontal cortex	L	-8, -6, 62	6.15	$< 10^{-3}$	23	-

Table S16. Neural substrates of the RL framework: 3-T Face/House version. Listed for every significant cluster ($p < 0.005$, $k \geq 10$) are anatomical regions; hemispheres ("H") as left ("L"), right ("R"), or bilateral ("B"); stereotactic coordinates in MNI space in mm (*x*, *y*, *z*); test statistics (t_{df}); probability values (*p*); cluster extents in voxels (*k*); and results of small-volume correction (SVC) at the cluster level ("C") or the peak level ("P") ($p_{FWE} < 0.05$), where marginally significant ("c" or "p" in lower case) ($0.05 < p_{FWE} < 0.10$) or uncorrected ("U") ($p < 0.005$) results are also listed if the most stringent threshold for SVC was not attained within the region of interest. All relevant groupings of participants are included. The conventions for displaying this table also apply for Tables S17, S19, S20, S23, and S24. This table is related to Figure 8 and Table S18.

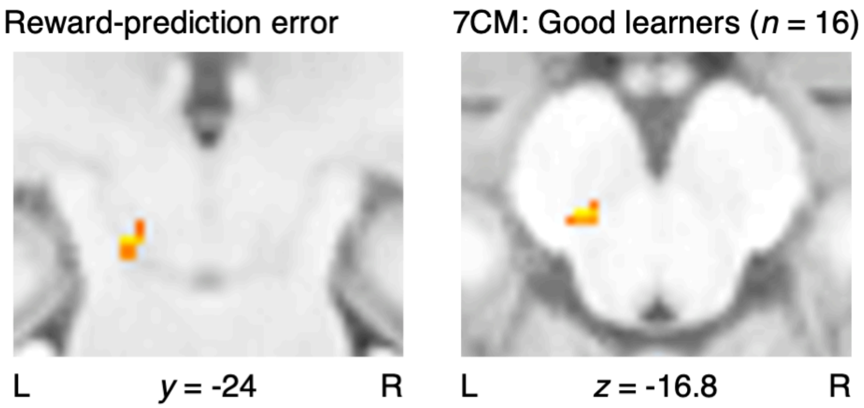
Reward-prediction error
7CM: Good learners (n = 16)
L
y = -24
R
L
z = -16.8
R

Figure S6. Neural substrates of the RL framework: 7-T Color/Motion version (Dopaminergic midbrain). At 7 T, reward-prediction error (RPE) signals from the GRL model were further localized to the substantia nigra (SN) ($p < 0.005$). This figure is related to Figure 9 and Tables S17 and S18.

Group (7-T Color/Motion)<br>Variable<br>Region	H	x, y, z	t_{df}	p	k	SVC
All learners (n = 21)			t_{20}			
Reward-prediction error						
Nucleus accumbens	R	8.4, 10.8, -6	4.91	$< 10^{-4}$	44	CP
Dorsal putamen	L	-25, 4.8, 7.2	6.20	$< 10^{-5}$	11	-
Posterior putamen	L	-30, -1.2, 6	4.97	$< 10^{-4}$	19	-
Posterior putamen	L	-27.6, -9.6, 7.2	5.86	$< 10^{-5}$	10	-
Posterior putamen	R	27.6, -9.6, -8.4	5.01	$< 10^{-4}$	20	-
Value						
Ventromedial prefrontal cortex	L	-4.8, 49.2, -12	3.71	$< 10^{-3}$	17	U
Ventromedial prefrontal cortex	R	6, 46.8, -12	6.22	$< 10^{-5}$	12	U
Anterior cingulate cortex	L	-6, 38.4, -10.8	4.37	10^{-4}	13	U
Nucleus accumbens	R	8.4, 15.6, -4.8	4.09	$< 10^{-3}$	19	C
Nucleus accumbens	R	10.8, 8.4, -9.6	4.33	$< 10^{-3}$	14	U
Dorsal putamen	R	22.8, 8.4, -4.8	4.47	10^{-4}	19	-
Ventral putamen	R	15.6, 6, -9.6	4.19	$< 10^{-3}$	50	-
Nucleus accumbens	L	-10.8, 4.8, -10.8	4.52	10^{-4}	59	C
Ventral putamen	L	-18, 4.8, -10.8	4.36	10^{-4}	25	-
Posterior cingulate cortex	B	0, -45.6, 30	3.94	$< 10^{-3}$	23	-
Reaction time						
Medial frontal cortex	R	8.4, 31.2, 43.2	4.97	$< 10^{-4}$	19	-
Medial frontal cortex	B	-3.6, 26.4, 49.2	4.29	$< 10^{-3}$	12	-
Medial frontal cortex	L	-4.8, 25.2, 40.8	4.61	$< 10^{-4}$	27	-
Medial frontal cortex	R	7.2, 24, 38.4	7.98	$< 10^{-7}$	1885	CP
Dorsal anterior cingulate cortex	L	-12, 21.6, 31.2	3.35	0.002	18	-
Medial frontal cortex	L	-6, 18, 33.6	4.43	10^{-4}	12	-

Medial frontal cortex	L	-9.6, 14.4, 57.6	4.07	$< 10^{-3}$	24	-
Dorsal anterior cingulate cortex	R	3.6, 13.2, 24	4.56	$< 10^{-4}$	12	-
Medial frontal cortex	L	-9.6, 10.8, 48	8.09	$< 10^{-7}$	1620	CP
Medial frontal cortex	R	18, 3.6, 70.8	6.47	10^{-6}	181	-
Medial frontal cortex	L	-2.4, 2.4, 37.2	3.77	$< 10^{-3}$	11	-
Medial frontal cortex	R	14.4, -14.4, 48	3.79	$< 10^{-3}$	11	-
Good learners (*n* = 16)			t_{15}			
Reward-prediction error						
Nucleus accumbens & Anterior caudate nucleus	R	7.2, 13.2, -4.8	5.70	$< 10^{-4}$	63	CP
Dorsal caudate nucleus	L	12, 10.8, 0	4.64	$< 10^{-3}$	16	p
Nucleus accumbens	L	-8.4, 3.6, -3.6	4.67	$< 10^{-3}$	18	p
Ventral putamen	R	24, 3.6, -9.6	5.11	$< 10^{-4}$	17	-
Posterior putamen	L	-30, -1.2, 6	4.41	$< 10^{-3}$	10	-
Posterior putamen	R	26.4, -2.4, -6	3.66	10^{-3}	11	-
Posterior putamen	R	28.8, -7.2, -7.2	4.38	$< 10^{-3}$	18	-
Posterior putamen	L	-28.8, -9.6, 13.2	4.62	$< 10^{-3}$	11	-
Substantia nigra	L	-9.6, -24, -16.8	4.80	10^{-4}	11	-
Value						
Ventromedial prefrontal cortex	L	-2.4, 50.4, -1.2	4.76	10^{-4}	16	-
Ventromedial prefrontal cortex	R	6, 46.8, -12	5.85	$< 10^{-4}$	10	U
Ventromedial prefrontal cortex	L	-2.4, 44.4, -10.8	4.99	$< 10^{-4}$	46	CP
Anterior cingulate cortex	B	0, 38.4, 7.2	3.70	10^{-3}	12	-
Ventromedial prefrontal cortex	L	-4.8, 38.4, -9.6	5.11	$< 10^{-4}$	16	U
Ventral putamen	L	-20.4, 9.6, -9.6	4.55	$< 10^{-3}$	22	-
Nucleus accumbens	L	-9.6, 7.2, -9.6	5.11	$< 10^{-4}$	28	CP
Nucleus accumbens	R	6, 3.6, -8.4	5.20	$< 10^{-4}$	14	-
Posterior cingulate cortex	L	-15.6, -33.6, 50.4	4.42	$< 10^{-3}$	14	-

Reaction time						
Medial frontal cortex	R	7.2, 31.2, 43.2	5.13	$< 10^{-4}$	39	-
Medial frontal cortex	L	-4.8, 26.4, 40.8	3.66	10^{-3}	13	-
Dorsal anterior cingulate cortex	L	-14.4, 25.2, 26.4	6.00	10^{-5}	77	-
Medial frontal cortex	R	15.6, 9.6, 58.8	3.50	0.002	11	-
Medial frontal cortex	R	16.8, 3.6, 70.8	5.95	10^{-5}	98	-
Medial frontal cortex	B	-2.4, 2.4, 46.8	9.69	$< 10^{-7}$	3476	CP
Medial frontal cortex	L	-2.4, 2.4, 37.2	4.13	$< 10^{-3}$	10	-
Medial frontal cortex	R	12, -6, 62.4	3.93	$< 10^{-3}$	14	-
Medial frontal cortex	L	-8.4, -7.2, 73.2	7.20	$< 10^{-5}$	134	-
Medial frontal cortex	R	7.2, -8.4, 74.4	3.77	$< 10^{-3}$	14	-
Poor learners (*n* = 5)			t_4			
Value						
Ventral putamen	R	18, 7.2, -10.8	6.50	$< 10^{-3}$	11	-
Dorsal putamen	R	22.8, 7.2, -4.8	10.89	$< 10^{-4}$	11	-
Ventral putamen	L	-18, 1.2, -13.2	13.07	$< 10^{-4}$	21	-
Reaction time						
Medial frontal cortex	R	8.4, 13.2, 39.6	22.98	10^{-5}	18	-
Medial frontal cortex	L	-4.8, 1.2, 54	16.92	$< 10^{-4}$	18	-
Medial frontal cortex	L	-7.2, -7.2, 67.2	10.15	$< 10^{-3}$	27	-

Table S17. Neural substrates of the RL framework: 7-T Color/Motion version. This table is related to Figures 9 and S6 and Table S18.

Variable<br>Region of interest	3-T Face/House			7-T Color/Motion		
	All	Good	Poor	All	Good	Poor
n	40	31	9	21	16	5
Reward-prediction error						
Striatum	U	U	U	U	U	-
Dopaminergic midbrain	-	U	-	-	U	-
Set of 7 regions of interest	S	S	U	U	S	-
L Anterior caudate nucleus (-8, 18, -8)	CP	cP	c	-	-	-
R Nucleus accumbens (8, 12, -4)	-	-	-	CP	CP	-
R Ventral putamen (18, 12, -12)	CP	-	-	-	-	-
L Nucleus accumbens (-12, 10, -6)	CP	CP	Cp	-	p	-
R Dorsal putamen (28, 6, 0)	CP	CP	-	-	-	-
L Dorsal caudate nucleus (-18, 2, 16)	-	CP	-	-	-	-
L Substantia nigra (-10, -14, -12)	-	-	-	-	-	-
Value						
Ventromedial prefrontal cortex	U	U	U	U	U	-
Striatum	U	U	-	U	U	U
Posterior cingulate cortex	U	U	U	U	U	-
Set of 4 regions of interest	S	S	-	S	S	-
B Ventromedial prefrontal cortex (0, 46, -8)	CP	CP	-	U	CP	-
R Nucleus accumbens (10, 16, -6)	CP	CP	-	C	-	-
L Nucleus accumbens (-10, 10, -6)	-	-	-	C	CP	-
B Posterior cingulate cortex (-2, -34, 38)	Cp	cP	-	-	-	-
Reaction time						
Medial frontal cortex	U	U	U	U	U	U
B Medial frontal cortex (0, 12, 48)	CP	CP	CP	CP	CP	-

Table S18. Neural substrates of the RL framework: Summary. The first portion of fMRI analyses across data sets and participant groups (i.e., "All", "Good", and "Poor" learners) are summarized for the RL framework that serves as the foundation of the GRL model. Regions of interest (ROIs) were informed by prior studies modeling the reward-prediction error, value, and reaction time. Initially, broader exploratory ROIs were defined anatomically and tested for uncorrected results ("U") ($p < 0.005$). For RPE and value signals, coordinate-based ROIs were first tested collectively via SVC at the set level ("S") ($p_{FWE} < 0.05$). Post-hoc tests followed for individual ROIs via SVC at the cluster level ("C") or the peak level ("P") ($p_{FWE} < 0.05$); marginally significant ("s", "c", or "p" in lower case) ($0.05 < p_{FWE} < 0.10$) or uncorrected ("U") ($p < 0.005$) results are listed as well if the most stringent threshold for SVC was not attained. Left ("L"), right ("R"), and bilateral ("B") refer to hemispheres for each ROI. The conventions for displaying this table also apply for Tables S21, S22, and S25. This table is related to Figures 8, 9, and S6 and Tables S16 and S17.

Group (3-T Face/House)<br>Variable<br>Region	H	*x*, *y*, *z*	t_{df}	*p*	*k*	SVC
All learners (*n* = 40)			t_{38}			
RPE x State generalization						
Anterior caudate nucleus	R	14, 26, 6	3.98	10^{-4}	19	-
Dorsal caudate nucleus	R	12, 14, 18	4.52	$< 10^{-4}$	24	-
Anterior caudate nucleus	L	-16, 12, 10	5.30	$< 10^{-5}$	40	-
Posterior putamen	L	-18, -2, -10	5.06	$< 10^{-5}$	76	-
Posterior putamen	L	-22, -4, 12	4.19	$< 10^{-4}$	52	CP
Posterior caudate nucleus	R	16, -12, 26	5.33	$< 10^{-5}$	122	-
Substantia nigra	R	8, -12, -10	3.32	$< 10^{-3}$	20	-
Substantia nigra	L	-12, -16, -6	3.36	$< 10^{-3}$	16	U
Substantia nigra	R	14, -24, -8	4.56	$< 10^{-4}$	16	-
Hippocampus	L	-18, -16, -22	3.73	$< 10^{-3}$	17	-
Hippocampus	L	-28, -16, -24	3.95	$< 10^{-3}$	11	U
Hippocampus	R	30, -16, -20	4.25	$< 10^{-4}$	33	CP
RPE x Action generalization						
Hippocampus	R	34, -14, -24	4.06	10^{-4}	10	-
Good learners (*n* = 31)			t_{29}			
RPE x State generalization						
Dorsal caudate nucleus	R	6, 8, 14	3.86	$< 10^{-3}$	28	-
Dorsal caudate nucleus	R	14, 4, 10	3.41	$< 10^{-3}$	18	-
Dorsal putamen &<br>Dorsal caudate nucleus	L	-10, -6, -6	4.71	$< 10^{-4}$	85	-
Posterior caudate nucleus	R	18, -12, 26	4.63	$< 10^{-4}$	45	-
Hippocampus	L	-30, -14, -22	3.84	$< 10^{-3}$	13	cp
Hippocampus	L	-26, -36, 0	5.14	$< 10^{-5}$	27	-

Hippocampus	R	20, -40, 8	3.68	$< 10^{-3}$	14	-
RPE x Action generalization						
Anterior caudate nucleus	R	14, 18, 12	4.53	$< 10^{-4}$	10	-
Posterior putamen	L	-32, -2, -10	4.85	$< 10^{-4}$	24	-
Hippocampus	R	34, -16, -24	3.55	$< 10^{-3}$	12	-
Poor learners ($n = 9$)			t_7			
RPE x State generalization						
Dorsal caudate nucleus	L	-12, 10, 4	4.88	$< 10^{-3}$	35	-
Ventral putamen	L	-16, 0, -12	11.13	$< 10^{-5}$	60	-
Posterior putamen	L	-30, -4, -10	5.27	$< 10^{-3}$	15	-
Substantia nigra	R	8, -6, -12	4.44	0.002	13	-
Posterior caudate nucleus	R	14, -14, 22	6.02	$< 10^{-3}$	53	-
Hippocampus	R	28, -18, -20	5.47	$< 10^{-3}$	13	cp
RPE x Action generalization						
Dorsal caudate nucleus	R	14, 12, 2	6.86	10^{-4}	28	c
Dorsal putamen & Insular cortex	L	-34, 8, 8	6.43	$< 10^{-3}$	56	-
Dorsal caudate nucleus	R	18, -2, 20	7.52	$< 10^{-4}$	46	-
Posterior putamen	L	-32, -8, -2	6.56	$< 10^{-3}$	15	-
Posterior putamen	R	28, -10, -4	8.77	$< 10^{-4}$	35	-
Hippocampus	L	-36, -34, -6	8.92	$< 10^{-4}$	66	-
Hippocampus	L	-26, -42, -6	4.65	10^{-3}	21	-
Nonlearners ($n = 7$)			t_5			
RPE x State generalization						
Anterior caudate nucleus	R	18, 22, 6	7.74	$< 10^{-3}$	15	-
Dorsal putamen	R	26, 10, 6	4.71	0.003	11	U
RPE x Action generalization						

Dorsal caudate nucleus	L	-16, 2, 6	6.53	$< 10^{-3}$	23	-
Hippocampus	L	-20, -10, -26	9.52	10^{-4}	12	-

Table S19. Neural substrates of the GRL model: 3-T Face/House version. This table is related to Figure 10 and Table S21.

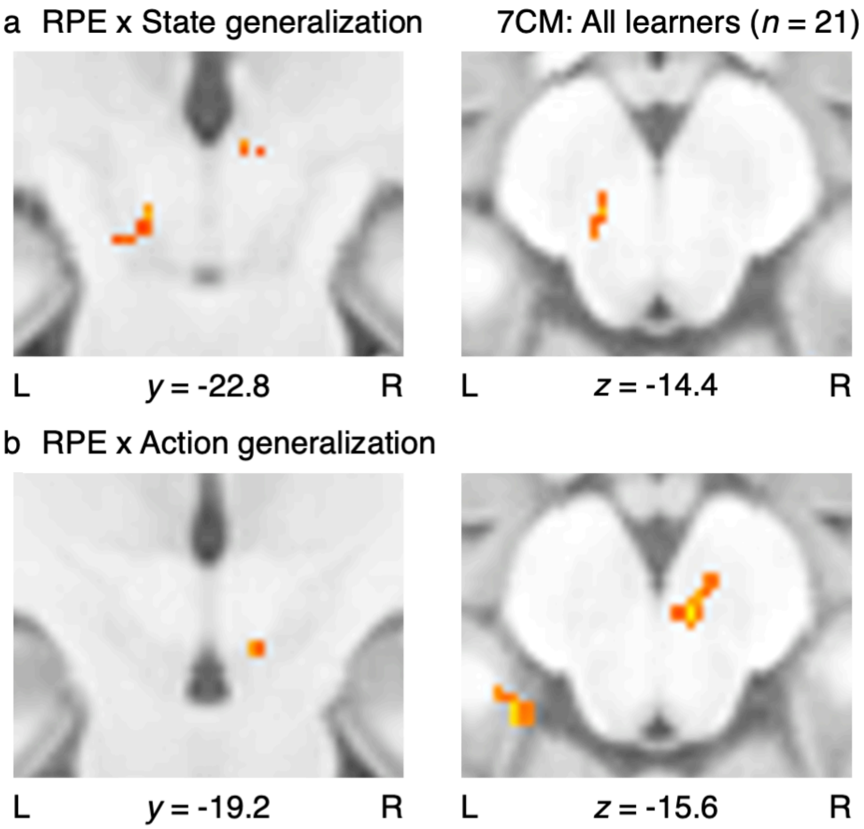
a RPE x State generalization
7CM: All learners (n = 21)
L
y = -22.8
R
L
z = -14.4
R
b RPE x Action generalization
L
y = -19.2
R
L
z = -15.6
R

Figure S7. Neural substrates of the GRL model: 7-T Color/Motion version (Dopaminergic midbrain). (a) At 7 T, interaction effects between RPE signals and state generalization were localized to both the SN and the ventral tegmental area (VTA) ($p < 0.005$). **(b)** Interaction effects between RPE signals and action generalization were likewise observed in both the SN and the VTA ($p < 0.005$). This figure is related to Figure 10 and Tables S20 and S21.

Group (7-T Color/Motion)<br>Variable<br>Region	H	*x*, *y*, *z*	t_{df}	*p*	*k*	SVC
All learners (*n* = 21)			t_{19}			
RPE x State generalization						
Anterior caudate nucleus	L	-10.8, 19.2, -7.2	4.19	$< 10^{-3}$	13	c
Nucleus accumbens	R	12, 14.4, -6	4.79	$< 10^{-4}$	19	CP
Dorsal caudate nucleus	L	-8.4, 13.2, 10.8	4.19	$< 10^{-3}$	15	-
Dorsal caudate nucleus	R	6, 12, 3.6	4.78	$< 10^{-4}$	26	-
Ventral putamen	L	-15.6, 12, -13.2	4.31	$< 10^{-3}$	16	-
Dorsal caudate nucleus	R	16.8, 10.8, 14.4	4.78	$< 10^{-4}$	20	-
Nucleus accumbens	R	10.8, 4.8, -10.8	5.23	$< 10^{-4}$	17	-
Dorsal caudate nucleus	L	-8.4, 3.6, 15.6	4.41	$< 10^{-3}$	29	-
Posterior putamen	L	-30, -13.2, -7.2	4.17	$< 10^{-3}$	14	-
Posterior putamen	R	32.4, -21.6, 1.2	5.71	$< 10^{-5}$	12	-
Substantia nigra &<br>Ventral tegmental area	L	-7.2, -22.8, -14.4	4.53	10^{-4}	15	-
RPE x Action generalization						
Anterior caudate nucleus	L	-4.8, 14.4, -2.4	4.23	$< 10^{-3}$	13	-
Dorsal caudate nucleus	L	-8.4, 13.2, 15.6	5.15	$< 10^{-4}$	81	-
Dorsal caudate nucleus	L	-9.6, 6, 6	4.83	$< 10^{-4}$	17	-
Nucleus accumbens	R	12, 4.8, -10.8	3.69	$< 10^{-3}$	10	-
Ventral tegmental area &<br>Substantia nigra	R	3.6, -21.6, -15.6	4.84	$< 10^{-4}$	13	-
Good learners (*n* = 16)			t_{14}			
RPE x State generalization						
Anterior caudate nucleus	R	9.6, 19.2, -2.4	5.26	$< 10^{-4}$	18	-
Nucleus accumbens	L	-10.8, 16.8, -9.6	4.80	10^{-4}	17	Cp

Anterior caudate nucleus	L	-4.8, 14.4, 0	9.57	$< 10^{-7}$	28	-
Nucleus accumbens	R	10.8, 14.4, -6	5.19	$< 10^{-4}$	16	CP
Anterior caudate nucleus	R	4.8, 13.2, 2.4	6.01	$< 10^{-4}$	23	U
Dorsal caudate nucleus	L	-8.4, 13.2, 15.6	5.12	$< 10^{-4}$	78	-
Dorsal caudate nucleus	R	15.6, 12, 13.2	3.93	$< 10^{-3}$	15	-
Nucleus accumbens	L	-7.2, 10.8, -4.8	4.47	$< 10^{-3}$	13	U
Dorsal caudate nucleus	L	-7.2, 6, 4.8	5.03	$< 10^{-4}$	14	-
Nucleus accumbens	R	10.8, 4.8, -10.8	7.27	$< 10^{-5}$	24	-
Dorsal putamen	L	-26.4, 3.6, 14.4	4.80	10^{-4}	16	-
Posterior putamen	R	26.4, -2.4, -6	4.51	$< 10^{-3}$	12	-
Posterior putamen	R	31.2, -6, 4.8	5.88	$< 10^{-4}$	11	-
Hippocampus	L	-22.8, -18, -20.4	4.72	$< 10^{-3}$	13	-
Hippocampus	L	-28.8, -22.8, -12	5.07	$< 10^{-4}$	22	C
Hippocampus	R	25.2, -27.6, -10.8	5.01	$< 10^{-4}$	28	-
RPE x Action generalization						
Anterior caudate nucleus	L	-6, 18, -1.2	5.76	$< 10^{-4}$	18	-
Anterior caudate nucleus	L	-8.4, 13.2, 10.8	4.46	$< 10^{-3}$	14	-
Dorsal caudate nucleus	L	-8.4, 13.2, 15.6	5.75	$< 10^{-4}$	67	-
Dorsal caudate nucleus	R	4.8, 12, 2.4	5.78	$< 10^{-4}$	17	-
Dorsal caudate nucleus	L	-8.4, 6, 6	4.90	10^{-4}	12	-
Nucleus accumbens	R	10.8, 4.8, -12	5.70	$< 10^{-4}$	19	-
Posterior putamen	R	32.4, -3.6, 4.8	4.48	$< 10^{-3}$	13	-
Poor learners (n = 5)			t_3			
RPE x State generalization						
Dorsal caudate nucleus	R	9.6, 7.2, 14.4	13.39	$< 10^{-3}$	12	-
Posterior putamen	L	-24, -13.2, -1.2	26.55	$< 10^{-4}$	19	-

Table S20. Neural substrates of the GRL model: 7-T Color/Motion version. This table is related to Figures 10 and S7 and Table S21.

Variable<br>Region of interest	3-T Face/House			7-T Color/Motion		
	All	Good	Poor	All	Good	Poor
n	40	31	9	21	16	5
RPE x State generalization						
Striatum	U	U	U	U	U	U
Dopaminergic midbrain	U	-	U	U	-	-
Set of 7 regions of interest	S	-	-	s	S	-
L Anterior caudate nucleus (-8, 18, -8)	-	-	-	c	Cp	-
R Nucleus accumbens (8, 12, -4)	-	-	-	CP	CP	-
R Ventral putamen (18, 12, -12)	-	-	-	-	-	-
L Nucleus accumbens (-12, 10, -6)	-	-	-	-	U	-
R Dorsal putamen (28, 6, 0)	-	-	-	-	-	-
L Dorsal caudate nucleus (-18, 2, 16)	CP	-	-	-	-	-
L Substantia nigra (-10, -14, -12)	U	-	-	-	-	-
RPE x Action generalization						
Striatum	-	U	U	U	U	-
Dopaminergic midbrain	-	-	-	U	-	-
Set of 7 regions of interest	-	-	U	-	-	-
L Anterior caudate nucleus (-8, 18, -8)	-	-	-	-	-	-
R Nucleus accumbens (8, 12, -4)	-	-	c	-	-	-
R Ventral putamen (18, 12, -12)	-	-	-	-	-	-
L Nucleus accumbens (-12, 10, -6)	-	-	-	-	-	-
R Dorsal putamen (28, 6, 0)	-	-	-	-	-	-
L Dorsal caudate nucleus (-18, 2, 16)	-	-	-	-	-	-
L Substantia nigra (-10, -14, -12)	-	-	-	-	-	-

Table S21. Neural substrates of the GRL model: Summary (Basal ganglia). The second portion of the fMRI analyses are first summarized for the basal ganglia as further validation of the GRL model. As these effects lack precedent, the ROIs (as before) originated from a prior study that modeled the RPE without including any effects of generalization. This table is related to Figures 10 and S7 and Tables S19 and S20.

Variable<br>Region of interest	3-T Face/House			7-T Color/Motion		
	All	Good	Poor	All	Good	Poor
n	40	31	9	21	16	5
RPE x State generalization						
Hippocampus	U	U	U	-	U	-
Set of 2 regions of interest	S	U	U	-	U	-
L Hippocampus (-28, -18, -16)	U	cp	-	-	C	-
R Hippocampus (28, -18, -16)	CP	-	cp	-	-	-
RPE x Action generalization						
Hippocampus	U	U	U	-	-	-
Set of 2 regions of interest	-	-	-	-	-	-
L Hippocampus (-28, -18, -16)	-	-	-	-	-	-
R Hippocampus (28, -18, -16)	-	-	-	-	-	-

Table S22. Neural substrates of the GRL model: Summary (Hippocampus). This qualitative summary of the second portion of the fMRI analyses examines the hippocampus. This table is related to Figure 10 and Tables S19 and S20.

Group (3-T Face/House)<br>Variable<br>Region	H	*x*, *y*, *z*	t_{df}	*p*	*k*	SVC
All learners (*n* = 40)			t_{38}			
RPE x Learning rate						
Dorsal caudate nucleus	R	10, 4, 18	3.76	$< 10^{-3}$	12	-
Poor learners (*n* = 9)			t_{7}			
RPE x Learning rate						
Ventral tegmental area & Substantia nigra	R	6, -18, -12	7.43	$< 10^{-4}$	17	-
Nonlearners (*n* = 7)			t_{5}			
RPE x Learning rate						
Dorsal caudate nucleus	R	18, -4, 26	7.54	$< 10^{-3}$	11	-

Table S23. Neural substrates of the learning rate: 3-T Face/House version. This table is related to Table S25.

Group (7-T Color/Motion)<br>Variable<br>Region	H	*x*, *y*, *z*	t_{df}	*p*	*k*	SVC
All learners (*n* = 21)			t_{19}			
RPE x Learning rate						
Dorsal caudate nucleus	R	12, -1.2, 14.4	4.56	10^{-4}	21	-
Posterior putamen	R	27.6, -12, 13.2	4.68	$< 10^{-4}$	11	-
Good learners (*n* = 16)			t_{14}			
RPE x Learning rate						
Dorsal caudate nucleus	R	15.6, -1.2, 14.4	5.70	$< 10^{-4}$	22	-
Posterior putamen	R	27.6, -12, 13.2	4.46	$< 10^{-3}$	12	-
Poor learners (*n* = 5)			t_{3}			
RPE x Learning rate						
Ventral tegmental area	L	-7.2, -15.6, -10.8	12.18	$< 10^{-3}$	13	C

Table S24. Neural substrates of the learning rate: 7-T Color/Motion version. This table is related to Table S25.

Variable<br>Region of interest	3-T Face/House			7-T Color/Motion		
	All	Good	Poor	All	Good	Poor
n	40	31	9	21	16	5
RPE x Learning rate						
Striatum	U	-	-	U	U	-
Dopaminergic midbrain	-	-	U	-	-	U
Set of 7 regions of interest	-	-	-	-	-	U
L Anterior caudate nucleus (-8, 18, -8)	-	-	-	-	-	-
R Nucleus accumbens (8, 12, -4)	-	-	-	-	-	-
R Ventral putamen (18, 12, -12)	-	-	-	-	-	-
L Nucleus accumbens (-12, 10, -6)	-	-	-	-	-	-
R Dorsal putamen (28, 6, 0)	-	-	-	-	-	-
L Dorsal caudate nucleus (-18, 2, 16)	-	-	-	-	-	-
L Substantia nigra (-10, -14, -12)	-	-	-	-	-	C
RPE x Learning rate						
Hippocampus	-	-	-	-	-	-
Set of 2 regions of interest	-	-	-	-	-	-
L Hippocampus (-28, -18, -16)	-	-	-	-	-	-
R Hippocampus (28, -18, -16)	-	-	-	-	-	-

Table S25. Neural substrates of the learning rate: Summary. The absence of overlap between specific effects of generalization and effects of learning performance indicates that the former are not confounded with the latter. This table is related to Tables S23 and S24.
